# Development of Bicyclo[3.1.0]hexane-Based A_3_ Receptor Ligands: Closing the Gaps in the Structure–Affinity Relationships

**DOI:** 10.3390/molecules27072283

**Published:** 2022-03-31

**Authors:** Jan Phillip Lemmerhirt, Andreas Isaak, Rongfang Liu, Max Kock, Constantin G. Daniliuc, Kenneth A. Jacobson, Laura H. Heitman, Anna Junker

**Affiliations:** 1 European Institute for Molecular Imaging (EIMI), University of Münster, Waldeyerstr. 15, 48149 Münster, Germany; janlemmerhirt@gmail.com (J.P.L.); a_isaa01@uni-muenster.de (A.I.); max.kock@web.de (M.K.); 2 Leiden Academic Centre for Drug Research (LACDR), Division of Medicinal Chemistry, Leiden University, Einsteinweg 55, 2333 CC Leiden, The Netherlands; r.liu@lacdr.leidenuniv.nl (R.L.); l.h.heitman@lacdr.leidenuniv.nl (L.H.H.); 3 Organisch-Chemisches Institut, University of Münster, Corrensstraße 40, 48149 Münster, Germany; constantin.daniliuc@uni-muenster.de; 4 Molecular Recognition Section, Laboratory of Bioorganic Chemistry, National Institute of Diabetes and Digestive and Kidney Diseases, National Institutes of Health, Bethesda, MD 20892, USA; kennethj@niddk.nih.gov

**Keywords:** Adenosine receptors, methanocarba, bicyclo[3.1.0]hexane, A_3_ receptors

## Abstract

The adenosine A_3_ receptor is a promising target for treating and diagnosing inflammation and cancer. In this paper, a series of bicyclo[3.1.0]hexane-based nucleosides was synthesized and evaluated for their P1 receptor affinities in radioligand binding studies. The study focused on modifications at 1-, 2-, and 6-positions of the purine ring and variations of the 5′-position at the bicyclo[3.1.0]hexane moiety, closing existing gaps in the structure–affinity relationships. The most potent derivative **30** displayed moderate A_3_AR affinity (K*i* of 0.38 μM) and high A_3_R selectivity. A subset of compounds varied at 5′-position was further evaluated in functional P2Y_1_R assays, displaying no off-target activity.

## 1. Introduction

**Figure 1:**
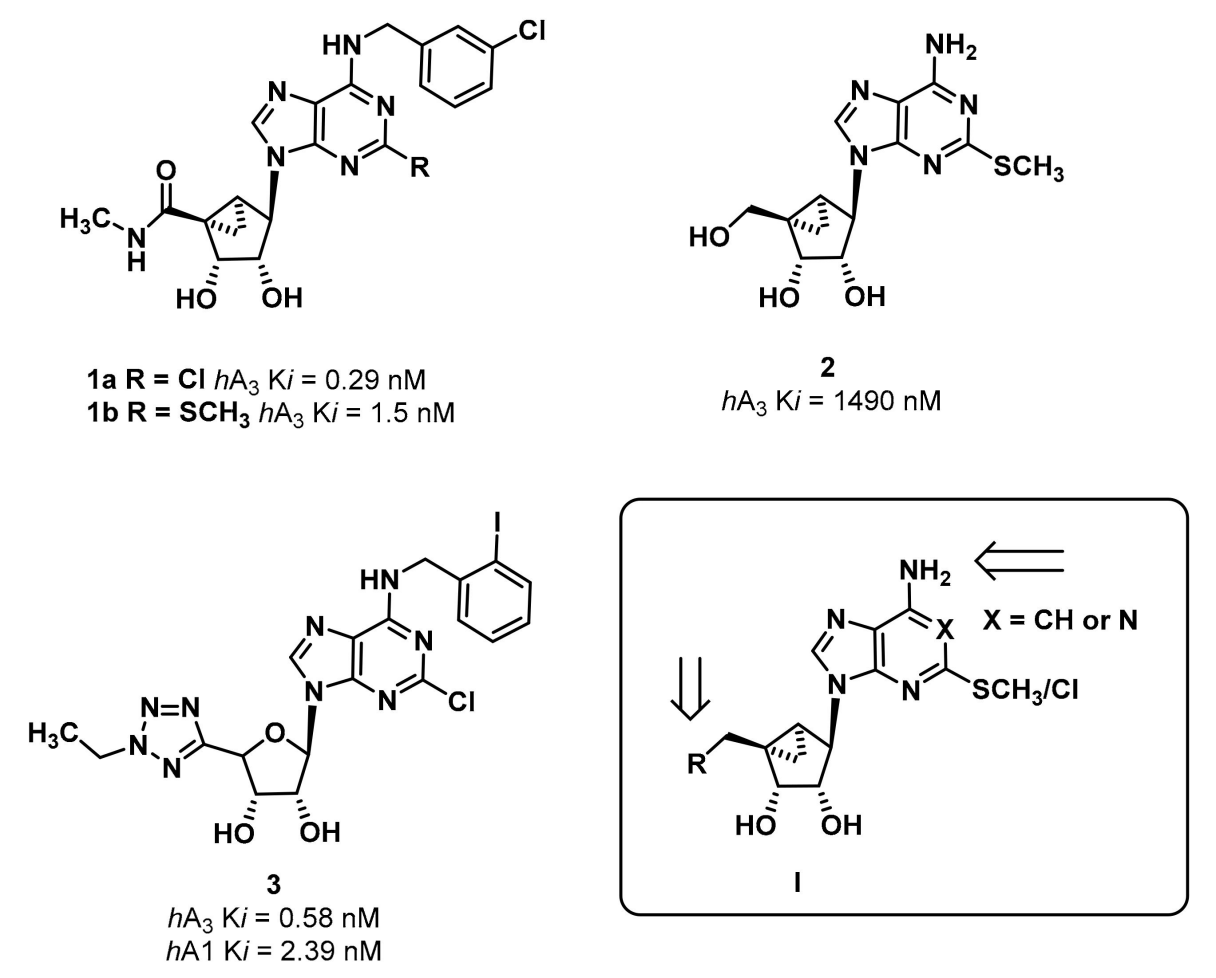
(N)-methanocarba-based A_3_ receptor antagonists **1a,b**, **2**, the tetrazole derivative **3** and general structure **I**.

The G protein-coupled adenosine (P1) receptors A_1_, A_2A_, A_2B_ and A_3_ play a central role in the complex mechanisms of purinergic signaling. In general, adenosine, the endogenous agonist at P1 receptors, exhibits protective functions as a response to organ stress and release of damage-associated-molecular pattern (DAMP) molecules such as e.g., ATP and S100 proteins [1,2,3]. Various P1 receptor agonists have been in clinical trials; to name a few, capadenoson (A_1_AR agonist) for the treatment of atrial fibrillation (NCT00568945) [4], apadenoson (A_2A_AR agonist) for the SPECT-myocardial perfusion imaging (NCT01313572), the A_3_ receptor agonists namodenoson in phase III for liver cancer (NCT04697810) [5,6], and piclidenoson (IB-MECA) for the treatment of psoriasis (NCT03168256), rheumatoid arthritis, and most recently, COVID-19 infections (NCT04333472) [7]. We are particularly interested in targeting the A_3_ receptor due to its high overexpression in inflammatory and cancer cells compared to its low expression levels in healthy cells, thus making it a potentially promising therapeutic and diagnostic target [8,9,10]. The introduction of the bicyclo[3.1.0]hexane scaffold, also known as (N)-methanocarba (N for North), in place of the furanose ring of nucleoside agonists is known to increase the A_3_ receptor (A_3_AR) potency and selectivity in comparison to other adenosine receptor subtypes [11,12]. In 2005 Jacobson et al. reported compounds **1a** and **1b** as highly potent A_3_ receptor agonists [13] and most recently, the synthesis of S-thioether (N)-methanocarba adenosine derivatives such as compound **2** (Figure 1) [14]. We were interested in exploring these scaffolds further through various substitutions at 6-position of the purine ring (purine numbering), the introduction of the 1-deazapurine scaffold, and variations of the 5′-position (ribose numbering) at the methanocarba moiety (Figure 1, general structure **I**). Jacobson et al. have already established the methyl and ethyl carboxamides as highly efficient substituents at the 5′-position. There are only a few reports on introducing other functional moieties at the 5′-position of adenosine receptor ligands, one of them being the tetrazole compound **3** as a highly potent dual A_1_AR and A_3_AR ligand [15]. However, the introduction of other, in particular acidic, functional groups at the 5′-position was never investigated. Therefore, we decided to combine the *(*N)-methanocarba moiety (providing A_3_AR preference [12]) with various functional groups at the 5′-position to develop novel adenosine receptor ligands.

## 2. Results and Discussion

**Scheme 1:**
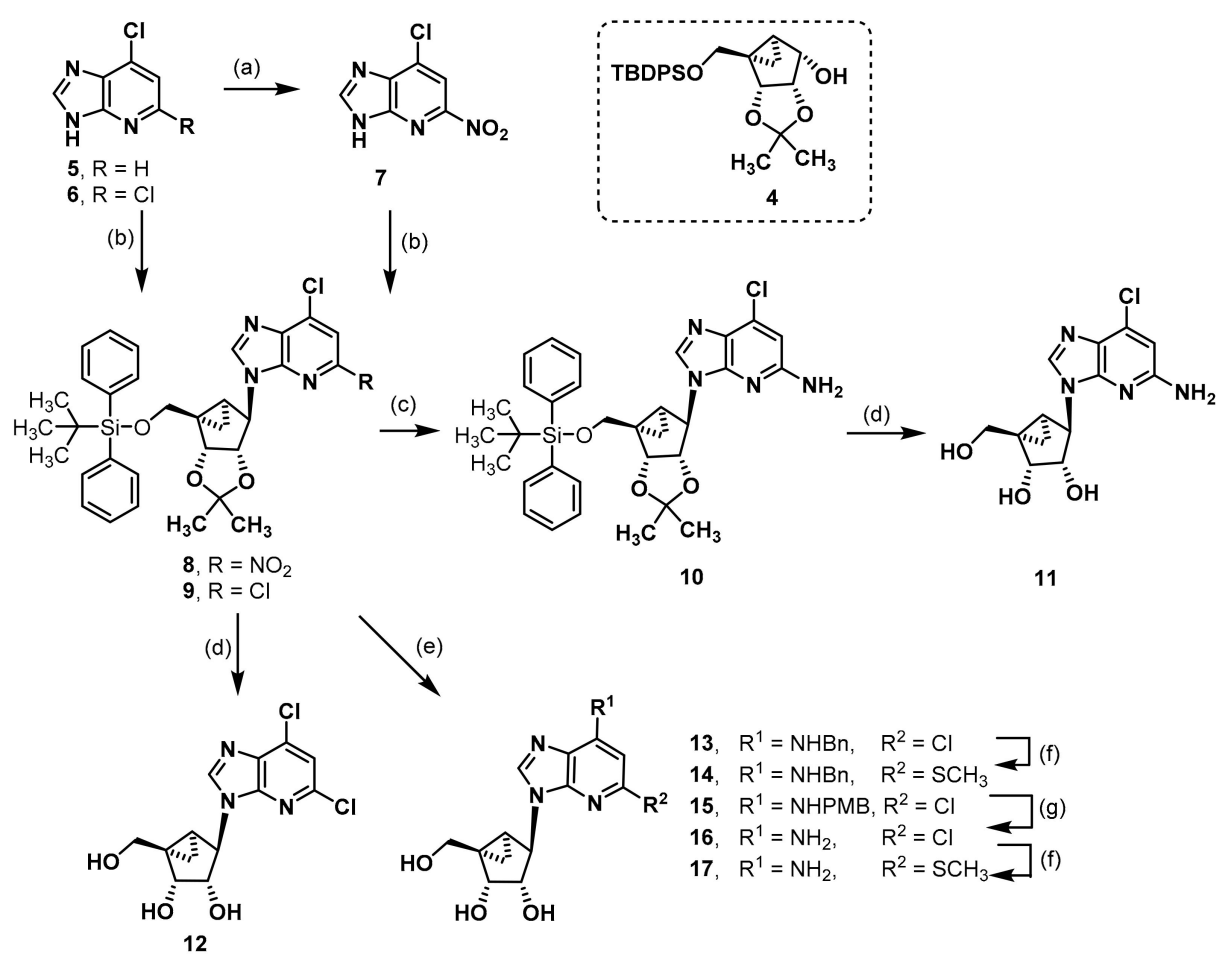
Synthesis of 1-deazapurine derivatives **11**–**17**. Reagents and conditions: (a) (1) Boc_2_O, DMAP, CH_2_Cl_2_, RT. (2) TBAN, TFAA, CH_2_Cl_2_, CH_3_OH, rt ⭢ reflux. (b) compound **4**, DIAD, PPh_3_, THF, rt ⭢ 70 °C. (c) Na_2_S_2_O_4_, CH_3_OH, H_2_O, rt. (d) TFA, CH_3_OH, H_2_O, 70 °C. (e) Benzylamine, DIPEA, NMP, 200 °C, then TFA, CH_3_OH, H_2_O, 70 °C. or *p*-methoxybenzylamine, DIPEA, NMP, 200 °C, then TFA, CH_3_OH, H_2_O, 70 °C. (f) NaSCH_3_, DMF, 90 °C, then TFA, CH_3_OH, H_2_O, 70 °C. (g) DDQ, H_2_O, CH_2_Cl_2_, rt, then TFA, CH_3_OH, H_2_O, 70 °C.

The synthesis of the bicyclo[3.1.0]hexane scaffold followed the reported procedure by Michel et al. [16], starting with D-ribose and leading to the TBDPS-protected bicyclo[3.1.0]hexan alcohol **4** as a central building block in 9 consecutive steps (see Supplementary Materials Scheme S1). First, we decided to explore the role of the nitrogen atom at the 1-position of the purine ring. Nitration of 6-chloro-1-deazapurine has led selectively to the formation of 2-nitro derivative **7**. Mitsunobu reaction of either 2,6-dichloro-1-deazapurine (**6**) or 6-chloro-2-nitro-1-deazapurine (**7**) with the methanocarba building block **4** had led to the formation of the protected nucleoside derivatives **8** and **9**, respectively, that were subsequently varied further at the 2-position of the purine ring through the introduction of either amino or methylthio groups (Scheme 1). The exocyclic amine at 6-position was introduced in a reaction of the 6-chloro derivative **9** and benzylamine (for compounds **13** and **14**) or para-methoxybenzyl amine (PMB, for compound **15**). Cleavage of the PMB group led to the derivatives **16** and **17** bearing a free exocyclic amine.

**Scheme 2:**
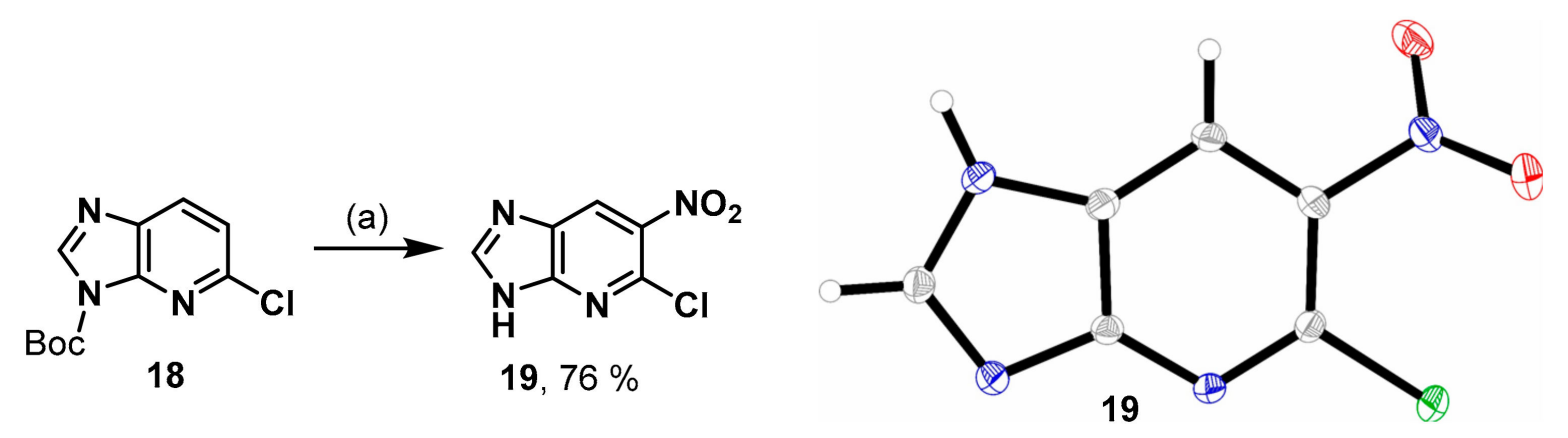
Nitration of Boc-protected 2-chloro-1-deazapurine (**18**) Reagents and conditions: (a) TBAN, TFAA, CH_2_Cl_2_, rt ⭢ reflux. Molecular structure of compound **19**. Thermal ellipsoids are depicted at 30% probability. CCDC number: 2157452.

The attempt of introducing the nitro group at the Boc-protected 2-chloro-1-deazapurine (**18**), in order to introduce the electron-withdrawing nitro group at 6-position, has led to the formation of one single compound, the 2-chloro-1-nitro-1-deazapurine (**19**), in 76% yield and not the desired 6-nitro derivative (purine numbering). The position of the nitration was additionally proven by an X-ray structure of compound **19** (Scheme 2).

**Scheme 3:**
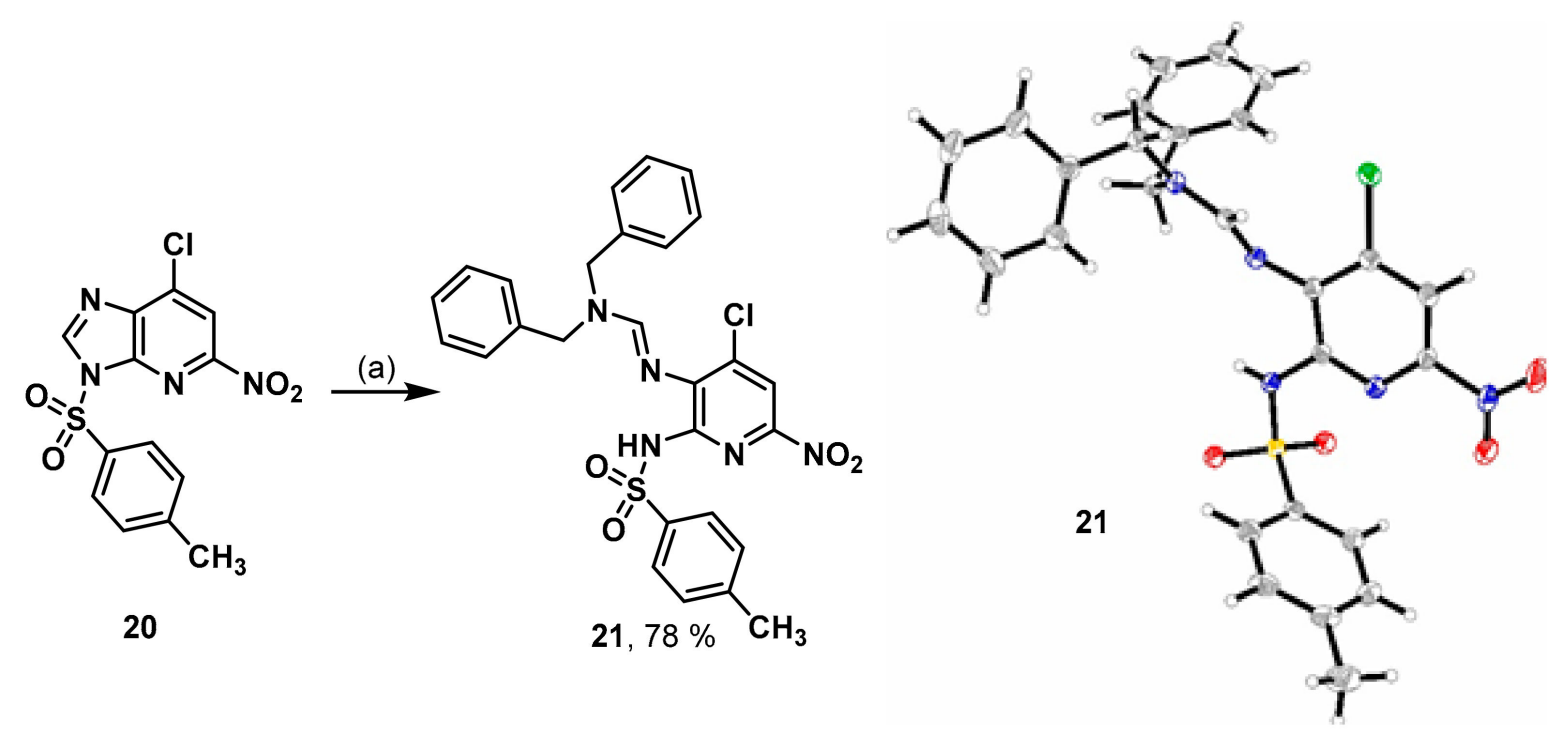
Reaction of dibenzyl amine with compound **20**. Reagents and conditions: (a) dibenzylamine, CH_2_Cl_2_, RT. Molecular structure of compound **21**. Thermal ellipsoids are depicted at 30% probability. CCDC number: 2157453.

The reaction of the protected 6-chloro-2-nitro nucleoside **8** with dibenzylamine was sluggish; therefore, to synthesize the *N*,*N*-dibenzyl-1-deaza derivatives, we envisaged the introduction of the dibenzyl group at the tosyl-protected 6-chloro-2-nitro-1-deazapurine **20** followed by subsequent cleavage of the tosyl group and a Mitsunobu reaction with compound **4**. Interestingly, the reaction of dibenzylamine with deazapurine **20** provided selectively the ring-opened product **21** in 78% yield. Due to the strong electron-withdrawing effect of the tosyl group, the dibenzylamine was able to perform a nucleophilic attack at the 8-position of the purine scaffold. The structure of compound **21** was additionally confirmed by X-ray crystal structure analysis (Scheme 3). Since the reaction of nucleoside **8** bearing a nitro group at the 2-position with dibenzylamine has led to the formation of various side-products, the synthesis of dibenzyl derivatives was skipped, and the nitro group was subsequently reduced to the primary amine function leading subsequently to the nucleoside **11**.

**Scheme 4:**
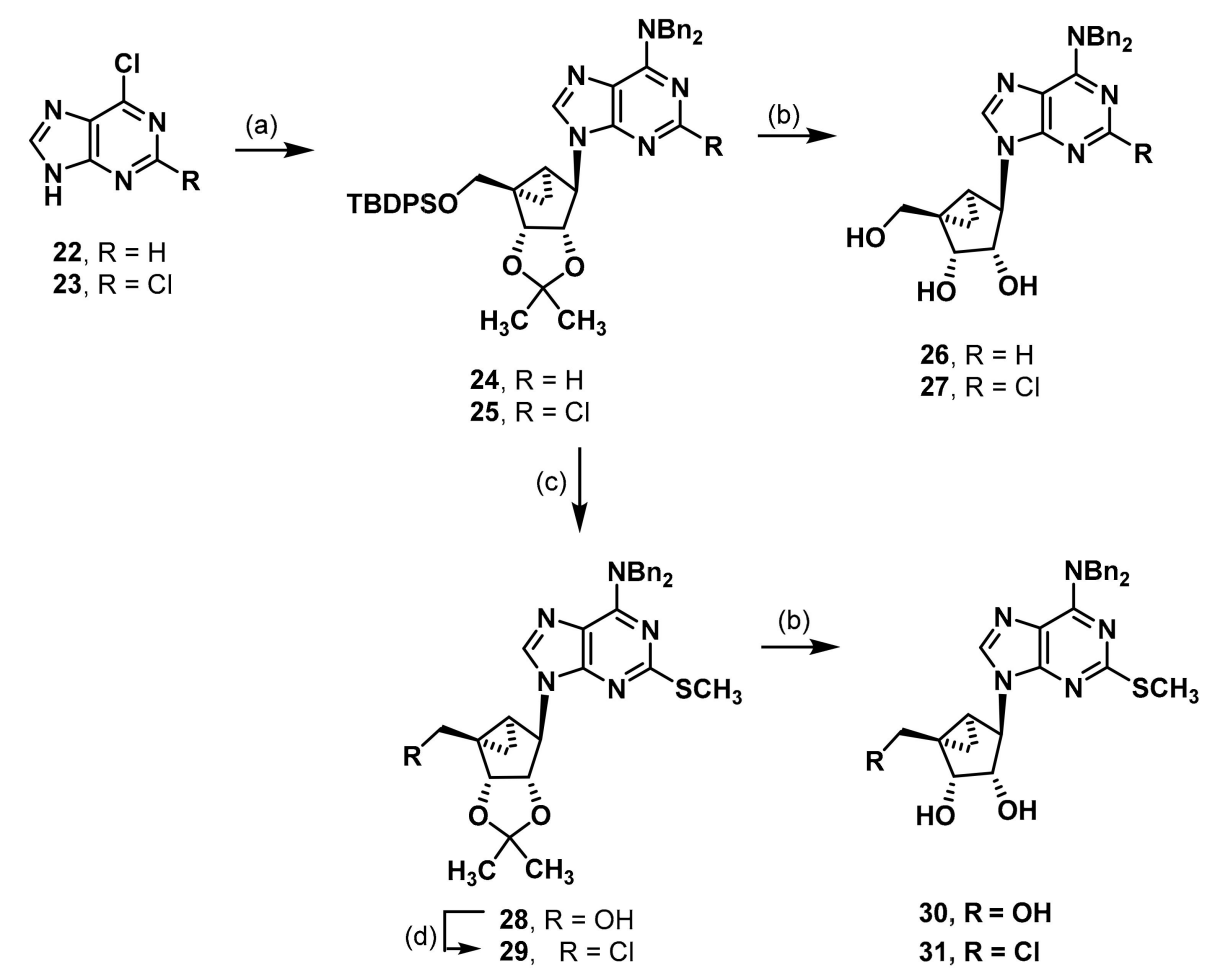
Synthesis of dibenzyl derivatives **26**, **27**, **30** and **31**. Reagents and conditions: (a) (1) Dibenzylamine, isopropanol, 90 °C. (2) Compound **4**, DIAD, PPh_3_ THF, 0 °C ⭢ RT. (b) TFA, CH_3_OH, H_2_O, 70 °C. (c) NaSCH_3_, DMF, 90 °C. (d) Cyanuric chloride, DMF, CH_2_Cl_2_, rt.

The purine derivatives **24** and **25**, bearing two benzyl groups, were prepared through the reaction of 6-chloropurines **22** and **23** with dibenzyl amine and subsequent Mitsunobu reaction of the methanocarba building block **4**, respectively. The methylthio group was introduced by reacting the protected 2-chloropurine nucleoside **25** with NaSCH_3_. Additionally, the 5′-hydroxy group was replaced by a chloride using cyanuric chloride. Cleavage of the acetonide and TBDPS groups has led to the formation of the respective nucleosides **26**, **27**, **30** and **31** in high yields (Scheme 4).

**Scheme 5:**
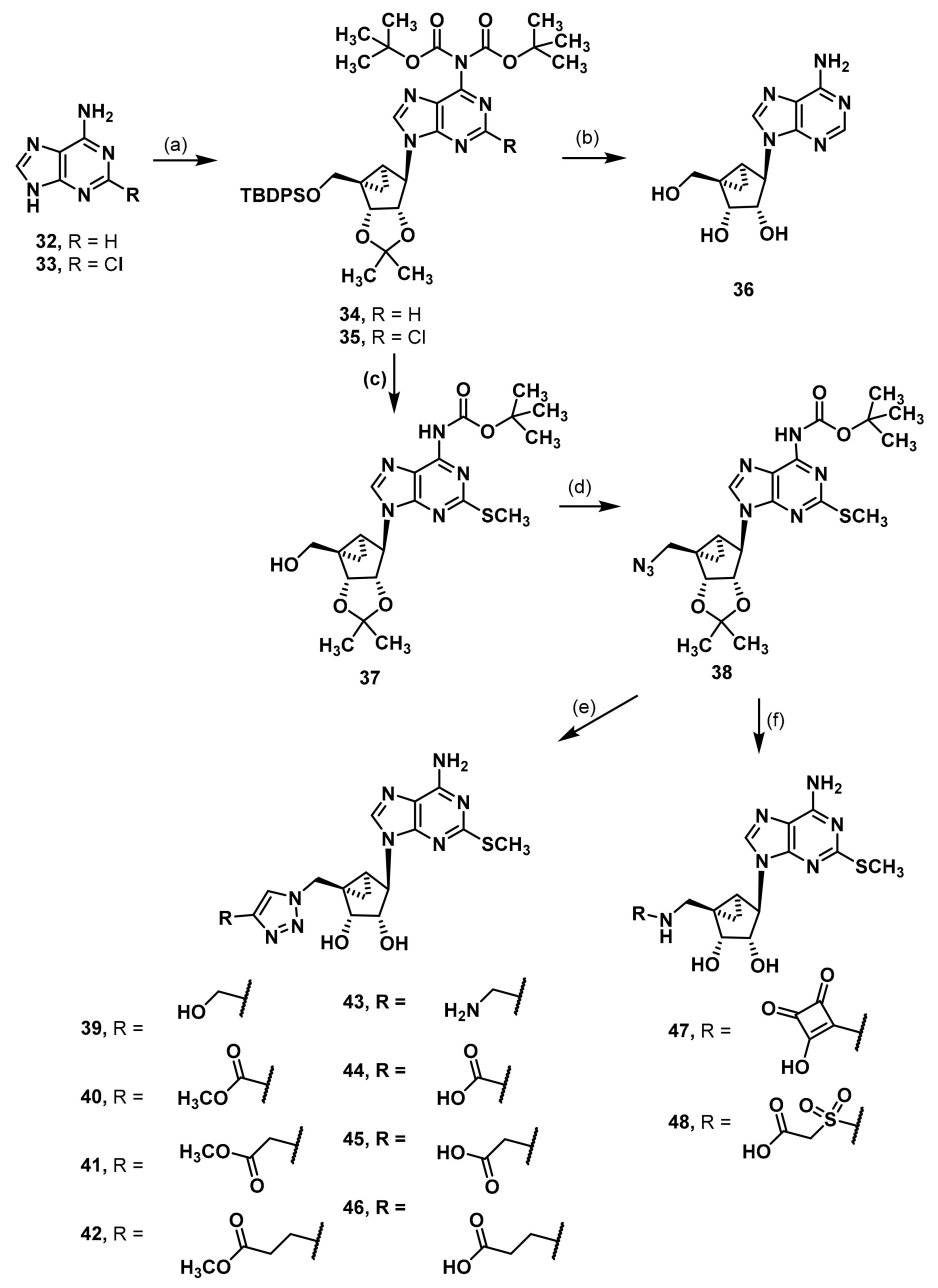
Synthesis of (N)-methanocarba adenosine **33** and derivatives modified at the 5′-position **39**–**48**. (a) (1) Boc_2_O, DMAP, THF, rt., then sat. NaHCO_3_ solution, CH_3_OH, 50 °C. (2) Compound **4**, DIAD, PPh_3_, THF, 0 °C ⭢ rt. (b) TFA, CH_3_OH, H_2_O, 70 °C. (c) NaSCH_3_, DMF, 90 °C. (d) (1) Tosyl chloride, Et_3_N, DMAP, CH_2_Cl_2_, RT. (2) NaN_3_, DMF, 70 °C. (e) Appropriate alkyne, Cu (II) acetylacetonate, sodium ascorbate, *tert*-butanol, H_2_O, rt, then TFA, CH_3_OH, H_2_O, 70 °C. (f) (1) Pd/C, H_2_ 5 bar, CH_3_OH, rt. (2) For **47**: squaric acid dimethyl ester, Et_3_N, CH_2_Cl_2_, rt., then TFA, CH_3_OH, H_2_O, 70 °C. For **48**: (1) methyl 2-(chlorosulfonyl)acetate, Et_3_N, DMAP, CH_2_Cl_2_, RT, then TFA, CH_3_OH, H_2_O, 70 °C. (2) NaOH, CH_3_CN, H_2_O, 70 °C.

Intrigued by the high A_3_AR affinity of compound **1b** and moderate affinity of compound **2**, we selected the 2-methylthio substituted adenine scaffold for the evaluation of the modifications at the 5′-position. Also, (N)-methanocarba adenine **36** should be prepared as a reference compound for the SAR studies. Hereby adenine (**32**) or 2-chloro adenine (**33**) were subjected to the Mitsunobu reaction. Subsequent cleavage of the protecting groups of compound **34** furnished (N)-methanocarba adenosine **36**, while the protected nucleoside **35** was used for the introduction of the methylthio group at the 2-position. Selective cleavage of the TBDPS protecting group and subsequent tosylation of the free alcohol and nucleophilic substitution of the tosylate led to the formation of the azide **38** as a central intermediate. Huisgen cycloaddition of the azide **38** with various alkynes and subsequent cleavage of the acetonide provided the triazole nucleosides **39**–**46** bearing neutral (**39**–**42**), basic (**43**), or acidic (**44**–**46**) functional groups. Reduction of the azide function using Pd/C, H_2_ led to the formation of an amine suitable for the reaction with squaric acid dimethyl ester or ethyl 2-(chlorosulfonyl)acetate to provide compounds **47** and **48**, respectively (Scheme 5).

The compounds were evaluated for their P1 receptor affinity in A_1_, A_2A_, A_2B_, and A_3_ receptor binding studies (Table 1). From all synthesized compounds, the (N)-methanocarba adenosine **36** is the only derivative displaying affinity to more than one P1 receptor. Compound **36** shows a preference for the A_3_ receptor subtype with a K*i* of 960 nM, 2- to 6-fold lower affinity towards A_2A_ and A_1_ receptors, respectively, and no affinity at the A_2B_ subtype. The nitrogen atom at 1-position is not required for A_3_ receptor affinity, as receptor binding appears to highly depend on substituents at 2- and 6-position. The derivatives **11** and **12** bearing a chloro substituent at 6-position show no P1 receptor affinity. The introduction of an amino group at the 6-position of the adenine ring as in compound **16** significantly increases the A_3_ receptor affinity (K*i* = 1.60 μM) while not showing any binding at other subtypes. Replacing the chloro with a methylthio group as in **17** leads to a loss of P1 receptor affinity. Interestingly, benzylation of the exocyclic amine as in compounds **13** and **14** restores the A_3_R affinity irrespective of the substituent at the 2-position. Extending the benzyl to a para-methoxybenzyl group as in **15** has no effect on A_3_R binding (K*i* = 0.50 μM). In the purine series, the dibenzylation of the exocyclic amine appears to work only in combination with the methylthio group (**30**, K*i* (A_3_R) = 0.38 μM); derivatives **26**, **27** and **31** were not potent at the A_3_ receptor. Most variations at the 5′-position were not tolerated. Only the triazole ester **42** displays a low A_3_R affinity of K*i* 6.35 μM. Considering the potential of the introduced moieties in compounds **39**–**48** to serve as potential bioisosteres of mono- and diphosphate groups, the compounds **39**–**48** were tested for their functional activity (agonistic and antagonistic) at P2Y_1_ receptors; none of the derivatives displayed any functional activity at P2Y_1_ receptors up to a concentration of 10 μM.

## 3. Conclusions

With the aim to further explore the SAR of (N)-methanocarba nucleosides at A_3_ receptor, a series of derivatives **11**–**17**, **26**, **27**, **30**, **31**, **36**, **39**–**48** varied at 1-, 2-, 6- and 5′-positions were prepared and evaluated for their affinity across all P1 receptor subtypes. The (N)-methanocarba adenosine **36** displayed affinity at A_1_, A_2A_, and A_3_ receptors combined with only moderate A_3_AR preference. The most potent compound **30**, bearing dibenzylamino group at the 6-position and methylthio at the 2-position, displayed high A_3_R selectivity. The presence of the nitrogen atom at the 1-position of the purine ring was not required for the A_3_AR affinity, consistent with a recent report on hypermodified (N)-methanocarba derivatives [15]. The introduction of larger moieties at the 5′-position led to a complete loss of A_3_AR affinity, except for the triazole ester **42** displaying low A_3_AR affinity. Further structural modifications such as e.g., benzylation of the exocyclic amine function might restore the affinity of the 5′-triazoles at the A_3_ receptor.

In conclusion, based on the multiple potential applications of (N)-methanocarba nucleosides as therapeutic agents [17,18], we have introduced new lead compounds that bind to the A_3_AR and can be further elaborated to increase affinity and selectivity.

## 4. Materials and Methods

### 4.1. Experimental Section

#### 4.1.1. Chemistry General

Unless otherwise noted, moisture-sensitive reactions were conducted under dry nitrogen. Flash column chromatography (fc): silica gel 60, 40–64 µm; parentheses include: diameter of the column, length of the column, fraction size, eluent, R_f_ value. Melting point: melting point apparatus Stuart Scientific^®^ SMP 3 (Bibby Sterilin Ltd., Staffsordshire, UK), uncorrected. IR: IR spectrophotometer FT-ATR-IR (Jasco^®^, Cremella (Lc), Italy). ^1^H NMR (400 MHz): Unity Mercury Plus 400 spectrometer (Varian^®^, Palo Alto, CA, US), AV400 (Bruker^®,^ Bremen, Germany), JEOL JNM-ECA-400 (Freising, Germany). ^13^C NMR (100 MHz): Unity Mercury plus 400 spectrometer (Varian^®®^) JEOL JNM-ECA-400; δ in ppm relative to tetramethylsilane; coupling constants are given with 0.5 Hz resolution, the assignments of ^13^C and ^1^H NMR signals were supported by 2D NMR techniques; MS: APCI = atmospheric pressure chemical ionization, EI = electron impact, ESI = electro-spray ionization: MicroTof (Bruker Daltronics, Bremen, Germany), calibration with sodium formate clusters before measurement. All solvents were of analytical grade quality and demineralized water was used. HPLC solvents were of gradient grade quality, and ultrapure water was used. All HPLC eluents were degassed by sonication prior to use. Thin-layer chromatography was conducted with silica gel F_254_ on aluminum plates in a saturated chamber at room temperature. The spots were visualized using UV light (254 nm) or reagents such as cerium molybdate dipping bath with additional heating using a standard heat gun. The retention factor values strongly depend on the temperature, the chamber saturation, and exact ratio of components of the eluent (highly volatile); the given retention factor values represent just approximate values. Flash column chromatography was conducted with silica gel 600 (40–63 μm, Macherey-Nagel). X-ray crystal structures: Equipment: Bruker APEX II CCD diffractometer (Bruker, Bremen, Germany): four circle diffractometer, Cu X-ray tube, graphite monochromator, APEX II CCD surface detector, Oxford Cryosystem 700 series (Oxford, UK) (N_2_ flow: 100–300 K).

#### 4.1.2. X-ray Diffraction Measurements

Data sets for compounds **19** and **21** were collected with a Nonius Kappa CCD rotating anode diffractometer. Programs used: data collection, COLLECT^xx^ (R. W. W. Hooft, Bruker AXS, 2008, Delft, The Netherlands); data reduction Denzo-SMN [19]; absorption correction, Denzo [20]; structure solution SHELXS-97 [21]; structure refinement SHELXL-97 [22]. Last-step refinement was done with the new software APEX3 V2019.1–0 (Bruker AXS (2019) APEX3 Version 2019.1–0, Bruker AXS Inc., Madison, WI, USA); structure refinement, SHELXL-2015 [23]; graphics, XP (Version 5.1, Bruker AXS Inc., Madison, WI, USA, 1998). *R*-values are given for observed reflections, and *w*R^2^ values are given for all reflections.

X-ray crystal structure analysis of **19**: a colorless, plate-like specimen of C_6_H_3_ClN_4_O_2_, approximate dimensions 0.060 mm × 0.200 mm × 0.260 mm, was used for the X-ray crystallographic analysis. The X-ray intensity data were measured on a rotating anode Nonius FR591 system equipped with a Mo rotating anode Mo rotating anode (Mo Kα, λ = 0.71073 Å) and a Montel mirror monochromator. The integration of the data using an orthorhombic unit cell yielded a total of 3297 reflections to a maximum θ angle of 28.13° (0.75 Å resolution), of which 1804 were independent (average redundancy 1.828, completeness = 99.1%, R_int_ = 1.94%, R_sig_ = 2.27%) and 1633 (90.52%) were greater than 2σ(F^2^). The final cell constants of a = 11.3385(3) Å, b = 6.5407(2) Å, c = 20.0740(7) Å, volume = 1488.72(8) Å^3^, are based upon the refinement of the XYZ-centroids of reflections above 20 σ(I). Data were corrected for absorption effects using the multi-scan method (SADABS). The calculated minimum and maximum transmission coefficients (based on crystal size) are 0.8850 and 0.9720. The structure was solved and refined using the Bruker SHELXTL Software Package, using the space group *Pbca*, with Z = 8 for the formula unit, C_6_H_3_ClN_4_O_2_. The final anisotropic full-matrix least-squares refinement on F^2^ with 122 variables converged at R1 = 3.16% for the observed data and wR2 = 8.40% for all data. The goodness-of-fit was 1.101. The largest peak in the final difference electron density synthesis was 0.293 e^−^/Å^3^ and the largest hole was −0.263 e^−^/Å^3^ with an RMS deviation of 0.050 e^−^/Å^3^. On the basis of the final model, the calculated density was 1.772 g/cm^3^ and F(000), 800 e^−^. The hydrogen at N2 atom was refined freely.

X-ray crystal structure analysis of **21**: A pale yellow, prism-like specimen of C_27_H_24_ClN_5_O_4_S, approximate dimensions 0.070 mm × 0.160 mm × 0.200 mm, was used for the X-ray crystallographic analysis. The X-ray intensity data were measured on a rotating anode Nonius FR591 system equipped with a Mo rotating anode (Mo Kα, λ = 0.71073 Å) and a Montel mirror monochromator. The integration of the data using a monoclinic unit cell yielded a total of 9321 reflections to a maximum θ angle of 26.73° (0.79 Å resolution), of which 5490 were independent (average redundancy 1.698, completeness = 98.2%, R_int_ = 2.98%, R_sig_ = 3.82%) and 4655 (84.79%) were greater than 2σ(F^2^). The final cell constants of a = 13.4033(2) Å, b = 9.4786(2) Å, c = 20.9689(4) Å, β = 98.6230(10)°, volume = 2633.87(8) Å^3^ are based upon the refinement of the XYZ-centroids of reflections above 20 σ(I). Data were corrected for absorption effects using the multi-scan method (SADABS). The calculated minimum and maximum transmission coefficients (based on crystal size) are 0.9480 and 0.9810. The structure was solved and refined using the Bruker SHELXTL Software Package, using the space group *P*2_1_/*n*, with Z = 4 for the formula unit, C_27_H_24_ClN_5_O_4_S. The final anisotropic full-matrix least-squares refinement on F^2^ with 348 variables converged at R1 = 4.71% for the observed data and wR2 = 10.64% for all data. The goodness-of-fit was 1.076. The largest peak in the final difference electron density synthesis was 0.214 e^−^/Å^3^ and the largest hole was −0.339 e^−^/Å^3^ with an RMS deviation of 0.047 e^−^/Å^3^. On the basis of the final model, the calculated density was 1.387 g/cm^3^ and F(000), 1144 e^−^. The hydrogen atom at N1 was refined freely.

CCDC-2157452 (compound **19**) and -2157453 (compound **21**) contain the supplementary crystallographic data for this paper. These data can be obtained free of charge from The Cambridge Crystallographic Data Centre via www.ccdc.cam.ac.uk/data_request/cif.

#### 4.1.3. HPLC Purity Measurements

Equipment: UV-detector: UltiMate 3000 variable Wavelength Detector; autosampler: UltiMate 3000; pump: Ultimate 3000; degasser: Ultimate 3000: data acquisition: Chromeleon Client 8.0.0 (Dionex Corpor., Sunnyvale, CA, USA). Method: column: guard column: Zorbax SB-Aq 12.5 × 4.6 mm catridge, column: Zorbax SB-Aq StableBond analytical 150 × 4.6 mm, flow rate: 1.00 mL/min; injection volume: 5.0 µL; detection at λ = 210 nm; Method A: solvents: A: Tetrabutylammonium phosphate buffer (5 mM) in H_2_O, B: CH_3_CN, gradient elution: (A%): 0–20 100 to 90%, 20–30 min: gradient from 90% to 100%. Method B: solvents: A: Tetrabutylammonium phosphate buffer (5 mM) in H_2_O, B: CH_3_CN, gradient elution: (A%): 0–20 min 80 to 20%, 20–30 min: gradient from 20% to 80%. Method C: solvents: A: Tetrabutylammonium phosphate buffer (5 mM) in H_2_O, B: CH_3_CN, gradient elution: (A%): 40–100%, 20–30 min: gradient from 100% to 40%. Method D: HPLC method for determination of the product purity: Merck Hitachi Equipment; UV detector: L-7400; autosampler: L-7200; pump: L-7100; degasser: L-7614; Method: column: LiChrospher^®®^ 60 RP-select B (5 µm), 250 × 4 mm^2^ column; flow rate: 1.00 mL/min; injection volume: 5.0 µL; detection at λ = 210 nm; solvents: A: water with 0.05% (*v*/*v*) trifluoroacetic acid; B: acetonitrile with 0.05% (*v*/*v*) trifluoroacetic acid: gradient elution: (A%): 0–4 min: 90%, 4–29 min: gradient from 90% to 0%, 29–31 min: 0%, 31–31.5 min: gradient from 0% to 90%, 31.5–40 min: 90%.

#### 4.1.4. Data Analysis

NMR spectra were processed with MestReNova 12.0 (MestreLab Research, Santiago de Compostela, Spain).

### 4.2. Adenosine Receptor Binding Studies

#### 4.2.1. Cell Culture and Membrane Preparation

Chinese hamster ovary (CHO) cells stably expressing the human adenosine A_1_ receptor (CHOhA1R) were kindly provided by Prof. S. J. Hill and CHO cells stably expressing the human adenosine A_3_ receptor (CHOhA_3_R) were a gift from Dr. K.-N. Klotz (University of Würzburg, Germany). Chinese hamster ovary cells stably expressing the human A_1_-receptor (CHOhA_1_R) or the human A_3_-receptor (CHOhA_3_R) were grown in Dulbecco’s Modified Eagle’s Medium (DMEM) and Ham’s F12 medium (1:1) supplemented with 10% (*v*/*v*) newborn calf serum, 50 µg/mL streptomycin, 50 IU/mL penicillin, and 200 µg/mL G418 at 37 °C and 5% CO_2_. CHOhA_1_R cells were subcultured twice a week at a ratio of 1:20 on 10 cm Ø plates and 15 cm Ø plates. CHOhA_3_R cells were subcultured twice a week at a ratio of 1:8 on 10 cm Ø plates and 15 cm Ø plates.

Human embryonic kidney 293 cells stably expressing the human adenosine A_2A_ receptor (HEK293hA_2A_R) were kindly provided by Dr. J. Wang (Biogen/IDEC, Cambridge, MA, USA), CHO-spap cells stably expressing the wild-type (WT) hA_2B_ receptor (CHO-spap-hA_2B_R) were kindly provided by S. J. Dowell (GlaxoSmithKline, Brentfort, UK). Human embryonic kidney cells from the cell line 293 stably expressing the human A_2A_-receptor (HEK_293_hA_2A_R) were grown in culture medium consisting of DMEM supplemented with 10% (*v*/*v*) newborn calf serum, 50 µg/mL streptomycin, 50 IU/mL penicillin, and 500 µg/mL G418 at 37 °C and 7% CO_2_. Cells were subcultured twice a week at a ratio of 1:8 on 10 cm Ø plates and 15 cm Ø plates.

Chinese hamster ovary cells stably expressing the human A_2A_-receptor and a reporter gene, the secreted placental alkaline phosphatase, (CHO-spap-hA_2B_R) were grown in DMEM and Ham’s F12 medium (1:1) supplemented with 10% (*v*/*v*) newborn calf serum, 100 µg/mL streptomycin, 100 IU/mL penicillin, 1 mg/mL G418, and 0.4 mg/mL hygromycin at 37 °C and 5% CO_2_. Cells were subcultured at a ratio of 1:20 twice a week.

All cells were grown to 80–90% confluency and detached from plates by scraping them into 5 mL phosphate-buffered saline. Detached cells were collected and centrifuged at 200 g for 5 min. Pellets derived from 100 15 cm Ø plates were pooled and resuspended in 70 mL of ice-cold 50 mM tris(hydroxymethyl)aminomethane (Tris)-HCl buffer, pH 7.4. A Heidolph Diax 900 homogenizer was used to homogenize the cell suspension. Membranes and the cytosolic fraction were separated by centrifugation at 100,000 g in a Beckman Optima LE-80 K ultracentrifuge (Beckman Coulter, Fullerton, CA, USA) at 4 °C for 20 min. The pellet was resuspended in 35 mL of the Tris-HCl buffer, and the homogenization and centrifugation steps were repeated. Tris-HCl buffer (25 mL) was used to resuspend the pellet, and adenosine deaminase (ADA) was added (0.8 U/mL) to break down endogenous adenosine. Membranes were stored in 250 µL and 500 µL aliquots at −80 °C. Total protein concentrations were measured using the bicinchoninic acid (BCA) method.

#### 4.2.2. Radioligand Displacement Assay

A_1_ Receptor: Membrane aliquots containing 5 µg (CHOhA_1_R) protein were incubated in a total volume of 100 µL assay buffer (50 mM Tris-HCl, pH 7.4) at 25 °C for 1 h. Radioligand displacement experiments were performed using six concentrations of competing ligand in the presence of 1.6 nM [^3^H]8-cyclopentyl-1,3-dipropylxanthine ([^3^H]DPCPX). At these concentrations, total radioligand binding did not exceed 10% of that added to prevent ligand depletion. Nonspecific binding was determined in the presence of 100 µM *N*^6^-cyclopentyladenosine (CPA). Incubations were terminated by rapid vacuum filtration to separate the bound and free radioligand through prewetted 96-well GF/B filter plates using a PerkinElmer Filtermate-harvester (Perkin Elmer, Groningen, the Netherlands). Filters were subsequently washed 12 times with ice-cold 50 mM Tris-HCl, pH 7.4.

A_2A_ Receptor: Membrane aliquots containing 30 µg (HEK_293_hA_2A_R) total protein were incubated in a total volume of 100 µL assay buffer (50 mM Tris-HCl, pH 7.4) at 25 °C for 1 h. Radioligand displacement experiments were performed using six concentrations of competing ligand in the presence of 5.5 nM [^3^H]4-[2-[7-amino-2-(2-furyl)-1,2,4-triazolo[1,5-a][1,3,5]triazin-5-yl-amino]ethyl]phenol ([^3^H]ZM241385). At these concentrations, total radioligand binding did not exceed 10% of that added to prevent ligand depletion. Nonspecific binding was determined in the presence of 100 µM adenosine-5-*N*-ethyluronamide (NECA). Incubations were terminated by rapid vacuum filtration to separate the bound and free radioligand through prewetted 96-well GF/B filter plates using a PerkinElmer Filtermate-harvester (Perkin Elmer, Groningen, The Netherlands). Filters were subsequently washed 12 times with ice-cold 50 mM Tris-HCl, pH 7.4.

A_2B_ Receptor: Membrane aliquots containing 30 µg (CHO-spap-hA_2B_R) total protein were incubated in a total volume of 100 µL assay buffer (0.1% CHAPS in 50 mM TrisHCl, pH 7.4) at 25 °C for 2 h. Radioligand displacement experiments were performed using six concentrations of competing ligand in the presence of 1.5 nM [^3^H]8-[4-[4-(4-chlorophenyl)piperazide-1-sulfonyl)phenyl]]-1-propylxanthine ([^3^H]PSB-603). At these concentrations, total radioligand binding did not exceed 10% of that added to prevent ligand depletion. Nonspecific binding was determined in the presence of 10 µM ZM241385. Incubations were terminated by rapid vacuum filtration to separate the bound and free radioligand through prewetted 96-well GF/B filter plates using a PerkinElmer Filtermate-harvester (Perkin Elmer, Groningen, the Netherlands). Filters were subsequently washed 12 times with ice-cold 0.1% BSA in 50 mM Tris-HCl, pH 7.4.

A_3_ Receptor: Membrane aliquots containing 15 µg (CHOhA_3_R) total protein were incubated in a total volume of 100 µL assay buffer (50 mM Tris-HCl, pH 8.0, supplemented with 10 mM MgCl_2_, 1 mM EDTA and 0.01% (*w*/*v*) CHAPS) at 25 °C for 2 h. Radioligand displacement experiments were performed using six concentrations of competing ligand in the presence of 10 nM [^3^H]8-ethyl-4-methyl-2-phenyl-(8*R*)-4,5,7,8-tetrahydro-1*H*-imidazo[2.1-i]purin-5-one ([^3^H]PSB11). At these concentrations, total radioligand binding did not exceed 10% of that added to prevent ligand depletion. Nonspecific binding was determined in the presence of 100 µM NECA. Incubations were terminated by rapid vacuum filtration to separate the bound and free radioligand through prewetted 96-well GF/B filter plates using a PerkinElmer Filtermate-harvester (Perkin Elmer, Groningen, The Netherlands). Filters were subsequently washed 12 times with ice-cold 50 mM Tris-HCl supplemented with 10 mM MgCl_2_, and 1 mM EDTA, pH 8.0 for CHOhA_3_R.

The plates of all four adenosine receptor assays were dried at 55 °C after which MicroscintTM-20-cocktail was added (Perkin Elmer, Groningen, The Netherlands). After 3 h the filter-bound radioactivity was determined by scintillation spectrometry using a 2450 MicroBeta Microplate Counter (Perkin Elmer, Groningen, The Netherlands).

#### 4.2.3. Data Analysis

All experimental data were analyzed using the non-linear regression curve fitting program GraphPad Prism 7.0 (GraphPad Software Inc., San Diego, CA, USA). IC_50_ values obtained from competition displacement binding data were converted into *K_i_* values using the Cheng–Prusoff equation. The *K*_D_ value (1.6 nM) of [^3^H]DPCPX at CHOhA_1_R membranes was taken from Kourounakis et al. [24]. The *K*_D_ value (1.0 nM) of [^3^H]ZM241385 at hA_2A_R membranes, the *K*_D_ value (1.71 nM) of [^3^H]PSB603 at CHspap-hA_2B_R membranes, and the *K*_D_ value (17.3 nM) of [^3^H]PSB11 at CHOhA_3_R membranes were taken from in-house determinations.

### 4.3. P2Y_1_ Receptor Studies

#### 4.3.1. Cell Culture

Human astrocytoma cell lines expressing human P2Y_1_ receptor (1321N1-HA-P2Y1, Kerafast) were maintained in high-glucose Dulbecco’s modified Eagle’s medium (DMEM) supplemented with 10% fetal calf serum and 1% Penicillin/Streptomycin (10.000 units penicillin and 10 mg streptomycin per mL in 0.9% NaCl, Sigma Aldrich) in tissue culture 75 cm^2^ flasks and subcultured every 2–4 days (1:3, 1:10) once confluent.

#### 4.3.2. Ca^2+^-Flux Assay

Fluo-4 Direct was prepared according to the manufacturer’s instructions. Human Astrocytoma cell line stably expressing the P2Y_1_ receptor (1321N1-HA-P2Y_1_, Kerafast) was seeded into black clear-bottom Nunc 96 well plates (Thermo Fisher Scientific) at 3.0–4.0 × 10^4^ cells/well and incubated for 48 h at 37 °C and 5% CO_2_ until cells reach confluence level of at least 85–90%. The medium was removed, and the cells were washed using 100 µL HBSS containing 20 mM HEPES. Loading cells with the fluorescent Ca^2+^ indicator Fluo-4 were performed at 37 °C for 40 min and an additional 20 min at room temperature, followed by 30 min of incubation in the presence (antagonist mode) of five different concentrations of antagonists (10^−4^ to 10^−8^ M) or absence of antagonists (agonist mode, mock solution 50 µL HBSS containing 20 mM HEPES and 2% DMSO). Followed by the application of ADP (concentration of determined EC_50_-value, antagonist mode) or different concentrations of potential agonists (10^−4^ to 10^−8^ M, agonist mode) and the changes of intracellular Ca^2+^ concentrations were monitored over 200 s using a FlexStation^®®^ 3 Multi-Mode Microplate Reader (Molecular Devices, San Jose, CA, USA, SoftMax7 Pro, excitation: 494 nm, emission: 516 nm). The concentration-dependent increase or decrease of Ca^2+^-flux was plotted against the logarithmic concentrations of compounds.

#### 4.3.3. Data Analysis

The activation or inhibition curves of three independent measurements, each done in duplicates, were fitted to Hill equation using GraphPad Prism software version 9.3.1 (GraphPad Software Inc. San Diego, CA, USA).

### 4.4. Synthetic Procedures

*(1R,2R,3S,4S,5S)-1-{[(tert-Butyldiphenylsilyl)oxy]methyl-2,3-O-isopropylidenebicyclo [3.1.0]hexan-2,3,4-triol* (**4**). The procedure was modified according to reference [16]. The (1*S*,2*S*,3*R*)-4-{[(*tert*-Butyldiphenylsilyl)oxy]methyl}-2,3-*O*-isopropylidene-4-cyclopenten-1,2,3-triol (1.01 g, 2.37 mmol) was dissolved in dry CH_2_Cl_2_ (13 mL) under a nitrogen atmosphere. The reaction was cooled down to −18 °C with an ice/salt bath. Diethylzinc (1 mol/L in hexane, 2.60 mL, 2.60 mmol, 1.1 eq.) was added dropwise, and the mixture was stirred for 15 min. Diidomethane (0.22 mL, 2.73 mmol, 1.15 eq.) in dry CH_2_Cl_2_ (1.6 mL) was also added dropwise and the reaction was stirred for another 15 min. Both steps were repeated a second time. Then diethylzinc (1 mol/L in hexane, 2.60 mL, 2.60 mmol, 1.1 eq.) was added for the third time. After stirring for 15 min at −18 °C, the reaction was allowed to warm to rt and stirred overnight. The reaction was quenched with saturated NH_4_Cl-solution and was extracted five times with CH_2_Cl_2_. The organic phase was dried over anh. Na_2_SO_4_ and concentrated in vacuo. The residue was purified by fc (cyclohexane:ethyl acetate = 7:1, Ø = 5 cm, l = 22 cm, V = 30 mL) to afford the product **4** as a colorless oil (R_f_ = 0.20, cyclohexane: ethyl acetate = 5:1), yield 0.90 g (86%). C_26_H_34_O_4_Si (438.64 g/mol). Purity (HPLC: method B): > 99% (t_R_ = 18.94 min). Exact mass (APCI): *m*/*z* calculated for C_23_H_27_O_2_Si [M-OH, -CO(CH_3_)_2_]^+^ 363.1775, found 363.1777. ^1^H-NMR (600 MHz, CDCl_3_) δ (ppm) = 7.66–7.60 (m, 4H, 2, 6-C*H_Ph_*), 7.46–7.34 (m, 6H, 3, 4, 5C*H_Ph_*), 5.00 (dd, *J* = 6.9, 1.2 Hz, 1H, 2-C*H*), 4.54 (td, *J* = 6.9, 0.8 Hz, 1H, 3-C*H*), 4.45 (dt, *J* = 9.6, 6.1 Hz, 1H, 4C*H*), 4.12 (q, *J* = 7.2 Hz, 0.2H, C*H*_2_, solvent: ethyl acetate), 4.07 (d, *J* = 11.0 Hz, 1H, OC*H*H), 3.29 (d, *J* = 11.0 Hz, 1H, OCH*H*), 2.33 (d, *J* = 9.7 Hz, 1H, O*H*), 2.04 (s, 0.3H, OC*H*_3_, solvent: ethyl acetate), 1.61 (dt, *J* = 9.3, 4.9 Hz, 1H, 5C*H*), 1.54 (s, 3H, C(C*H*_3_)_2_), 1.31 (s, 3H, C(C*H*_3_)_2_), 1.26 (t, *J* = 7.1 Hz, 0.5H, CH_2_C*H*_3_, solvent: ethyl acetate), 1.09 (t, *J* = 5.0 Hz, 1H, 6-C*H*H), 1.05 (s, 9H, C(C*H*_3_)_3_), 0.54 (ddt, *J* = 8.8, 5.3, 1.1 Hz, 1H, 6-CH*H*). ^13^C-NMR (151 MHz, CDCl_3_) δ (ppm) = 135.7 (4C, *C*-2, 6*_Ph_*), 133.8, 133.7 (2C, *C*-1*_Ph_*), 129.9 (2C, *C*-4*_Ph_*), 127.8 (4C, *C*-3, 5*_Ph_*), 113.0 (1C, *C*(CH_3_)_2_), 81.3 (1C, *C*2), 79.9 (1C, *C*3), 71.2 (1C, *C*4), 65.4 (1C, O*C*H_2_), 35.7 (1C, *C*-1), 33.0 (1C, *C*-5), 27.0 (3C, C(*C*H_3_)_3_), 26.3 (1C, C(*C*H_3_)_2_), 24.8 (1C, C(*C*H_3_)_2_), 19.4 (1C, *C*(CH_3_)_3_), 10.5 (1C, *C*-6). FT-IR (neat) *ṽ* (cm^−1^) = 2932, 2859 (C-H_aliphat_.), 1470 (C = C_aromat_.), 1107, 1080, 1042 (CO), 741, 702 (CH_aromat_., out of plane).

*7-Chloro-5-nitro-3H-imidazo[4,5-b]pyridine* (**7**). An amount of 6-chloro-1-deazapurine (1.01 g, 6.5 mmol) and di-*tert*-butyl dicarbonate (3.02 g, 16.6 mmol, 2.5 eq.) were suspended in CH_2_Cl_2_ (20 mL). DMAP (0.04 g, 0.3 mmol, 0.1 eq.) was added and the mixture was stirred for 1.5 h. The reaction was quenched with silica gel and filtered through a pad of Celite^®®^. The mixture was concentrated in vacuo and the residue was redissolved in CH_2_Cl_2_ (20 mL). Tetrabutylammonium nitrate (3.07 g, 10.1 mmol, 1.5 eq.) was added and the mixture cooled to 0 °C with an ice bath. Trifluoroacetic anhydride (1.8 mL, 10.1 mmol, 1.5 eq.) was added dropwise and the reaction stirred for 2.5 h at rt. The solvent was evaporated, and the residue was dissolved in CH_3_OH (40 mL). The solution was refluxed overnight. The mixture was concentrated until the product was precipitating but was still properly suspended. After cooling the suspension in the fridge for 1 h, the solid was filtered off, washed with ice cold CH_3_OH, and dried in vacuo to afford the product **77** as a beige solid (R_f_ = 0.43, CH_2_Cl_2_: CH_3_OH = 9:1), yield 1.02 g (78%). C_6_H_3_ClN_4_O_2_ (198.57 g/mol). Melting point: 295.9 °C. Purity (HPLC: method B): > 99% (t_R_ = 5.67 min). Exact mass (APCI): *m*/*z* calculated for C_6_H_4_ClN_4_O_2_ [M + H]^+^ 199.0007, found 199.0017. ^1^H-NMR (600 MHz, DMSO-*d*_6_) δ (ppm) = 8.90 (s, 1H, 2-C*H*), 8.36 (s, 1H, 6-C*H*). ^13^C-NMR (151 MHz, DMSO-*d*_6_) δ (ppm) = 151.5 (1C, *C*-5), 149.6 (1C, *C*-2), 112.8 (1C, *C*-6); C-3a, C-7 and C-7a were not visible. FT-IR (neat) *ṽ* (cm^−1^) = 3098, 3013 (v C-H_aromat_.), 2743, 2677, 2612, 2554 (N-H), 1543, 1501 (C = C_aromat_.), 833, 880 (C-H_aromat_., out of plane).

*(1R,2R,3S,4R,5S)-4-(5-Amino-7-chloro-3H-imidazo[4,5-b]pyridin-3-yl)-1-{[(tert-butyldiphenylsilyl)oxy]methyl}-2,3-O-isopropylidenebicyclo[3.1.0]hexane-2,3-diol* (**10**). The deazapurine **7** (0.29g, 1.48 mmol, 1.3 eq.) and triphenylphospane (0.61 g, 2.31 mmol, 2.1 eq.) were dissolved in THF (10 mL) under nitrogen atmosphere. DIAD (0.42 mL, 2.14 mmol, 1.9 eq.) was added dropwise at 0 °C. The mixture was stirred for 15 min at rt. A solution of the alcohol **4** (0.49 g, 1.11 mmol) in THF (13 mL) was added and the mixture was stirred at 70 °C for 1 h in the microwave at a power of 200 W. DIAD (0.42 mL, 2.14 mmol, 1.9 eq.) was added and the mixture was stirred again at 70 °C for 1 h in the microwave at a power of 200 W. DIAD (0.42 mL, 2.14 mmol, 1.9 eq.) was added and the mixture was stirred at 70 °C for 1 h in the microwave at a power of 200 W for the third time. The solvent was evaporated and the residue was purified by fc (cyclohexane: ethyl acetate = 5:1, Ø = 5 cm, l = 20 cm, V = 30 mL), but the intermediate was still heavily contaminated with DIAD and was directly dissolved in CH_3_OH (40 mL). Na_2_S_2_O_4_ (1.51 g, 8.64 mmol, 7.8 eq.) and 10 mL H_2_O were added. The mixture was stirred for 3d at rt. The solvent was evaporated and the residue was purified by fc (cyclohexane: ethyl acetate = 5:1 ⭢ 4:1 ⭢ 2:1, Ø = 5 cm, l = 20 cm, V = 30 mL) to afford the product **10** as colorless solid (R_f_ = 0.42, cyclohexane: ethyl acetate = 1:1), yield 0.138 g (21%). C_32_H_37_ClN_4_O_3_Si (589.21 g/mol). Melting point: 88.9 °C. Purity (HPLC: method B): > 99% (t_R_ = 21.36 min).

Exact mass (LC-MS-ESI): *m*/*z* calculated for C_32_H_38_ClN_4_O_3_Si [M + H]^+^ 589.2396, found 589.2401. ^1^H-NMR (600 MHz, DMSO-*d*_6_) δ (ppm) = 8.15 (s, 1H, 2-CH_imidazopyridine_), 7.63–7.58 (m, 4H, 2, 6CH_Ph_), 7.47–7.43 (m, 2H, 4-CH_Ph_), 7.41–7.37 (m, 4H, 3, 5-CH_Ph_), 6.52 (s, 1H, 6-CH_imidazopyridine_), 6.26 (s, 2H, NH_2_), 5.33 (dd, J = 7.1, 1.3 Hz, 1H, 2-CH), 4.89 (s, 1H, 4-CH), 4.65 (dd, J = 7.1, 1.5 Hz, 1H, 3-CH), 4.10 (d, J = 10.8 Hz, 1H, OCHH), 3.64 (d, J = 10.8 Hz, 1H, OCHH), 1.66 (ddd, J = 9.2, 4.4, 1.5 Hz, 1H, 5CH), 1.44 (s, 3H, C(CH_3_)_2_), 1.19 (s, 3H, C(CH_3_)_2_), 1.02 (s, 9H, C(CH_3_)_3_), 0.97 (t, J = 4.7 Hz, 1H, 6CHH), 0.88 (ddd, J = 9.1, 5.0, 1.5 Hz, 1H, 6-CHH); the ^1^HNMR spectrum displayed small impurities in the range of about 5%. ^13^C-NMR (151 MHz, DMSO-d_6_) δ (ppm) = 157.4 (1C, C-5_imidazopyridine_), 145.9 (1C, C3a_imidazopyridine_), 138.3 (1C, C-2_imidazopyridine_), 135.1 (4C, C-2, 6_Ph_), 134.1 (1C, C7_imidazopyridine_), 132.7 (2C, C-1_Ph_), 129.9 (2C, C4_Ph_), 127.9 (4C, C3, 5_Ph_), 124.5 (1C, C7a_imidazopyridine_), 111.2 (1C, C(CH_3_)_2_), 104.1 (1C, C6_imidazopyridine_), 88.0 (1C, C3), 80.7 (1C, C2), 64.7 (1C, OCH_2_), 57.8 (1C, C-4), 38.1 (1C, C1), 29.9 (1C, C-5), 26.7 (3C, C(CH_3_)_3_), 25.9 (1C, C(CH_3_)_2_), 24.3 (1C, C(CH_3_)_2_), 18.8 (1C, C(CH_3_)_3_), 11.9 (1C, C-6); the ^13^C-NMR spectrum displayed small impurities in the range of about 5%. FT-IR (neat) *ṽ* (cm^−1^) = 3333 (N-H), 2932 (C-H_aliphat_.), 1601, 1570 (C = C_aromat_.), 1107, 1061, 1038 (C-O), 741, 702 (CH_aromat_., out of plane).

*(1R,2R,3S,4R,5S)-4-(5-Amino-7-chloro-3H-imidazo[4,5-b]pyridin-3-yl)-1-(hydroxymethyl)bicyclo[3.1.0]hexane-2,3-diol* (**11**). Compound **10** (0.068 g, 0.12 mmol) was dissolved in CH_3_OH (1.6 mL), trifluoroacetic acid (0.20 mL) and H_2_O (0.20 mL) were added. The mixture was heated to 70 °C for 1 d. The solvent was evaporated and the residue was purified by semi-preparative HPLC (method B) to afford the alcohol **11** as a colorless solid (R_f_ = 0.2, CH_2_Cl_2_: CH_3_OH = 9:1), yield 0.012 g (34%). C_13_H_15_ClN_4_O_3_ (310.74 g/mol). Purity (HPLC: method D): 99% (t_R_ = 6.97 min). Exact mass (LC-MS-ESI): *m*/*z* calculated for C_13_H_16_ClN_4_O_3_ [M + H]^+^ 311.0905, found 311.0908. ^1^H-NMR (600 MHz, DMSO-d_6_) δ (ppm) = 8.32 (s, 1H, 2-CH_imidazopyridine_), 6.49 (s, 1H, 6-CH_imidazopyridine_), 6.23 (s, 2H, NH_2_), 5.02 (s, 2H, CH_2_OH, 3-OH), 4.71 (s, 1H, 4-CH), 4.57 (d, J = 6.3 Hz, 2H, 2-CH, 2-OH), 4.08 (d, J = 11.3 Hz, 1H, OCHH), 3.64 (d, J = 6.3 Hz, 1H, 3-CH), 3.12 (d, J = 11.3 Hz, 1H, OCHH), 1.41 (ddd, J = 8.7, 3.9, 1.4 Hz, 1H, 5-CH), 1.32 (t, J = 4.3 Hz, 1H, 6-CHH), 0.78 (ddd, J = 8.6, 4.7, 1.3 Hz, 1H, 6-CHH). ^13^C-NMR (151 MHz, DMSO-*d*_6_) δ (ppm) = 157.2 (1C, C-5_imidazopyridine_), 146.1 (1C, C-3a_imidazopyridine_), 138.5 (1C, C-2_imidazopyridine_), 133.9 (1C, C-7_imidazopyridine_), 124.4 (1C, C-7a_imidazopyridine_), 104.0 (1C, C-6_imidazopyridine_), 76.1 (1C, C-3), 70.2 (1C, C-2), 62.2 (1C, OCH_2_), 60.2 (1C, C-4), 36.3 (1C, C-1), 23.4 (1C, C-5), 11.1 (1C, C-6). FT-IR (neat) *ṽ* (cm^−1^) = 3321, 3206 (O-H), 2920 (C-H_aliphat_.), 1601, 1574 (C = C_aromat_.), 1061, 1003 (C-O).

*(1R,2R,3S,4R,5S)-1-{[(tert-Butyldiphenylsilyl)oxy]methyl}-4-(5,7-dichloro-3H-imidazo[4,5-b]pyridin-3-yl)-2,3-O-isopropylidenebicyclo[3.1.0]hexane-2,3-diol* (**9**). An amount of 2,6-dichloro-1-deazapurine (**6**, 0.16 g, 0.86 mmol, 1.2 eq.) and triphenylphospane (0.36 g, 1.37 mmol, 1.9 eq.) were dissolved in THF (7 mL) under nitrogen atmosphere. DIAD (0.27 mL, 1.38 mmol, 1.9 eq.) was added dropwise at 0 °C. The mixture was stirred for 30 min at rt. A solution of the alcohol **4** (0.32 g, 0.73 mmol) in THF (7 mL) was added and the mixture was stirred at 70 °C for 1 h in the microwave at a power of 200 W. DIAD (0.27 mL, 1.38 mmol, 1.9 eq.) was added and the mixture was stirred at 70 °C for 1 h in the microwave at a power of 200 W again. DIAD (0.27 mL, 1.38 mmol, 1.9 eq.) was added and the mixture was stirred at 70 °C for 1 h in the microwave at a power of 200 W for the third time. The solvent was evaporated and the residue was purified by fc (cyclohexane: ethyl acetate = 7:1, Ø = 5 cm, l = 20 cm, V = 30 mL) to afford the product **87** as a colorless solid (R_f_ = 0.26, cyclohexane: ethyl acetate = 5:1), yield 0.40 g (89%). C_32_H_35_Cl_2_N_3_O_3_Si (608.64 g/mol). Melting point: 75.7 °C. Purity (HPLC: method C): 98% (t_R_ = 17.28 min). Exact mass (LC-MS-ESI): *m*/*z* calculated for C_32_H_36_Cl_2_N_3_O_3_Si [M + H]^+^ 608.1898, found 608.1899. ^1^H-NMR (600 MHz, DMSO-*d*_6_) δ (ppm) = 8.67 (s, 1H, 2-C*H_imidazopyridine_*), 8.16 (s, 1H, 6C*H_imidazopyridine_*), 7.59 (ddt, *J* = 10.6, 6.8, 1.4 Hz, 4H, 2, 6C*H_Ph_*), 7.46–7.40 (m, 2H, 4C*H_Ph_*), 7.40–7.37 (m, 2H, 3, 5-C*H_Ph_*), 7.36–7.32 (m, 2H, 3, 5-C*H_Ph_*), 5.23 (dd, *J* = 7.1, 1.3 Hz, 1H, 2C*H*), 5.04 (s, 1H, 4C*H*), 4.77 (dd, *J* = 7.1, 1.6 Hz, 1H, 3-C*H*), 4.02 (d, *J* = 10.6 Hz, 1H, OC*H*H), 3.90 (d, *J* = 10.6 Hz, 1H, OCH*H*), 1.72 (ddd, *J* = 9.2, 4.5, 1.6 Hz, 1H, 5C*H*), 1.46 (s, 3H, C(C*H*_3_)_2_), 1.18 (s, 3H, C(C*H*_3_)_2_), 1.01 (s, 10H, 6-C*H*H, C(C*H*_3_)_3_), 0.96 (ddd, *J* = 9.2, 5.1, 1.5 Hz, 1H, 6-CH*H*). ^13^C-NMR (151 MHz, DMSO-*d*_6_) δ (ppm) = 145.7 (1C, *C*-3a*_imidazopyridine_*), 144.9 (1C, *C*2*_imidazopyridine_*), 144.2 (1C, *C*-5*_imidazopyridine_*), 135.2 (1C, *C*-7*_imidazopyridine_*), 135.1 (4C, *C*2, 6*_Ph_*), 132.8 (2C, *C*-1*_Ph_*), 132.2 (1C, *C*7a*_imidazopyridine_*), 129.8 (2C, *C*4*_Ph_*), 127.8 (2C, *C*3, 5*_Ph_*), 127.8 (2C, *C*3, 5*_Ph_*), 118.1 (1C, *C*6*_imidazopyridine_*), 111.4 (1C, *C*(CH_3_)_2_), 87.9 (1C, *C*3), 81.6 (1C, *C*2), 64.5 (1C, O*C*H_2_), 59.3 (1C, *C*-4), 38.3 (1C, *C*1), 29.4 (1C, *C*-5), 26.7 (3C, C(*C*H_3_)_3_), 25.9 (1C, C(*C*H_3_)_2_), 24.2 (1C, C(*C*H_3_)_2_), 18.8 (1C, *C*(CH_3_)_3_), 12.0 (1C, *C*-6). FT-IR (neat) *ṽ* (cm^−1^) = 2978, 2932 (C-H_aliphat_.), 1589, 1562 (C = C_aromat_.), 1065, 1042 (CO), 741, 702 (CH_aromat_., out of plane).

*(1R,2R,3S,4R,5S)-4-(5,7-Dichloro-3H-imidazo[4,5-b]pyridin-3-yl)-1-(hydroxymethyl)bicyclo[3.1.0]hexane-2,3-diol* (**12**). Compound **9** (0.036 g, 0.06 mmol) was dissolved in CH_3_OH (1.1 mL), trifluoroacetic acid (0.14 mL) and H_2_O (0.14 mL) were added. The mixture was heated to 70 °C for 2 d. The solvent was evaporated and the residue was purified by fc (CH_3_CN: H_2_O = 5:95 ⭢ 100:0, 12 mL/min, Biotage^®®^ SNAP Ultra C18, 12 g, V = 20 mL) to afford the alcohol **12** as a colorless solid (R_f_ = 0.34, CH_2_Cl_2_: CH_3_OH = 9:1), yield 0.016 g (84%). C_13_H_13_Cl_2_N_3_O_3_ (330.17 g/mol). Purity (HPLC: method B): 98% (t_R_ = 5.95 min). Exact mass (APCI): *m*/*z* calculated for C_13_H_14_Cl_2_N_3_O_3_ [M + H]^+^ 330.0407, found 330.0406. ^1^H-NMR (600 MHz, CD_3_OD) δ (ppm) = 8.93 (s, 1H, 2-C*H_imidazopyridine_*), 7.47 (s, 1H, 6-C*H_imidazopyridin_*), 5.00 (s, 1H, 4-C*H*), 4.78 (dd, *J* = 6.6, 1.7 Hz, 1H, 2-C*H*), 4.28 (dd, *J* = 11.5, 0.9 Hz, 1H, OC*H*H), 3.91 (dt, *J* = 6.6, 1.3 Hz, 1H, 3-C*H*), 3.36 (d, *J* = 11.5 Hz, 1H, OCH*H*), 1.65 (ddd, *J* = 8.7, 3.9, 1.5 Hz, 1H, 5-C*H*), 1.58 (dd, *J* = 5.2, 3.9 Hz, 1H, 6-C*H*H), 0.78 (ddd, *J* = 8.7, 5.2, 1.8 Hz, 1H, 6-CH*H*). ^13^C-NMR (151 MHz, CD_3_OD) δ (ppm) = 147.2 (1C, *C*-3a*_imidazopyridine_*), 147.0 (1C, *C*-5*_imidazopyridine_*), 145.6 (1C, *C*-2*_imidazopyridine_*), 137.1 (1C, *C*-7*_imidazopyridine_*), 133.1 (1C, *C*-7a*_imidazopyridine_*), 119.9 (1C, *C*-6*_imidazopyridine_*), 77.5 (1C, *C*-3), 72.3 (1C, *C*-2), 64.3 (1C, O*C*H_2_), 63.6 (1C, *C*-4), 37.9 (1C, *C*-1), 24.5 (1C, *C*-5), 12.2 (1C, *C*-6). FT-IR (neat) *ṽ* (cm^−1^) = 3244 (O-H), 2978 (C-H_aliphat_.), 1593, 1562 (C = C_aromat_.), 1064, 1006 (C-O).

*(1R,2R,3S,4R,5S)-4-[7-(Benzylamino)-5-chloro-3H-imidazo[4,5-b]pyridin-3-yl]-1-(hydroxymethyl)bicyclo[3.1.0]hexane-2,3-diol* (**13**). Compound **9** (0.26 g, 0.43 mmol) was dissolved in *N*-methyl-2-pyrrolidone (NMP, 5.5 mL). Benzylamine (0.78 mL, 7.14 mmol, 17 eq.) and *N,N*-diisopropylethylamine (DIPEA, 0.52 mL, 3.06 mmol, 7 eq.) were added. The mixture was stirred at 200 °C for 1 h in the microwave at a power of 200 W. The solution was directly purified by fc (CH_3_CN: H_2_O = 30:70 ⭢ 100:0, 50 mL/min, Biotage^®®^ SNAP C18, 120 g, V = 20 mL) to afford the protected product as a colorless solid (R_f_ = 0.44, cyclohexane: ethyl acetate = 1:1), yield 0.25 g (86%). C_39_H_43_ClN_4_O_3_Si (679.33 g/mol). Melting point: 81.4 °C. Purity (HPLC: method C): 97% (t_R_ = 17.58 min). Exact mass (LC-MS-ESI): *m*/*z* calculated for C_39_H_44_ClN_4_O_3_Si [M + H]^+^ 678.2866, found 679.2898.^1^H-NMR (400 MHz, DMSO-*d*_6_) δ (ppm) = 8.25 (s, 1H, 2-C*H_imidazopyridine_*), 7.92 (t, *J* = 6.5 Hz, 1H, N*H*), 7.64–7.57 (m, 4H, 2, 6-C*H_Ph_*), 7.47–7.28 (m, 10H, 3, 4, 5-C*H_Ph_*, 2, 3, 5, 6-C*H_benzyl_*), 7.26–7.19 (m, 1H, 4-C*H_benzyl_*), 6.29 (s, 1H, 6-C*H_imidazopyridine_*), 5.26 (dd, *J* = 7.1, 1.4 Hz, 1H, 2-C*H*), 4.92 (s, 1H, 4-C*H*), 4.65 (dd, *J* = 7.2, 1.5 Hz, 1H, 3-C*H*), 4.62 (s, 2H, C*H_2 benzyl_*), 4.05 (d, *J* = 10.7 Hz, 1H, OC*H*H), 3.76 (d, *J* = 10.7 Hz, 1H, OCH*H*), 1.65 (ddd, *J* = 9.3, 4.4, 1.5 Hz, 1H, 5-C*H*), 1.44 (s, 3H, C(C*H*_3_)_2_), 1.18 (s, 3H, C(C*H*_3_)_2_), 1.01 (s, 10H, 6-C*H*H, C(C*H*_3_)_3_), 0.90 (ddd, *J* = 9.2, 5.1, 1.4 Hz, 1H, 6-CH*H*). ^13^C-NMR (101 MHz, DMSO-*d*_6_) δ (ppm) = 147.8 (1C, *C*-7*_imidazopyridine_*), 146.0 (1C, *C*-5*_imidazopyridine_*), 144.8 (1C, *C*-3a*_imidazopyridine_*), 139.2 (1C, *C*-1*_benzyl_*), 138.7 (1C, *C*-2*_imidazopyridine_*), 135.0 (4C, *C*-2, 6*_Ph_*), 132.8 (2C, *C*-1*_Ph_*), 129.8 (2C, *C*-4*_Ph_*), 128.4 (2C, *C*-3, 5*_benzyl_*), 127.8 (4C, *C*-3, 5*_Ph_*), 126.9 (2C, *C*-2, 6*_benzyl_*), 126.8 (1C, *C*-4*_benzyl_*), 122.2 (1C, *C*-7a*_imidazopyridine_*), 111.3 (1C, *C*(CH_3_)_2_), 98.0 (1C, *C*-6*_imidazopyridine_*), 88.2 (1C, *C*-3), 81.2 (1C, *C*-2), 64.7 (1C, O*C*H_2_), 58.2 (1C, *C*-4), 45.3 (1C, *C*H_2_ *_benzyl_*), 38.2 (1C, *C*-1), 29.8 (1C, *C*-5), 26.7 (3C, C(*C*H_3_)_3_), 25.9 (1C, C(*C*H_3_)_2_), 24.2 (1C, C(*C*H_3_)_2_), 18.8 (1C, *C*(CH_3_)_3_), 12.0 (1C, *C*-6). FT-IR (neat) *ṽ* (cm^−1^) = 2978, 2932 (C-H_aliphat_.), 1608, 1582 (C = C_aromat_.), 1111, 1069, 1038 (C-O), 737, 698 (C-H_aromat_., out of plane).

Next, the compound (0.080 g, 0.12 mmol) was dissolved in CH_3_OH (2.5 mL), trifluoroacetic acid (0.32 mL) and H_2_O (0.32 mL) were added. The mixture was heated to 70 °C for 2 d. The solvent was evaporated and the residue was purified by fc (CH_3_CN: H_2_O = 5:95 ⭢ 100:0, 12 mL/min, Biotage^®®^ SNAP Ultra C18, 12 g, V = 20 mL) to afford the alcohol **13** as a colorless solid (R_f_ = 0.20, CH_2_Cl_2_: CH_3_OH = 95:5), yield 0.034 g (72%). C_20_H_21_ClN_4_O_3_ (400.86 g/mol). Purity (HPLC: method B): 99% (t_R_ = 9.53 min). Exact mass (LC-MS-ESI): *m*/*z* calculated for C_20_H_22_ClN_4_O_3_ [M + H]^+^ 401.1375, found 401.1367. ^1^H-NMR (600 MHz, CD_3_OD) δ (ppm) = 8.47 (s, 1H, 2-C*H_imidazopyridine_*), 7.39–7.35 (m, 2H, 2, 6-C*H_benzyl_*), 7.32 (dd, *J* = 8.5, 6.8 Hz, 2H, 3, 5-C*H_benzyl_*), 7.28–7.23 (m, 1H, 4-C*H_benzyl_*), 6.36 (s, 1H, 6-C*H_imidazopyridine_*), 5.48 (s, 0.2H, C*H*_2_Cl_2_, solvent: dichloromethane), 4.83 (s, 1H, 4-C*H*), 4.76 (dd, *J* = 6.7, 1.7 Hz, 1H, 2-C*H*), 4.57 (s, 2H, C*H_2 benzyl_*), 4.27 (dd, *J* = 11.6, 0.9 Hz, 1H, OC*H*H), 3.84 (dt, *J* = 6.7, 1.2 Hz, 1H, 3-C*H*), 3.32 (d, *J* = 11.5 Hz, 1H, OCH*H*), 1.62 (ddd, *J* = 8.8, 3.9, 1.5 Hz, 1H, 5-C*H*), 1.55 (dd, *J* = 5.2, 3.9 Hz, 1H, 6-C*H*H), 0.74 (ddd, *J* = 8.7, 5.2, 1.8 Hz, 1H, 6-CH*H*). ^13^C-NMR (151 MHz, CD_3_OD) δ (ppm) = 149.2 (1C, *C*-7*_imidazopyridine_*), 148.6 (1C, *C*-5*_imidazopyridine_*), 146.1 (1C, *C*-3a*_imidazopyridine_*), 140.3 (1C, *C*-2*_imidazopyridine_*), 139.4 (1C, *C*-1*_benzyl_*), 129.7 (2C, *C*-3, 5*_benzyl_*), 128.4 (2C, *C*-2, 6*_benzyl_*), 128.3 (1C, *C*-4*_benzyl_*), 123.4 (1C, *C*-7a*_imidazopyridine_*), 99.7 (1C, *C*-6*_imidazopyridine_*), 77.6 (1C, *C*-3), 72.3 (1C, *C*-2), 64.4 (1C, O*C*H_2_), 63.3 (1C, *C*-4), 47.5 (1C, *C*H_2_ *_benzyl_*), 38.0 (1C, *C*-1), 24.4 (1C, *C*-5), 12.2 (1C, *C*-6). FT-IR (neat) *ṽ* (cm^−1^) = 3325 (O-H), 2978 (C-H_aliphat_.), 1608, 1578 (C = C_aromat_.), 1119, 1072 (C-O), 733, 694 (C-H_aromat_., out of plane).

*(1R,2R,3S,4R,5S)-4-[7-(Benzylamino)-5-methylthio-3H-imidazo[4,5-b]pyridin-3-yl]-1-(hydroxymethyl)bicyclo[3.1.0]hexane-2,3-diol* (**14**). Compound **13** (0.10 g, 0.15 mmol) was dissolved in DMF (5.5 mL). NaSCH_3_ (0.21 g, 3.04 mmol, 20 eq.) was added. The mixture was stirred at 90 °C for 2 h in the microwave at a power of 200 W. The solvent was evaporated and the residue was purified by fc (CH_3_CN: H_2_O = 5:95 ⭢ 100:0, 12 mL/min, Biotage^®®^ SNAP Ultra C18, 12 g, V = 20 mL). The purified intermediate was dissolved in CH_3_OH (1 mL), trifluoroacetic acid (0.12 mL) and H_2_O (0.12 mL) were added. The mixture was heated to 70 °C for 4 h. The solvent was evaporated and the residue was purified by fc (CH_3_CN: H_2_O = 5:95 ⭢ 100:0, 12 mL/min, Biotage^®®^ SNAP Ultra C18, 12 g, V = 20 mL) to afford the product **93** as a colorless solid alongside an impurity due to the incomplete conversion during formation of the methylthio ether. The mixture was dissolved in DMF (0.5 mL) and NaSCH_3_ (0.041 g, 0.58 mmol, 4 eq.) was added. The mixture was stirred at 90 °C for 1 h in the microwave at a power of 200 W. The solvent was evaporated and the residue was purified by fc (CH_3_CN: H_2_O = 5:95 ⭢ 100:0, 12 mL/min, Biotage^®®^ SNAP Ultra C18, 12 g, V = 20 mL) to afford the product **93** as a beige solid with a small contamination with starting material (R_f_ = 0.29, CH_2_Cl_2_: CH_3_OH = 9:1), yield 0.010 g (16%). C_21_H_24_N_4_O_3_S (412.51 g/mol). Purity (HPLC: method B): 93% (t_R_ = 9.78 min). Exact mass (LC-MS-ESI): *m*/*z* calculated for C_21_H_25_N_4_O_3_S [M + H]^+^ 413.1642, found 413.1643. ^1^H-NMR (400 MHz, DMSO-*d*_6_) δ (ppm) = 8.28 (s, 1H, 2-C*H_imidazopyridine_*), 7.41 (t, *J* = 6.4 Hz, 1H, N*H*), 7.38–7.27 (m, 4H, 2, 3, 5, 6-C*H_benzyl_*), 7.25–7.19 (m, 1H, 4C*H_benzyl_*), 6.12 (s, 1H, 6C*H_imidazopyridine_*), 5.10 (s, 1H, 3-O*H*), 4.98 (s, 1H, CH_2_O*H*), 4.79 (s, 1H, 4-C*H*), 4.58 (s, 3H, 2C*H*, C*H_2 benzyl_*), 4.46 (s, 1H, 2-O*H*), 4.06 (d, *J* = 11.3 Hz, 1H, OC*H*H), 4.03 (q, *J* = 7.1 Hz, 0.3H, C*H*_2_, solvent: ethyl acetate), 3.68 (d, *J* = 6.5 Hz, 1H, 3-C*H*), 3.15 (d, *J* = 11.4 Hz, 1H, OCH*H*), 2.45 (s, 3H, SC*H*_3_), 2.08 (s, 0.1H, C*H*_3_CN, solvent: acetonitrile), 1.99 (s, 0.3H, OC*H*_3_, solvent: ethyl acetate), 1.43 (ddd, *J* = 8.7, 4.6, 1.4 Hz, 1H, 5-C*H*), 1.33 (t, *J* = 4.3 Hz, 1H, 6C*H*H), 1.17 (t, *J* = 7.1 Hz, 0.1H, CH_2_C*H*_3_, solvent: ethyl acetate), 0.59 (ddd, *J* = 8.6, 4.7, 1.6 Hz, 1H, 6-CH*H*); the ^1^HNMR spectrum displayed small impurities in the range of about 5% assigned to compound **13**. ^13^C-NMR (101 MHz, DMSO-*d*_6_) δ (ppm) = 153.9 (1C, *C*-5*_imidazopyridine_*), 146.1 (1C, *C*7*_imidazopyridine_*), 146.0 (1C, *C*-3a*_imidazopyridine_*), 139.7 (1C, *C*-1*_benzyl_*), 137.2 (1C, *C*2*_imidazopyridine_*), 128.3 (2C, *C*-3, 5*_benzyl_*), 127.0 (2C, *C*-2, 6*_benzyl_*), 126.7 (1C, *C*4*_benzyl_*), 120.9 (1C, *C*7a*_imidazopyridine_*), 95.9 (1C, *C*6*_imidazopyridine_*), 76.2 (1C, *C*3), 70.3 (1C, *C*2), 62.3 (1C, O*C*H_2_), 60.2 (1C, *C*-4), 45.4 (1C, *C*H_2_ *_benzyl_*), 36.4 (1C, *C*-1), 23.3 (1C, *C*-5), 13.1 (1C, S*C*H_3_), 11.1 (1C, *C*-6); the ^13^C-NMR spectrum displayed small impurities in the range of about 5% assigned to compound **13**. FT-IR (neat) *ṽ* (cm^−1^) = 3302 (O-H), 2978 (C-H_aliphat_.), 1605, 1582 (C = C_aromat_.), 1115, 1072, 1006 (C-O), 737, 698 (CH_aromat_., out of plane).

*(1R,2R,3S,4R,5S)-4-{5-Chloro-7-[(4-methoxy)benzylamino]-3H-imidazo[4,5-b]pyridin-3-yl}-1-(hydroxymethyl)bicyclo[3.1.0]hexane-2,3-diol* (**15**). Compound **9** (0.41 g, 0.68 mmol) was dissolved in NMP (8 mL). 4-methoxybenzylamine (1.3 mL, 9.95 mmol, 15 eq.) and DIPEA (0.56 mL, 3.29 mmol, 4.5 eq.) were added. The mixture was stirred at 200 °C for 5 min in the microwave at a power of 200 W and was directly purified by fc (CH_3_CN: H_2_O = 5:95 ⭢ 100:0, 50 mL/min, Biotage^®®^ SNAP C18, 120 g, V = 20 mL) to afford the acetonide-protected product (1*R*,2*R*,3*S*,4*R*,5*S*)-4-(7-Amino-5-chloro-3*H*-imidazo[4,5-*b*]pyridin-3-yl)-1-{[(*tert*-butyldiphenylsilyl)oxy]methyl}-2,3-*O*-isopropylidenebicyclo[3.1.0]hexane-2,3-diol as a colorless solid (R_f_ = 0.35, cyclohexane: ethyl acetate = 1:1), yield 0.39 g (80%). C_40_H_45_ClN_4_O_4_Si (708.29 g/mol). Melting point: 82.3 °C. Purity (HPLC: method C): 96% (t_R_ = 17.51 min). Exact mass (LC-MS-ESI): *m*/*z* calculated for C_40_H_46_ClN_4_O_4_Si [M + H]^+^ 709.2971, found 709.2958. ^1^H-NMR (400 MHz, DMSO-*d*_6_) δ (ppm) = 8.24 (s, 1H, 2-C*H_imidazopyridine_*), 7.85 (t, *J* = 5.2 Hz, 1H, N*H*), 7.637.56 (m, 4H, 2, 6-C*H_Ph_*), 7.46–7.32 (m, 6H, 3, 4, 5-C*H_Ph_*), 7.32–7.27 (m, 2H, 2, 6C*H_benzyl_*), 6.91–6.86 (m, 2H, 3, 5-C*H_benzyl_*), 6.29 (s, 1H, 6C*H_imidazopyridine_*), 5.25 (dd, *J* = 7.3, 1.2 Hz, 1H, 2C*H*), 4.91 (s, 1H, 4C*H*), 4.65 (dd, *J* = 7.1, 1.5 Hz, 1H, 3-C*H*), 4.53 (s, 2H, C*H_2 benzyl_*), 4.04 (d, *J* = 10.7 Hz, 1.2H, OC*H*H, C*H*_2_, solvent: ethyl acetate), 3.76 (d, *J* = 10.7 Hz, 1H, OCH*H*), 3.71 (s, 3H, OC*H*_3_), 2.07 (s, 0.1H, C*H*_3_CN, solvent: acetonitrile), 1.99 (s, 0.2H, OC*H*_3_, solvent: ethyl acetate), 1.65 (ddd, *J* = 9.3, 4.5, 1.5 Hz, 1H, 5C*H*), 1.45 (s, 3H, C(C*H*_3_)_2_), 1.18 (s, 3.2H, C(C*H*_3_)_2_, CH_2_C*H*_3_, solvent: ethyl acetate), 1.02 (s, 9H, C(C*H*_3_)_3_), 0.98 (t, *J* = 4.8 Hz, 1H, 6-C*H*H), 0.90 (ddd, *J* = 9.2, 5.2, 1.4 Hz, 1H, 6-CH*H*); the ^1^HNMR spectrum displayed small impurities in the range of about 5%.^13^C-NMR (101 MHz, DMSO-*d*_6_) δ (ppm) = 158.2 (1C, *C*4*_benzyl_*), 147.8 (1C, *C*7*_imidazopyridine_*), 146.0 (1C, *C*5*_imidazopyridine_*), 144.7 * (1C, *C*-3a*_imidazopyridine_*), 138.6 (1C, *C*2*_imidazopyridine_*), 135.0 (4C, *C*-2, 6*_Ph_*), 132.8 (2C, *C*-1*_Ph_*), 131.0 * (1C, *C*-1*_benzyl_*), 129.8 (2C, *C*4*_Ph_*), 128.3 (2C, *C*2, 6*_benzyl_*), 127.8 (4C, *C*3, 5*_Ph_*), 122.2 (1C, *C*7a*_imidazopyridine_*), 113.8 (2C, *C*3*_imidazopyridine_*, 5*_benzyl_*), 111.3 (1C, *C*(CH_3_)_2_), 98.0 * (1C, *C*6*_imidazopyridine_*), 88.2 (1C, *C*3), 81.2 (1C, *C*2), 64.8 (1C, O*C*H_2_, 58.2 (1C, *C*-4), 55.0 (1C, O*C*H_3_), 44.7 (1C, *C*H_2_ *_benzyl_*), 38.2 (1C, *C*-1), 29.8 (1C, *C*-5), 26.7 (3C, C(*C*H_3_)_3_), 25.9 (1C, C(*C*H_3_)_2_), 24.3 (1C, C(*C*H_3_)_2_), 18.8 (1C, *C*(CH_3_)_3_), 12.0 (1C, *C*-6); *: *C*-3a*_imidazopyridine_*, *C*-6*_imidazopyridine_* and *C*-1*_benzyl_* could only be seen in 2D NMR spectra. FT-IR (neat) *ṽ* (cm^−1^) = 3071 (v C-H_aromat_.), 2932 (C-H_aliphat_.), 1609, 1582 (C = C_aromat_.), 1111, 1065, 1038 (C-O), 741, 702 (CH_aromat_., out of plane).

Next, the acetonide-protected compound (0.050 g, 0.07 mmol) was dissolved in CH_3_OH (1.6 mL), trifluoroacetic acid (0.20 mL) and H_2_O (0.20 mL) were added. The mixture was heated to 70 °C for 1 d. The solvent was evaporated and the residue was purified by fc (CH_3_CN: H_2_O = 5:95 ⭢ 100:0, 12 mL/min, Biotage^®®^ SNAP Ultra C18, 12 g, V = 20 mL) to afford the alcohol **95** as a colorless solid (R_f_ = 0.19, CH_2_Cl_2_: CH_3_OH = 95:5), yield 0.027 g (89%). C_21_H_23_ClN_4_O_4_ (430.89 g/mol). Purity (HPLC: method B): 98% (t_R_ = 9.67 min). Exact mass (LC-MS-ESI): *m*/*z* calculated for C_21_H_24_ClN_4_O_4_ [M + H]^+^ 431.1481, found 431.1486.^1^H-NMR (400 MHz, DMSO-*d*_6_ δ (ppm) = 8.44 (s, 1H, 2-C*H_imidazopyridine_*), 7.73 (t, *J* = 6.8 Hz, 1H, N*H*), 7.31–7.25 (m, 2H, 2, 6-C*H_benzyl_*), 6.94–6.83 (m, 2H, 3, 5-C*H_benzyl_*), 6.30 (s, 1H, 6-C*H_imidazopyridine_*), 5.75 (s, 0.4H, C*H*_2_Cl_2_, solvent: dichloromethane), 4.72 (s, 1H, 4-C*H*), 4.58 (dd, *J* = 6.5, 1.5 Hz, 3H, 2-C*H*, C*H_2 benzyl_*), 4.09 (d, *J* = 11.4 Hz, 1H, OC*H*H), 3.71 (s, 3H, OC*H*_3_), 3.66 (dt, *J* = 6.4, 1.2 Hz, 1H, 3-C*H*), 3.15 (d, *J* = 11.4 Hz, 1H, OCH*H*), 1.44 (ddd, *J* = 8.8, 3.9, 1.4 Hz, 1H, 5-C*H*), 1.34 (t, *J* = 4.3 Hz, 1H, 6-C*H*H), 0.60 (ddd, *J* = 8.6, 4.7, 1.5 Hz, 1H, 6-CH*H*). ^13^C-NMR (101 MHz, DMSO-*d*_6_) δ (ppm) = 158.2 (1C, *C*-4*_benzyl_*), 147.7 (1C, *C*-7*_imidazopyridine_*), 145.9 (1C, *C*-5*_imidazopyridine_*), 145.0 (1C, *C*-3a*_imidazopyridine_*), 138.5 (1C, *C*-2*_imidazopyridine_*), 131.0 (1C, *C*-1*_benzyl_*), 128.3 (2C, *C*-2, 6*_benzyl_*), 121.8 (1C, *C*-7a*_imidazopyridine_*), 113.8 (2C, *C*-3, 5*_benzyl_*), 98.0 (1C, *C*-6*_imidazopyridine_*), 76.0 (1C, *C*-3), 70.3 (1C, *C*-2), 62.2 (1C, O*C*H_2_), 60.5 (1C, *C*-4), 55.0 (1C, O*C*H_3_), 54.9 (0.2C, *C*H_2_Cl_2_, solvent: dichloromethane), 44.9 (1C, *C*H_2_ *_benzyl_*), 36.4 (1C, *C*-1), 23.2 (1C, *C*-5), 11.0 (1C, *C*-6). FT-IR (neat) *ṽ* (cm^−1^) = 3318 (O-H), 2920 (C-H_aliphat_.), 1609 (C = C_aromat_.), 1119, 1080, 1030 (C-O), 737 (C-H_aromat_., out of plane).

*(1R,2R,3S,4R,5S)-4-(7-Amino-5-chloro-3H-imidazo[4,5-b]pyridin-3-yl)-1-(hydroxymethyl)bicyclo[3.1.0]hexane-2,3-diol* (**16**). The acetonide-protected intermediate (1*R*,2*R*,3*S*,4*R*,5*S*)-4-(7-amino-5-chloro-3*H*-imidazo[4,5-*b*]pyridin-3-yl)-1-{[(*tert*-butyldiphenylsilyl)oxy]methyl}-2,3-*O*-isopropylidenebicyclo[3.1.0]hexane-2,3-diol (0.26 g, 0.43 mmol) was dissolved in CH_2_Cl_2_ (2.7 mL). H_2_O (0.3 mL) and 2,3-dichloro-5,6-dicyano-1,4-benzoquinone (DDQ, 0.038 g, 0.17 mmol, 1.5 eq.) were added. The mixture was stirred at rt overnight. H_2_O was added and the mixture was extracted three times with CH_2_Cl_2_. The solvent was evaporated and the residue was purified by fc (CH_3_CN: H_2_O = 5:95 ⭢ 100:0, 12 mL/min, Biotage^®®^ SNAP C18, 12 g, V = 20 mL) to afford the amine as a beige solid (R_f_ = 0.19, cyclohexane: ethyl acetate = 1:1), yield 0.049 g (74%). C_32_H_37_ClN_4_O_3_Si (588.23 g/mol). Melting point: 101.0 °C. Purity (HPLC: method B): 98% (t_R_ = 22.35 min). Exact mass (LC-MS-ESI): *m*/*z* calculated for C_32_H_38_ClN_4_O_3_Si [M + H]^+^ 589.2396, found 589.2395. ^1^H-NMR (600 MHz, DMSO-*d*_6_) δ (ppm) = 8.21 (s, 1H, 2-C*H_imidazopyridine_*), 7.63–7.58 (m, 4H, 2, 6C*H_Ph_*), 7.47–7.42 (m, 2H, 4-C*H_Ph_*), 7.41–7.37 (m, 4H, 3, 5-C*H_Ph_*), 6.80 (s, 2H, N*H*_2_), 6.38 (s, 1H, 6-C*H_imidazopyridine_*), 5.75 (s, 0.1H, C*H*_2_Cl_2_, solvent: dichloromethane), 5.27 (dd, *J* = 7.0, 1.3 Hz, 1H, 2-C*H*), 4.90 (s, 1H, 4C*H*), 4.64 (dd, *J* = 7.2, 1.5 Hz, 1H, 3-C*H*), 4.04 (d, *J* = 10.7 Hz, 1H, OC*H*H), 3.74 (d, *J* = 10.7 Hz, 1H, OCH*H*), 1.65 (ddd, *J* = 9.3, 4.4, 1.5 Hz, 1H, 5C*H*), 1.45 (s, 3H, C(C*H*_3_)_2_), 1.19 (s, 3H, C(C*H*_3_)_2_), 1.02 (s, 9H, C(C*H*_3_)_3_), 0.98 (t, *J* = 4.8 Hz, 1H, 6-C*H*H), 0.89 (ddd, *J* = 9.2, 5.1, 1.5 Hz, 1H, 6CH*H*). ^13^C-NMR (151 MHz, DMSO-*d*_6_) δ (ppm) = 148.9 (1C, *C*-7*_imidazopyridine_*), 145.7 (1C, *C*5*_imidazopyridine_*), 145.3 (1C, *C*-3a*_imidazopyridine_*), 138.7 (1C, *C*-2*_imidazopyridine_*), 135.1 (4C, *C*2, 6*_Ph_*), 132.8 (2C, *C*-1*_Ph_*), 129.9 (2C, *C*4*_Ph_*), 127.9 (4C, *C*3, 5*_Ph_*), 122.1 (1C, *C*7a*_imidazopyridine_*), 111.3 (1C, *C*(CH_3_)_2_), 100.5 (1C, *C*6*_imidazopyridine_*), 88.3 (1C, *C*3), 81.2 (1C, *C*2), 64.9 (1C, O*C*H_2_), 58.1 (1C, *C*-4), 38.3 (1C, *C*-1), 29.9 (1C, *C*-5), 26.8 (3C, C(*C*H_3_)_3_), 25.9 (1C, C(*C*H_3_)_2_), 24.3 (1C, C(*C*H_3_)_2_), 18.9 (1C, *C*(CH_3_)_3_), 12.1 (1C, *C*-6). FT-IR (neat) *ṽ* (cm^−1^) = 3360 (N-H), 2978, 2932 (C-H_aliphat_.), 1624, 1601 (C = C_aromat_.), 1111, 1065, 1038 (C-O), 741, 702 (CH_aromat_., out of plane).

Next the acetonide-protected intermediate (0.051 g, 0.09 mmol) was dissolved in CH_3_OH (1.6 mL), trifluoroacetic acid (0.20 mL) and H_2_O (0.20 mL) were added. The mixture was heated to 70 °C for 1 d. The solvent was evaporated and the residue was purified by semi-preparative HPLC (method B) to afford the alcohol **16** as a colorless solid (R_f_ = 0.24, CH_2_Cl_2_: CH_3_OH = 9:1), yield 0.016 g (60%). C_13_H_15_ClN_4_O_3_ (310.74 g/mol). Purity (HPLC: method B): > 99% (t_R_ = 4.11 min). Exact mass (LC-MS-ESI): *m*/*z* calculated for C_13_H_16_ClN_4_O_3_ [M + H]^+^ 311.0905, found 311.0905. ^1^H-NMR (600 MHz, DMSO-*d*_6_) δ (ppm) = 8.45 (s, 1H, 2-C*H_imidazopyridine_*), 6.76 (s, 2H, N*H*_2_), 6.39 (s, 1H, 6-C*H_imidazopyridine_*), 4.72 (s, 1H, 4-C*H*), 4.57 (dd, *J* = 6.5, 1.6 Hz, 1H, 2-C*H*), 4.08 (d, *J* = 11.4 Hz, 1H, OC*H*H), 3.66 (d, *J* = 6.4 Hz, 1H, 3-C*H*), 3.14 (d, *J* = 11.4 Hz, 1H, OCH*H*), 1.44 (ddd, *J* = 8.8, 3.9, 1.4 Hz, 1H, 5-C*H*), 1.34 (t, *J* = 4.3 Hz, 1H, 6-C*H*H), 0.60 (ddd, *J* = 8.5, 4.7, 1.6 Hz, 1H, 6-CH*H*). ^13^C-NMR (151 MHz, DMSO-*d*_6_) δ (ppm) = 148.6 (1C, *C*-5*_imidazopyridine_*), 145.6 (1C, *C*-7*_imidazopyridine_*), 145.4 (1C, *C*-3a*_imidazopyridine_*), 138.7 (1C, *C*-2*_imidazopyridine_*), 121.4 (1C, *C*-7a*_imidazopyridine_*), 100.5 (1C, *C*-6*_imidazopyridine_*), 76.0 (1C, *C*-3), 70.3 (1C, *C*-2), 62.2 (1C, O*C*H_2_), 60.6 (1C, *C*-4), 36.4 (1C, *C*-1), 23.2 (1C, *C*-5), 11.1 (1C, *C*-6). FT-IR (neat) *ṽ* (cm^−1^) = 3345, 3217 (O-H), 2924 (C-H_aliphat_.), 1632, 1601 (C = C_aromat_.), 1115, 1069, 1007 (C-O).

*(1R,2R,3S,4R,5S)-4-(7-Amino-5-methylthio-3H-imidazo[4,5-b]pyridin-3-yl)-1-(hydroxymethyl)bicyclo[3.1.0]hexane-2,3-diol* (**17**). An amount of (1*R*,2*R*,3*S*,4*R*,5*S*)-4-(7-Amino-5-chloro-3*H*-imidazo[4,5-*b*]pyridin-3-yl)-1-{[(*tert*-butyldiphenylsilyl)oxy]methyl}-2,3-*O*-isopropylidenebicyclo[3.1.0]hexane-2,3-diol (0.030 g, 0.05 mmol) was dissolved in DMF (1.5 mL). NaSCH_3_ (0.078 g, 1.11 mmol, 22 eq.) was added. The mixture was stirred at 90 °C for 2 h in the microwave at a power of 200 W. H_2_O was added and the reaction was extracted three times with ethyl acetate. The organic phase was dried over anh. Na_2_SO_4_, filtered and concentrated in vacuo. Due to incomplete conversion, the residue was dissolved in DMF (1.5 mL), and NaSCH_3_ (0.054 g, 0.77 mmol, 15 eq.) was added. The mixture was stirred at 90 °C for 1 h in the microwave at a power of 200 W. H_2_O was added and the reaction was extracted three times with ethyl acetate. The organic phase was dried over anh. Na_2_SO_4_, filtered and concentrated in vacuo. The residue was purified by fc (CH_3_CN: H_2_O = 5:95 ⭢ 100:0, 12 mL/min, Biotage^®®^ SNAP C18, 12 g, V = 20 mL) to afford the methylthio ether as a colorless oil (R_f_ = 0.23, CH_2_Cl_2_: CH_3_OH = 95:5), yield 0.011 g (58%). C_17_H_22_N_4_O_3_S (362.45 g/mol). Melting point: 109.2 °C. Purity (HPLC: method B): 94% (t_R_ = 9.14 min). Exact mass (LC-MS-ESI): *m*/*z* calculated for C_17_H_23_N_4_O_3_S [M + H]^+^ 363.1485, found 363.1493. ^1^H-NMR (600 MHz, DMSO-*d*_6_) δ (ppm) = 8.15 (s, 1H, 2-C*H_imidazopyridine_*), 6.39 (s, 2H, N*H*_2_), 6.27 (s, 1H, 6-C*H_imidazopyridine_*), 5.75 (s, 0.4H, C*H*_2_Cl_2_, solvent: dichloromethane), 5.22 (dd, *J* = 7.0, 1.4 Hz, 1H, 2-C*H*), 4.98 (t, *J* = 5.2 Hz, 1H, O*H*), 4.94 (s, 1H, 4-C*H*), 4.55 (dd, *J* = 7.1, 1.5 Hz, 1H, 3C*H*), 3.86 (dd, *J* = 11.7, 3.2 Hz, 1H, OC*H*H), 3.36–3.31 (m, 1H, OCH*H*), 2.49 (s, 3H, SC*H*_3_), 1.61 (ddd, *J* = 9.2, 4.4, 1.5 Hz, 1H, 5-C*H*), 1.44 (s, 3.3H, C(C*H*_3_)_2_, 1.17 (s, 3.3H, C(C*H*_3_)_2_, 0.98 (t, *J* = 4.8 Hz, 1H, 6-C*H*H), 0.880.84 (m, 1H, 6-CH*H*): ^13^C-NMR (151 MHz, DMSO-*d*_6_) δ (ppm) = 153.9 (1C, *C*-5*_imidazopyridine_*), 147.1 (1C, *C*7*_imidazopyridine_*), 146.1 (1C, *C*-3a*_imidazopyridine_*), 137.5 (1C, *C*-2*_imidazopyridine_*), 121.1 (1C, *C*7a*_imidazopyridine_*), 111.2 (1C, *C*(CH_3_)_2_), 98.3 (1C, *C*6*_imidazopyridine_*), 88.5 (1C, *C*3), 80.8 (1C, *C*2), 62.6 (1C, O*C*H_2_), 57.7 (1C, *C*4), 54.9 (0.2C, *C*H_2_Cl_2_, solvent: dichloromethane), 38.7 (1C, *C*1), 29.8 (1C, *C*5), 25.9 (1C, C(*C*H_3_)_2_), 24.2 (1C, C(*C*H_3_)_2_), 13.2 (1C, S*C*H_3_), 12.6 (1C, *C*6): FT-IR (neat) *ṽ* (cm^−1^) = 3352, 3202 (O-H), 2986, 2924 (C-H_aliphat_.), 1624, 1582 (C = C_aromat_.), 1057, 1026, 1015 (C-O).

Next, the acetonide-protected methylthioether intermediate (0.025 g, 0.07 mmol) was dissolved in CH_3_OH (1.6 mL) and trifluoroacetic acid (0.20 mL) and H_2_O (0.20 mL) were added. The mixture was heated to 50 °C overnight. The solvent was evaporated and the residue was purified by semi-preparative HPLC (method C) to afford the product **17** as a colorless solid (R_f_ = 0.22, CH_2_Cl_2_: CH_3_OH = 9:1), yield 0.012 g (55%). C_14_H_18_N_4_O_3_S (322.38 g/mol). Purity (HPLC: method D): 99% (t_R_ = 10.45 min). Exact mass (LC-MS-ESI): *m*/*z* calculated for C_14_H_19_N_4_O_3_S [M + H]^+^ 323.1172, found 323.1167. ^1^H-NMR (600 MHz, DMSO-*d*_6_) δ (ppm) = 8.43 (s, 1H, 2-C*H_imidazopyridine_*), 6.46 (s, 2H, N*H*_2_), 6.31 (s, 1H, 6-C*H_imidazopyridine_*), 4.81 (s, 1H, 4-C*H*), 4.57 (dd, *J* = 6.6, 1.5 Hz, 1H, 2-C*H*), 4.06 (d, *J* = 11.4 Hz, 1H, OC*H*H), 3.70 (d, *J* = 6.4 Hz, 1H, 3-C*H*), 3.15 (d, *J* = 11.4 Hz, 1H, OCH*H*), 2.54 (s, 0.4H, C*H*_3_, solvent: DMSO), 2.50 (s, 3H, SC*H*_3_), 2.07 (s, 0.1H, C*H*_3_CN, solvent: acetonitrile), 1.45 (ddd, *J* = 8.7, 3.9, 1.3 Hz, 1H, 5C*H*), 1.34 (t, *J* = 4.3 Hz, 1H, 6-C*H*H), 0.61 (ddd, *J* = 8.6, 4.6, 1.5 Hz, 1H, 6-CH*H*). ^13^C-NMR (151 MHz, DMSO-*d*_6_) δ (ppm) = 154.2 (1C, *C*-5*_imidazopyridine_*), 146.5 (1C, *C*7*_imidazopyridine_*), 145.9 (1C, *C*3a*_imidazopyridine_*), 137.2 (1C, *C*-2*_imidazopyridine_*), 119.4 (1C, *C*7a*_imidazopyridine_*), 98.7 (1C, *C*6*_imidazopyridine_*), 76.0 (1C, *C*3), 70.2 (1C, *C*2), 62.2 (1C, O*C*H_2_), 60.4 (1C, *C*-4), 40.5 (0.1C, *C*H_3_, solvent: DMSO), 36.4 (1C, *C*1), 23.2 (1C, *C*-5), 13.1 (1C, S*C*H_3_), 11.2 (1C, *C*6). FT-IR (neat) *ṽ* (cm^−1^) = 3341, 3217 (O-H), 2920 (C-H_aliphat_.), 1674, 1628, 1597 (C = C_aromat_.), 1119, 1069, 1011 (C-O).

*5-Chloro-6-nitro-3H-imidazo[4,5-b]pyridine* (**19**). 2-Chloro-1-deazapurine (0.10 g, 0.66 mmol) and di-*tert*-butyl dicarbonate (0.20 g, 0.91 mmol, 1.4 eq.) were suspended in CH_2_Cl_2_ (1 mL). A catalytic amount of DMAP (~1 mg) was added and the mixture was stirred for 2.5 h. The reaction was quenched with silica gel and filtered through a pad of Celite^®®^. The mixture was concentrated in vacuo and the residue was redissolved in CH_2_Cl_2_ (2 mL). Tetrabutylammonium nitrate (0.30 g, 0.98 mmol, 1.5 eq.) was added and the mixture cooled to 0 °C with an ice bath. Trifluoroacetic anhydride (0.14 mL, 0.99 mmol, 1.5 eq.) was added dropwise and the reaction stirred for 5 h at rt and under reflux overnight. The solvent was evaporated, and the residue was purified by fc (CH_2_Cl_2_:CH_3_OH = 97.5:2.5 ⭢ 96.5:3.5 ⭢ 95.5, Ø = 3 cm, l = 24 cm, V = 10 mL) to afford the product **19** as light brown solid (76%). C_6_H_3_ClN_4_O_2_ (198.57 g/mol). ^1^H-NMR (400 MHz, DMSO-*d*_6_) δ (ppm) = 8.87 (s, 1H, 7-C*H*), 8.80 (s, 1H, 2-C*H*), 3.17 (s, 0.4H, C*H*_3_OH, solvent: methanol). ^13^C-NMR (101 MHz, DMSO-*d*_6_) δ (ppm) = 149.6 (1C, *C*-2), 139.9 (1C, *C*-5), 135.6 (1C, *C*-6), 48.6 (0.1H, *C*H_3_OH, solvent: methanol); C-3a, C-7 and C-7a were not visible.

Crystal data for C_6_H_3_ClN_4_O_2_ (M = 198.57 g/mol): orthorhombic, Pbca (No. 61), a = 11.3385(3) Å, b = 6.5407(2) Å, c = 20.0740(7) Å, V = 1488.72(8) Å^3^, Z = 8, 1.772 mg/m^3^, T = 173(2) K, μ(CuKα) = 0.479 mm^−1^, final R indices [I > 2σ(I)] R^1^ = 0.0316, wR^2^ = 0.0798, R indices (all data) R^1^ = 0.0355, wR^2^ = 0.0827

*7-Chloro-5-nitro-3-tosyl-3H-imidazo[4,5-b]pyridine* (**20**). Compound **7** (0.50 g, 2.5 mmol) was suspended in CH_2_Cl_2_ (20 mL). Tosyl chloride (0.97 g, 5.1 mmol, 2 eq.) and DIPEA (0.88 mL, 5.1 mmol, 2 eq.) were added and the mixture stirred for 3 h at rt. The reaction was neutralized with saturated NH_4_Cl solution and was extracted with CH_2_Cl_2_. The organic phase was dried over anh. Na_2_SO_4_ and concentrated in vacuo. The residue was purified by fc (cyclohexane: ethyl acetate = 5:1 ⭢ 2:1, Ø = 6 cm, l = 22 cm, V = 65 mL). The mixed fractions were purified again using fc (cyclohexane: ethyl acetate = 5:1, Ø = 6 cm, l = 22 cm, V = 65 mL) to afford the product **20** as a colorless solid (R_f_ = 0.23, cyclohexane: ethyl acetate = 3:1), yield 0.81 g (91%). C_13_H_9_ClN_4_O_4_S (352.75 g/mol). Melting point: 200.2 °C. Purity (HPLC: method B): > 99% (t_R_ = 15.72 min). Exact mass (APCI): *m*/*z* calculated for C_13_H_10_ClN_4_O_4_S [M + H]^+^ 353.0106, found 353.0105. ^1^H-NMR (400 MHz, CD_3_CN) δ (ppm) = 8.88 (s, 1H, 2-CH), 8.39 (s, 1H, 6-CH), 8.228.18 (m, 2H, 2, 6-CH_tosyl_), 7.48–7.43 (m, 2H, 3, 5-CH_tosyl_), 2.40 (s, 3H, CH_3_). ^13^C-NMR (101 MHz, CD_3_CN) δ (ppm) = 153.6 (1C, C-5), 148.9 (1C, C4_tosyl_), 147.9 (1C, C-2), 144.0 (1C, C-3a), 139.1 (1C, C7a), 138.5 (1C, C-7), 133.9 (1C, C-1_tosyl_), 131.3 (2C, C3, 5_tosyl_), 130.0 (2C, C-2, 6_tosyl_), 116.7 (1C, C6), 21.8 (1C, CH_3_). FT-IR (neat) *ṽ* (cm^−1^) = 3117, 3102 (v C-H_aromat_.), 2978 (C-H_aliphat_.), 1598, 1555 (C = C_aromat_.), 1373, 1327 (NO_2_), 1176 (S = O), 837, 814 (CH_aromat_., out of plane).

*N,N-Dibenzyl-N’-[4-chloro-2-(4-methylphenyl)sulfonamido-6-nitropyridin-3-yl]formimidamide* (**21**). Compound **20** (0.074 g, 0.21 mmol) was dissolved in CH_2_Cl_2_ (2 mL). Dibenzylamine (0.40 mL, 2.08 mmol, 10 eq.) was added and the mixture stirred overnight at rt. The reaction was washed with saturated NH_4_Cl solution and was extracted with CH_2_Cl_2_. The organic phase was dried over anh. Na_2_SO_4_ and concentrated in vacuo. The residue was purified by fc (cyclohexane:ethylacetate:CH_3_OH = 25:3:2 + 1% triethylamine, Ø = 2 cm, l = 25 cm, V = 10 mL) to afford the product **21** as a red solid. Red solid (R_f_ = 0.36, ethyl acetate = 100%), yield 0.090 g (78%). C_27_H_24_ClN_5_O_4_S (550.03 g/mol). Melting point: 171.2 °C. Purity (HPLC: method B): > 99% (t_R_ = 21.02 min). Exact mass (LC-MS-ESI): *m*/*z* calculated for C_27_H_25_ClN_5_O_4_S [M + H]^+^ 550.1310, found 550.1285. ^1^H-NMR (600 MHz, DMSO-*d*_6_) δ (ppm) = 10.30 (s, 1H, N*H*), 8.44 (s, 1H, N = C*H*), 8.09 (d, 2H, *J* = 8.1 Hz, 2, 6-C*H_tosyl_*), 7.92 (s, 1H, 5-C*H_pyridine_*), 7.43–7.26 (m, 12H, 3, 5C*H_tosyl_*, 2, 3, 4, 5, 6C*H_benzyl_*), 4.68 (s, 2H, C*H_2 benzyl_*), 4.46 (s, 2H, C*H_2 benzyl_*), 2.34 (s, 3H, C*H*_3_); the ^1^HNMR spectrum displayed small impurities in the range of about 5%. ^13^C-NMR (151 MHz, DMSO-*d*_6_) δ (ppm) = 157.6 (1C, *C*-1), 147.1 (1C, *C*-6*_pyridine_*), 142.9 (1C, *C*4*_tosyl_*), 139.8 (1C, *C*-4*_pyridine_*), 136.5 (3C, *C*-1*_tosyl_*, *C*-1*_benzyl_*), 132.8 (1C, *C*-3*_pyridine_*), 128.6 (2C, *C*3, 5*_tosyl_*), 128.5 (4C, *C*3, 5*_benzyl_*), 128.4 (2C, *C*-2, 6*_tosyl_*), 128.2 (2C, *C*2, 6*_benzyl_*), 128.0 (2C, *C*2, 6*_benzyl_*), 127.8 (1C, *C*4*_benzyl_*), 127.2 (1C, *C*4*_benzyl_*), 112.8 (1C, *C*5*_pyridine_*), 53.6 (1C, *C*H_2_ *_benzyl_*), 47.1 (1C, *C*H_2_ *_benzyl_*), 21.0 (1C, *C*H_3_); the signal for *C*-4*_pyridine_* could not be seen in ^13^CNMR spectrum. FT-IR (neat) *ṽ* (cm^−1^) = 3251 (N-H), 2978, 2924 (C-H_aliphat_.), 1616 (C = C_aromat_.), 1327(NO_2_), 1161 (S = O), 829, 814, 748, 737 (CH_aromat_., out of plane).

Crystal data for C_27_H_24_ClN_5_O_4_S (M = 550.02 g/mol): monoclinic, P21/n (No. 14), a = 13.4033(2) Å, b = 9.4786(2) Å, β = 98.623(1)°, c = 20.9689(4) Å, V = 2633.87(8) Å^3^, Z = 4, 1.387 mg/m^3^, T = 173(2), μ(CuKα) = 0.268 mm^−1^, Final R indices [I > 2σ(I)]R^1^ = 0.0424, wR^2^ = 0.0934, R indices (all data) R^1^ = 0.0502, wR^2^ = 0.0992

*(1R,2R,3S,4R,5S)-4-[6-(Dibenzylamino)-9H-purin-9-yl]-1-{[(tert-butyldiphenylsilyl)oxy]methyl}-2,3-O-isopropylidenebicyclo[3.1.0]hexane-2,3-diol* (**24**). An amount of 6-chloropurine (**22**, 1.00 g, 6.47 mmol) was suspended in isopropanol (60 mL). Dibenzylamine (5.0 mL, 26.0 mmol, 4 eq.) was added. The mixture was stirred at 90 °C under reflux for 7 h. The solvent was evaporated and the residue was purified by fc (CH_2_Cl_2_:CH_3_OH = 59:1 ⭢ 29:1 + 0.5% HCOOH, Ø = 6 cm, l = 20 cm, V = 65 mL) to afford the *N*,*N*-dibenzyladenine as a colorless solid (R_f_ = 0.36, ethyl acetate = 100%), yield 1.91 g (94%). C_19_H_17_N_5_ (315.38 g/mol). Melting point: 186.4 °C. Purity (HPLC: method B): > 99% (t_R_ = 15.31 min). Exact mass (APCI): *m*/*z* calculated for C_19_H_18_N_5_ [M + H]^+^ 316.1557, found 316.1568. ^1^H-NMR (400 MHz, DMSO-*d*_6_) δ (ppm) = 13.14 (s, 1H, 9-N*H*), 8.28 (s, 1H, 2-C*H*), 8.14 (s, 1H, 8-C*H*), 7.46–7.34 (m, 10H, 2, 3, 4, 5, 6-C*H_benzyl_*), 5.50 (s, 2H, C*H_2 benzyl_*), 4.94 (s, 2H, C*H_2 benzyl_*). ^13^C-NMR (101 MHz, DMSO-*d*_6_) δ (ppm) = 154.1 (1C, *C*-6), 151.9 (1C, *C*-2), 151.6 (1C, *C*-4), 138.5 (1C, *C*-8), 138.0 (1C, *C*1*_benzyl_*), 128.5 (4C, *C*3, 5*_benzyl_*), 127.4 (4C, *C*2, 6*_benzyl_*), 127.0 (2C, *C*4*_benzyl_*), 118.5 (1C, *C*-5), 50.6 (1C, *C*H_2_ *_benzyl_*), 48.5 (1C, *C*H_2_ *_benzyl_*). FT-IR (neat) *ṽ* (cm^−1^) = 3059 (v C-H_aromat_.), 2978, (C-H_aliphat_.), 1574 (C = C_aromat_.), 752, 737, 698 (CH_aromat_., out of plane).

The *N*,*N*-dibenzyladenine (0.47 g, 1.48 mmol, 1.3 eq.) and triphenylphospane (0.47 g, 1.78 mmol, 1.6 eq.) were dissolved in THF (10 mL) under nitrogen atmosphere. Diisopropyl azodicarboxylate (DIAD, 0.34 mL, 1.73 mmol, 1.5 eq.) was added dropwise at 0 °C. The mixture was stirred for 30 min at rt. A solution of the alcohol **4** (0.50 g, 1.15 mmol) in THF (10 mL) was added and the solution was stirred overnight. The solvent was evaporated and the residue was purified by fc (cyclohexane:ethyl acetate = 19:1 ⭢ 9:1, Ø = 6 cm, l = 20 cm, V = 65 mL) to afford the product **24** as a colorless solid (R_f_ = 0.29, cyclohexane:ethyl acetate = 9:1), yield 0.73 g (86%). C_45_H_49_N_5_O_3_Si (736.00 g/mol). Melting point: 77.6 °C. Purity (HPLC: method C): >99% (t_R_ = 18.82 min). Exact mass (APCI): *m*/*z* calculated for C_45_H_50_N_5_O_3_Si [M + H]^+^ 736.3677, found 736.3707. ^1^H-NMR (600 MHz, DMSO-*d*_6_) δ (ppm) = 8.30 (s, 1H, 8-C*H_purine_*), 8.25 (s, 1H, 2-C*H_purine_*), 7.60–7.55 (m, 4H, 2, 6-C*H_Ph_*), 7.42–7.37 (m, 2H, 4-C*H_Ph_*), 7.36–7.32 (m, 4H, 3, 5-C*H_Ph_*), 7.31–7.24 (m, 10H, 2, 3, 4, 5, 6-C*H_benzyl_*), 5.60 (s, 1H, C*H*H*_benzyl_*), 5.47 (s, 1H, CH*H_benzyl_*), 5.28 (dd, *J* = 7.0, 1.3 Hz, 1H, 2-C*H*), 4.98 (s, 1H, 4-C*H*), 4.96 (s, 1H, C*H*H*_benzyl_*), 4.88 (s, 1H, CH*H_benzyl_*), 4.76 (dd, *J* = 7.1, 1.4 Hz, 1H, 3-C*H*), 4.04 (d, *J* = 10.7 Hz, 1H, OC*H*H), 3.72 (d, *J* = 10.8 Hz, 1H, OCH*H*), 1.67 (ddd, *J* = 9.3, 4.5, 1.6 Hz, 1H, 5-C*H*), 1.46 (s, 3H, C(C*H*_3_)_2_), 1.39 (s, 1.6H, C*H*_2_, solvent: cyclohexane), 1.20 (s, 3H, C(C*H*_3_)_2_), 1.01 (t, *J* = 4.8 Hz, 1H, 6-C*H*H), 0.98 (s, 9H, C(C*H*_3_)_3_), 0.90 (ddd, *J* = 9.1, 5.1, 1.5 Hz, 1H, 6-CH*H*). ^13^C-NMR (151 MHz, DMSO-*d*_6_) δ (ppm) = 154.1 (1C, *C*-6*_purine_*), 152.0 (1C, *C*-2*_purine_*), 150.0 (1C, *C*-4*_purine_*), 138.5 (1C, *C*-8*_purine_*), 137.9 (2C, *C*-1*_benzyl_*), 135.1 (4C, *C*-2, 6*_Ph_*), 132.8 (2C, *C*-1*_Ph_*), 129.8 (2C, *C*-4*_Ph_*), 128.5 (4C, *C*-3, 5*_benzyl_*), 127.8 (4C, *C*-3, 5*_Ph_*), 127.4 (4C, *C*-2, 6*_benzyl_*), 127.1 (2C, *C*-4*_benzyl_*), 119.0 (1C, *C*-5*_purine_*), 111.3 (1C, *C*(CH_3_)_2_), 87.9 (1C, *C*-2), 81.1 (1C, *C*-3), 64.7 (1C, O*C*H_2_), 58.4 (1C, *C*-4), 50.8 (1C, *C*H_2_ *_benzyl_*), 48.6 (1C, *C*H_2_ *_benzyl_*), 38.1 (1C, *C*-1), 29.9 (1C, *C*-5), 26.7 (3C, C(*C*H_3_)_3_), 26.3 (s, 0.8C, *C*H_2_, solvent: cyclohexane), 25.9 (1C, C(C*H*_3_)_2_), 24.3 (1C, C(C*H*_3_)_2_), 18.8 (1C, *C*(CH_3_)_3_), 12.2 (1C, *C*-6). FT-IR (neat) *ṽ* (cm^−1^) = 2978 (C-H_aliphat_.), 1574 (C = C_aromat_.), 1107, 1064, 1037 (C-O), 737, 698 (C-H_aromat_., out of plane).

*(1R,2R,3S,4R,5S)-4-[6-(Dibenzylamino)-9H-purin-9-yl]-1-(hydroxymethyl)bicyclo[3.1.0]hexane-2,3-diol* (**26**). Compound **24** (0.125 g, 0.17 mmol) was dissolved in CH_3_OH (3.6 mL) and trifluoroacetic acid (0.40 mL) and H_2_O (0.40 mL) were added. The mixture was heated to 70 °C for 2 d. The solvent was evaporated and the residue was purified by fc (CH_3_CN:H_2_O = 5:95 ⭢ 100:0, 12 mL/min, Biotage^®®^ SNAP Ultra C18, 12 g, V = 20 mL) to afford the alcohol **106** as a colorless solid (R_f_ = 0.24, ethyl acetate = 100%), yield 0.051 g (65%). C_26_H_27_N_5_O_3_ (457.53 g/mol). Purity (HPLC: method B): > 99% (t_R_ = 11.99 min). Exact mass (LC-MS-ESI): *m*/*z* calculated for C_26_H_28_N_5_O_3_ [M + H]^+^ 458.2187, found 458.2187. ^1^H-NMR (600 MHz, DMSO-*d*_6_) δ (ppm) = 8.49 (s, 1H, 8-C*H_purine_*), 8.31 (s, 1H, 2C*H_purine_*), 7.347.29 (m, 4H, 3, 5-C*H_benzyl_*), 7.29–7.23 (m, 6H, 2, 4, 6-C*H_benzyl_*), 5.63 (s, 1H, C*H*H*_benzyl_*), 5.42 (s, 1H, CH*H_benzyl_*), 5.25 (s, 1H, 3-O*H*), 5.03 (t, *J* = 5.0 Hz, 1H, CH_2_O*H*), 4.97 (s, 1H, C*H*H*_benzyl_*), 4.82 (s, 2H, CH*H_benzyl_*, 4-C*H*), 4.58 (t, *J* = 5.2 Hz, 1H, 2-C*H*), 4.49 (t, *J* = 6.8 Hz, 1H, 2-O*H*), 4.07 (dd, *J* = 11.4, 4.9 Hz, 1H, OC*H*H), 3.72 (d, *J* = 6.4 Hz, 1H, 3C*H*), 3.13 (dd, *J* = 11.4, 4.1 Hz, 1H, OCH*H*), 1.49 (ddd, *J* = 8.7, 3.9, 1.4 Hz, 1H, 5C*H*), 1.37 (dd, *J* = 4.7, 3.9 Hz, 1H, 6C*H*H), 0.61 (ddd, *J* = 8.5, 4.7, 1.6 Hz, 1H, 6CH*H*). ^13^C-NMR (151 MHz, DMSO-*d*_6_) δ (ppm) = 154.1 (1C, *C*-6*_purine_*), 151.9 (1C, *C*-2*_purine_*), 150.1 (1C, *C*-4*_purine_*), 138.3 (1C, *C*-8*_purine_*), 137.9 (2C, *C*-1*_benzyl_*), 128.5 (4C, *C*3, 5*_benzyl_*), 127.4 (4C, *C*2, 6*_benzyl_*), 127.1 (2C, *C*-4*_benzyl_*), 118.9 (1C, *C*-5*_purine_*), 75.9 (1C, *C*-3), 70.2 (1C, *C*2), 62.3 (1C, O*C*H_2_), 60.8 (1C, *C*-4), 50.7 (1C, *C*H_2_ *_benzyl_*), 48.6 (1C, *C*H_2_ *_benzyl_*), 36.4 (1C, *C*-1), 23.1 (1C, *C*5), 11.2 (1C, *C*-6). FT-IR (neat) *ṽ* (cm^−1^) = 3310 (O-H), 3028 (v C-H_aromat_.), 2978, 2920 (C-H_aliphat_.), 1578 (C = C_aromat_.), 1068 (C-O), 698 (CH_aromat_., out of plane).

*(1R,2R,3S,4R,5S)-4-[6-(Dibenzylamino)-2-chloro-9H-purin-9-yl]-1-{[(tert-butyldiphenylsilyl)oxy]methyl}-2,3-O-isopropylidenebicyclo[3.1.0]hexane-2,3-diol* (**25**). An amount of 2,6-dichloropurine (2.02 g, 10.47 mmol) was dissolved in isopropanol (100 mL). Dibenzylamine (8.0 mL, 41.6 mmol, 3.9 eq.) was added. The mixture was stirred at 90 °C under reflux for 1.5 h. The precipitated product was filtered off and purified by fc (CH_2_Cl_2_:CH_3_OH = 59:1 ⭢ 29:1 + 0.5% HCOOH, Ø = 8 cm, l = 20 cm, V = 100 mL) to afford the purine derivative as a colorless solid (R_f_ = 0.37, cyclohexane:ethyl acetate = 1:1), yield 3.27 g (88%). C_19_H_16_ClN_5_ (349.82 g/mol). Melting point: 260.0 °C. Purity (HPLC: method B): > 99% (t_R_ = 16.17 min). Exact mass (APCI): *m*/*z* calculated for C_19_H_17_ClN_5_ [M + H]^+^ 350.1167, found 350.1167. ^1^H-NMR (400 MHz, DMSO-*d*_6_) δ (ppm) = 13.29 (s, 1H, 9-N*H*), 8.16 (s, 1H, 8-C*H*), 7.367.30 (m, 4H, 3, 5-C*H_benzyl_*), 7.30–7.24 (m, 6H, 2, 4, 6-C*H_benzyl_*), 5.53 (s, 2H, C*H_2 benzyl_*), 4.81 (s, 2H, C*H_2 benzyl_*). ^13^C-NMR (101 MHz, DMSO-*d*_6_) δ (ppm) = 154.5 (1C, *C*-6), 152.8 (1C, *C*-4), 152.4 (1C, *C*-2), 139.2 (1C, *C*-8), 137.3 (1C, *C*1*_benzyl_*), 128.5 (4C, *C*3, 5*_benzyl_*), 127.5 (4C, *C*2, 6*_benzyl_*), 127.2 (2C, *C*4*_benzyl_*), 117.6 (1C, *C*5), 50.8 (1C, *C*H_2_ *_benzyl_*), 48.9 (1C, *C*H_2_ *_benzyl_*). FT-IR (neat) *ṽ* (cm^−1^) = 3066 (v C-H_aromat_.), 2978, (C-H_aliphat_.), 1578 (C = C_aromat_.), 1076 (C-Cl), 741, 694 (CH_aromat_., out of plane).

Next, the purine derivative (1.04 g, 2.98 mmol, 1.3 eq.) and triphenylphospane (0.90 g, 3.44 mmol, 1.5 eq.) were dissolved in THF (20 mL) under nitrogen atmosphere. DIAD (0.67 mL, 3.41 mmol, 1.5 eq.) was added dropwise at 0 °C. The mixture was stirred for 15 min at rt. A solution of the alcohol **4** (1.03 g, 2.35 mmol) in THF (20 mL) was added and the solution was stirred overnight. The solvent was evaporated and the residue was purified by fc (cyclohexane:ethyl acetate = 19:1 ⭢ 9:1, Ø = 6 cm, l = 20 cm, V = 65 mL) to afford the product **26** as a colorless solid (R_f_ = 0.35, cyclohexane:ethyl acetate = 1:1), yield 1.67 g (92%). C_45_H_48_ClN_5_O_3_Si (770.45 g/mol). Melting point: 84.7 °C. Purity (HPLC: method C): >99% (t_R_ = 19.49 min). Exact mass (LC-MS-ESI): *m*/*z* calculated for C_45_H_49_ClN_5_O_3_Si [M + H]^+^ 770.3288, found 770.3285. ^1^H-NMR (600 MHz, DMSO-*d*_6_) δ (ppm) = 8.29 (s, 1H, 8-C*H_purine_*), 7.60–7.56 (m, 4H, 2, 6-C*H_Ph_*), 7.42–7.25 (m, 16H, 2, 3, 4, 5, 6-C*H_benzyl_*, 3, 4, 5-C*H_Ph_*), 5.57 (d, *J* = 15.7 Hz, 1H, C*H*H*_benzyl_*), 5.47 (d, *J* = 15.7 Hz, 1H, CH*H_benzyl_*), 5.21 (dd, *J* = 7.1, 1.3 Hz, 1H, 2-C*H*), 4.91 (s, 1H, 4-C*H*), 4.86 (d, *J* = 15.4 Hz, 1H, C*H*H*_benzyl_*), 4.79 (d, *J* = 15.4 Hz, 1H, CH*H_benzyl_*), 4.75 (dd, *J* = 7.2, 1.5 Hz, 1H, 3-C*H*), 4.03 (d, *J* = 10.7 Hz, 1H, OC*H*H), 3.87 (d, *J* = 10.7 Hz, 1H, OCH*H*), 1.64 (ddd, *J* = 9.2, 4.5, 1.6 Hz, 1H, 5-C*H*), 1.46 (s, 3H, C(C*H*_3_)_2_), 1.43 (s, 0.7H, C*H*_2_, solvent: cyclohexane), 1.19 (s, 3H, C(C*H*_3_)_2_), 0.98 (s, 10H, 6-C*H*H, C(C*H*_3_)_3_), 0.93 (ddd, *J* = 9.1, 5.1, 1.4 Hz, 1H, 6-CH*H*). ^13^C-NMR (151 MHz, DMSO-*d*_6_) δ (ppm) = 154.5 (1C, *C*-6*_purine_*), 152.5 (1C, *C*-2*_purine_*), 151.1 (1C, *C*-4*_purine_*), 139.3 (1C, *C*-8*_purine_*), 137.4 (1C, *C*-1*_benzyl_*), 136.9 (1C, *C*-1*_benzyl_*), 135.0 (4C, *C*-2, 6*_Ph_*), 132.9 (2C, *C*-1*_Ph_*), 129.8 (2C, *C*-4*_Ph_*), 128.6 (4C, *C*-2, 6*_benzyl_*), 127.8 (4C, *C*-3, 5*_Ph_*), 127.7 (2C, *C*-2, 6*_benzyl_*), 127.5 (1C, *C*-2, 6*_benzyl_*), 127.3 (2C, *C*-4*_benzyl_*), 118.3 (1C, *C*-5*_purine_*), 111,3 (1C, *C*(CH_3_)_2_), 87.9 (1C, *C*-3), 81.6 (1C, *C*-2), 64.5 (1C, O*C*H_2_), 58.9 (1C, *C*-4), 50.9 (1C, *C*H_2_ *_benzyl_*), 49.2 (1C, *C*H_2_ *_benzyl_*), 38.3 (1C, *C*-1), 29.6 (1C, *C*-5), 26.7 (3C, C(*C*H_3_)_3_), 26.3 (0.4C, *C*H_2_, solvent: cyclohexane), 25.9 (1C, C(*C*H_3_)_2_), 24.3 (1C, C(*C*H_3_)_2_), 18.8 (1C, *C*(CH_3_)_3_), 12.0 (1C, *C*-6). FT-IR (neat) *ṽ* (cm^−1^) = 2978 (C-H_aliphat_.), 1574 (C = C_aromat_.), 1111, 1069, 1042 (C-O), 740, 698 (C-H_aromat_., out of plane).

*(1R,2R,3S,4R,5S)-4-[6-(Dibenzylamino)-2-chloro-9H-purin-9-yl]-1-(hydroxymethyl)bicyclo[3.1.0]hexane-2,3-diol* (**27**). Compound **25** (0.099 g, 0.13 mmol) was dissolved in CH_3_OH (3.6 mL), trifluoroacetic acid (0.40 mL) and H_2_O (0.40 mL) were added. The mixture was heated to 70 °C for 2 d. The solvent was evaporated and the residue was purified by fc (CH_2_Cl_2_:CH_3_OH = 96:4, Ø = 2 cm, l = 24 cm, V = 10 mL) but still showed a small impurity by ^1^H-NMR. The impure product was purified again by fc (CH_3_CN:H_2_O = 5:95 ⭢ 100:0, 12 mL/min, Biotage^®®^ SNAP Ultra C18, 12 g, V = 20 mL) to afford the pure alcohol **27** as a colorless solid (R_f_ = 0.32, ethyl acetate = 100%), yield 0.052 g (82%). C_26_H_26_ClN_5_O_3_ (491.98 g/mol). Purity (HPLC: method B): > 99% (t_R_ = 14.14 min). Exact mass (APCI): *m*/*z* calculated for C_26_H_27_ClN_5_O_3_ [M + H]^+^ 492.1797, found 492.1784. ^1^H-NMR (600 MHz, DMSO-*d*_6_) δ (ppm) = 8.51 (s, 1H, 8-C*H_purine_*), 7.36–7.31 (m, 4H, 3, 5-C*H_benzyl_*), 7.30–7.25 (m, 6H, 2, 4, 6-C*H_benzyl_*), 5.61 (d, *J* = 15.7 Hz, 1H, C*H*H*_benzyl_*), 5.41 (d, *J* = 15.8 Hz, 1H, CH*H_benzyl_*), 5.27 (s, 1H, 3-O*H*), 4.99 (t, *J* = 5.0 Hz, 1H, CH_2_O*H*), 4.88 (d, *J* = 15.4 Hz, 1H, C*H*H*_benzyl_*), 4.75 (s, 1H, CH*H_benzyl_*), 4.72 (s, 1H, 4-C*H*), 4.56 (d, *J* = 6.5 Hz, 1H, 2-C*H*), 4.50 (s, 1H, 2-O*H*), 4.07 (d, *J* = 11.0 Hz, 1H, OC*H*H), 3.73 (d, *J* = 6.2 Hz, 1H, 3-C*H*), 3.13 (d, *J* = 11.3 Hz, 1H, OCH*H*), 1.47 (ddd, *J* = 8.8, 3.8, 1.5 Hz, 1H, 5-C*H*), 1.36 (t, *J* = 4.3 Hz, 1H, 6-C*H*H), 0.61 (ddd, *J* = 8.6, 4.7, 1.5 Hz, 1H, 6-CH*H*). ^13^C-NMR (151 MHz, DMSO-*d*_6_) δ (ppm) = 154.5 (1C, *C*-6*_purine_*), 152.5 (1C, *C*-2*_purine_*), 151.4 (1C, *C*-4*_purine_*), 138.9 (1C, *C*-8*_purine_*), 137.4 (1C, *C*-1*_benzyl_*), 136.9 (1C, *C*-1*_benzyl_*), 128.6 (4C, *C*-3, 5*_benzyl_*), 127.7 (2C, *C*-2, 6*_benzyl_*), 127.4 (2C, *C*-2, 6*_benzyl_*), 127.3 (2C, *C*-4*_benzyl_*), 118.0 (1C, *C*-5*_purine_*), 75.8 (1C, *C*-3), 70.2 (1C, *C*-2), 62.2 (1C, O*C*H_2_), 61.0 (1C, *C*-4), 50.8 (1C, *C*H_2_ *_benzyl_*), 49.1 (1C, *C*H_2_ *_benzyl_*), 36.4 (1C, *C*-1), 23.1 (1C, *C*-5), 11.1 (1C, *C*-6).

FT-IR (neat) *ṽ* (cm^−1^) = 3341 (O-H), 2978 (C-H_aliphat_.), 1574 (C = C_aromat_.), 1069 (C-O), 698 (C-H_aromat_., out of plane).

*(1R,2R,3S,4R,5S)-4-[6-(Dibenzylamino)-2-methylthio-9H-purin-9-yl]-1-(hydroxymethyl)-2,3-O-isopropylidenebicyclo[3.1.0]hexane-2,3-diol* (**28**). Compound **25** (0.30 g, 0.39 mmol) was dissolved in DMF (15 mL). NaSCH_3_ (0.41 g, 5.86 mmol, 15 eq.) was added. The mixture was stirred at 90 °C for 1 h in the microwave at a power of 200 W. The solvent was evaporated and the residue was purified by fc (cyclohexane:ethyl acetate = 6:4, Ø = 5 cm, l = 24 cm, V = 30 mL) to afford the product **28** as a colorless solid (R_f_ = 0.35, cyclohexane:ethyl acetate = 1:1), yield 0.227 g (89%). C_30_H_33_N_5_O_3_S (543.69 g/mol). Melting point: 97.8 °C. Purity (HPLC: method B): 93% (t_R_ = 18.60 min). Exact mass (LC-MS-ESI): *m*/*z* calculated for C_30_H_34_N_5_O_3_S [M + H]^+^ 544.2377, found 544.2378. ^1^H-NMR (400 MHz, DMSO-*d*_6_) δ (ppm) = 8.24 (s, 1H, 8-C*H_purine_*), 7.36–7.22 (m, 10H, 2, 3, 4, 5, 6-C*H_benzyl_*), 5.11 (dd, *J* = 15.6, 15.1 Hz, 2H, C*H_2 benzyl_*), 5.20 (d, *J* = 7.1, 1.3 Hz, 1H, 2C*H*), 4.94 (s, 1H, O*H*), 4.92 (s, 1H, 4-C*H*), 4.91–4.74 (m, 2H, C*H_2 benzyl_*), 4.61 (dd, *J* = 7.2, 1.5 Hz, 1H, 3-C*H*), 3.84 (dd, *J* = 11.6, 4.0 Hz, 1H, OCH*H*), 3.35 (d, *J* = 11.6, 3.9 Hz, 1H, OC*H*H), 2.41 (s, 3H, SC*H*_3_), 1.65 (ddd, *J* = 9.2, 4.4, 1.5 Hz, 1H, 5C*H*), 1.45 (s, 3H, C(C*H*_3_)_2_), 1.15 (s, 3H, C(C*H*_3_)_2_), 0.98 (t, *J* = 4.9 Hz, 1H, 6C*H*H), 0.88 (ddd, *J* = 9.1, 5.1, 1.5 Hz, 1H, 6CH*H*); the ^1^H-NMR spectrum displayed small impurities in the range of about 5%. ^13^C-NMR (101 MHz, DMSO-*d*_6_) δ (ppm) = 163.6 (1C, *C*-2*_purine_*), 153.3 (1C, *C*-6*_purine_*), 150.9 (1C, *C*-4*_purine_*), 137.8 (1C, *C*-1*_benzyl_*), 137.6 (1C, *C*-8*_purine_*), 128.5 (4C, *C*3, 5*_benzyl_*), 127.4 (4C, *C*2, 6*_benzyl_*), 127.1 (2C, *C*-4*_benzyl_*), 116.6 (1C, *C*-5*_purine_*), 111.2 (1C, *C*(CH_3_)_2_), 88.3 (1C, *C*-3), 80.9 (1C, *C*-2), 62.6 (1C, O*C*H_2_), 58.1 (1C, *C*4), 50.9 (1C, *C*H_2_ *_benzyl_*), 48.9 (1C, *C*H_2_ *_benzyl_*), 38.7 (1C, *C*-1), 29.6 (1C, *C*5), 25.8 (1C, C(*C*H_3_)_2_), 24.2 (1C, C(*C*H_3_)_2_), 13.8 (1C, S*C*H_3_), 12.6 (1C, *C*-6); the ^13^C-NMR spectrum displayed small impurities in the range of about 5%. FT-IR (neat) *ṽ* (cm^−1^) = 3372 (O-H), 2982, 2924 (C-H_aliphat_.), 1562 (C = C_aromat_.), 1057, 1030 (CO), 748, 733, 698 (CH_aromat_., out of plane).

*(1S,2R,3S,4R,5S)-4-[6-(Dibenzylamino)-2-methylthio-9H-purin-9-yl]-1-(chloromethyl)-2,3-O-isopropylidenebicyclo[3.1.0]hexane-2,3-diol* (**29**). Cyanuric chloride (0.051 g, 0.28 mmol, 1.5 eq.) was stirred with DMF (0.08 mL, 1.04 mmol, 5.8 eq.) for 2 h at rt. Then CH_2_Cl_2_ (1 mL) and the alcohol **28** (0.098 g, 0.18 mmol) were added and the mixture was stirred overnight. Water was added and the phases were separated. The organic phase was washed with K_2_CO_3_ solution, 0.05 M HCl, and water. The organic phase was dried over anh. Na_2_SO_4_, and filtered and concentrated in vacuo. The residue was purified by fc (cyclohexane:ethyl acetate = 7:1, Ø = 2 cm, l = 20 cm, V = 10 mL) to afford the chloride **29** as a colorless solid (R_f_ = 0.35, cyclohexane:ethyl acetate = 5:1), yield 0.066 g (65%). C_30_H_32_ClN_5_O_2_S (562.13 g/mol). Melting point: 182.4 °C. Purity (HPLC: method B): > 99% (t_R_ = 21.54 min). Exact mass (LC-MS-ESI): *m*/*z* calculated for C_30_H_33_ClN_5_O_2_S [M + H]^+^ 562.2038, found 562.2036. ^1^H-NMR (600 MHz, CDCl_3_) δ (ppm) = 7.86 (s, 1H, 8-C*H_purine_*), 7.34–7.29 (m, 4H, 3, 5C*H_benzyl_*), 7.29–7.24 (m, 6H, 2, 4, 6-C*H_benzyl_*), 5.50 (s, 2H, C*H_2 benzyl_*), 5.38 (dd, *J* = 7.2, 1.5 Hz, 1H, 2C*H*), 5.01 (s, 1H, 4-C*H*), 4.95 (s, 2H, C*H_2 benzyl_*), 4.69 (dd, *J* = 7.2, 1.4 Hz, 1H, 3-C*H*), 3.94 (d, *J* = 11.6 Hz, 1H, ClC*H*H), 3.81 (d, *J* = 11.6 Hz, 1H, ClCH*H*), 2.50 (s, 3H, SC*H*_3_), 1.75 (ddd, *J* = 9.4, 4.7, 1.5 Hz, 1H, 5C*H*), 1.56 (s, 3H, C(C*H*_3_)_2_), 1.43 (s, 0.1H, C*H*_2_, solvent: cyclohexane), 1.35 (dd, *J* = 5.9, 4.8 Hz, 1H, 6C*H*H), 1.27 (s, 3H, C(C*H*_3_)_2_), 1.08 (ddd, *J* = 9.4, 5.9, 1.6 Hz, 1H, 6CH*H*). ^13^C-NMR (151 MHz, CDCl_3_) δ (ppm) = 165.0 (1C, *C*-2*_purine_*), 154.2 (1C, *C*-6*_purine_*), 151.6 (1C, *C*4*_purine_*), 137.9 (1C, *C*-1*_benzyl_*), 136.5 (1C, *C*-8*_purine_*), 128.7 (4C, *C*3, 5*_benzyl_*), 128.0 (4C, *C*2, 6*_benzyl_*), 127.5 (2C, *C*4*_benzyl_*), 117.5 (1C, *C*-5*_purine_*), 112.6 (1C, *C*(CH_3_)_2_), 89.4 (1C, *C*-3), 82.4 (1C, *C*2), 59.6 (1C, *C*-4), 51.2 (1C, *C*H_2_ *_benzyl_*), 49.2 (1C, Cl*C*H_2_), 49.0 (1C, *C*H_2_ *_benzyl_*), 39.0 (1C, *C*-1), 33.0 (1C, *C*-5), 26.2 (1C, C(*C*H_3_)_2_), 24.4 (1C, C(*C*H_3_)_2_), 16.4 (1C, *C*-6), 14.8 (1C, S*C*H_3_). FT-IR (neat) *ṽ* (cm^−1^) = 2978, 2928 (C-H_aliphat_.), 1589, 1566 (C = C_aromat_.), 1072, 1049 (CO), 798 (C-Cl), 733, 694 (CH_aromat_., out of plane).

*(1R,2R,3S,4R,5S)-4-[6-(Dibenzylamino)-2-methylthio-9H-purin-9-yl]-1-(hydroxymethyl)bicyclo[3.1.0]hexane-2,3-diol* (**30**). Compound **28** (0.080 g, 0.15 mmol) was dissolved in CH_3_OH (2.5 mL), trifluoroacetic acid (0.32 mL) and H_2_O (0.32 mL) were added. The mixture was heated to 70 °C for 2 h. The solvent was evaporated and the residue was purified by fc (CH_3_CN:H_2_O = 5:95 ⭢ 100:0, 12 mL/min, Biotage^®®^ SNAP Ultra C18, 12 g, V = 20 mL) to afford the alcohol **111** as a colorless solid (R_f_ = 0.32, ethyl acetate = 100%), yield 0.036 g (48%). C_27_H_29_N_5_O_3_S (503.62 g/mol). Purity (HPLC: method B): 98% (t_R_ = 14.50 min). Exact mass (APCI): *m*/*z* calculated for C_27_H_30_N_5_O_3_S [M + H]^+^ 492.2064, found 492.2066. ^1^H-NMR (600 MHz, DMSO-*d*_6_) δ (ppm) = 8.35 (s, 1H, 8-C*H_purine_*), 7.95 (s, 0.1H, C*H*, solvent: DMF), 7.31 (t, *J* = 7.5 Hz, 4H, 3, 5-C*H_benzyl_*), 7.29–7.23 (m, 6H, 2, 4, 6-C*H_benzyl_*), 5.61 (d, *J* = 15.8 Hz, 1H, C*H*H*_benzyl_*), 5.41 (d, *J* = 15.8 Hz, 1H, CH*H_benzyl_*), 5.19 (s, 1H, 3-O*H_l_*), 4.97 (t, *J* = 5.1 Hz, 1H, CH_2_O*H*), 4.92 (d, *J* = 14.7 Hz, 1H, C*H*H*_benzyl_*), 4.78 (d, *J* = 14.7 Hz, 1H, CH*H_benzyl_*), 4.75 (s, 1H, 4-C*H*), 4.57 (ddd, *J* = 8.1, 6.5, 1.6 Hz, 1H, 2C*H*), 4.49 (d, 7.9 Hz, 1H, 2-O*H*), 4.05 (dd, *J* = 11.3, 5.3 Hz, 1H, OC*H*H), 3.72 (ddt, *J* = 6.4, 4.7, 1.3 Hz, 1H, 3-C*H*), 3.13 (dd, *J* = 11.4, 4.8 Hz, 1H, OCH*H*), 2.89 (s, 0.4H, C*H*_3_, solvent: DMF), 2.73 (s, 0.3H, C*H*_3_, solvent: DMF), 2.41 (s, 3H, SC*H*_3_), 2.07 (s, 0.1H, C*H*_3_CN, solvent: acetonitrile), 1.45 (ddd, *J* = 8.8, 3.9, 1.4 Hz, 1H, 5C*H*), 1.34 (t, *J* = 4.3 Hz, 1H, 6C*H*H), 0.60 (ddd, *J* = 8.6, 4.7, 1.7 Hz, 1H, 6CH*H*); the ^1^HNMR spectrum displayed small impurities in the range of about 5%. ^13^C-NMR (151 MHz, DMSO-*d*_6_) δ (ppm) = 163.4 (1C, *C*-2*_purine_*), 162.3 (0.1C, *C*H, solvent: DMF), 153.3 (1C, *C*6*_purine_*), 151.1 (1C, *C*-4*_purine_*), 137.8 (2C, *C*-1*_benzyl_*), 137.4 (1C, *C*-8*_purine_*), 128.5 (4C, *C*3, 5*_benzyl_*), 127.4 (4C, *C*-2, 6*_benzyl_*), 127.1 (2C, *C*-4*_benzyl_*), 116.5 (1C, *C*-5*_purine_*), 76.0 (1C, *C*-3), 70.2 (1C, *C*2), 62.2 (1C, O*C*H_2_), 60.6 (1C, *C*-4), 50.8 (1C, *C*H_2_ *_benzyl_*), 48.9 (1C, *C*H_2_ *_benzyl_*), 36.4 (1C, *C*-1), 35.8 (0.1C, *C*H_3_, solvent: DMF), 30.8 (0.1C, *C*H_3_, solvent: DMF), 23.2 (1C, *C*5), 13.8 (1C, S*C*H_3_), 11.1 (1C, *C*6). FT-IR (neat) *ṽ* (cm^−1^) = 3368 (O-H), 2978, 2924 (C-H_aliphat_.), 1562 (C = C_aromat_.), 1069 (CO), 733, 698 (CH_aromat_., out of plane).

*(1S,2R,3S,4R,5S)-4-[6-(Dibenzylamino)-2-methylthio-9H-purin-9-yl]-1-(chloromethyl)bicyclo[3.1.0]hexane-2,3-diol* (**31**). Compound **29** (0.055 g, 0.10 mmol) was dissolved in a mixture of CH_3_OH (1.6 mL) and CH_2_Cl_2_ (1.5 mL). Trifluoroacetic acid (0.20 mL) and H_2_O (0.20 mL) were added. The mixture was heated to 70 °C for 6 h. The solvent was evaporated and the residue was purified by fc (CH_3_CN:H_2_O = 5:95 ⭢ 100:0, 12 mL/min, Biotage^®®^ SNAP Ultra C18, 12 g, V = 20 mL) to afford the product **31** as a colorless solid (R_f_ = 0.31, cyclohexane:ethyl acetate = 1:1), yield 0.042 g (82%). C_27_H_28_ClN_5_O_2_S (522.06 g/mol). Purity (HPLC: method B): 97% (t_R_ = 17.55 min). Exact mass (LC-MS-ESI): *m*/*z* calculated for C_27_H_29_ClN_5_O_2_S [M + H]^+^ 522.1725, found 522.1713. ^1^H-NMR (600 MHz, DMSO-*d*_6_) δ (ppm) = 8.09 (s, 1H, 8-C*H_purine_*), 7.32 (t, *J* = 7.5 Hz, 4H, 3, 5-C*H_benzyl_*), 7.27 (d, *J* = 7.4 Hz, 6H, 2, 4, 6-C*H_benzyl_*), 5.54 (d, *J* = 15.2 Hz, 1H, C*H*H*_benzyl_*), 5.48 (d, *J* = 15.4 Hz, 1H, CH*H_benzyl_*), 5.30 (s, 1H, 3-O*H*), 4.87 (d, *J* = 14.9 Hz, 1H, C*H*H*_benzyl_*), 4.82 (d, *J* = 14.9 Hz, 1H, CH*H_benzyl_*), 4.79 (t, *J* = 7.5 Hz, 1H, 2-O*H*), 4.70 (s, 1H, 4-C*H*), 4.62 (ddd, *J* = 7.8, 6.7, 1.6 Hz, 1H, 2-C*H*), 4.16 (d, *J* = 11.4 Hz, 1H, ClC*H*H), 4.03 (q, *J* = 7.1 Hz, 0.2H, C*H*_2_, solvent: ethyl acetate), 3.94 (ddt, *J* = 6.4, 4.7, 1.5 Hz, 1H, 3-C*H*), 3.74 (d, *J* = 11.4 Hz, 1H, ClCH*H*), 2.41 (s, 3H, SC*H*_3_), 1.99 (s, 0.3H, OC*H*_3_, solvent: ethyl acetate), 1.69 (ddd, *J* = 9.2, 4.1, 1.2 Hz, 1H, 5-C*H*), 1.53 (t, *J* = 4.5 Hz, 1H, 6-C*H*H), 1.17 (t, *J* = 7.1 Hz, 0.1H, CH_2_C*H*_3_, solvent: ethyl acetate), 0.88 (ddd, *J* = 8.7, 4.8, 1.7 Hz, 1H, 6-CH*H*). ^13^C-NMR (151 MHz, DMSO-*d*_6_) δ (ppm) = 163.5 (1C, *C*-2*_purine_*), 153.4 (1C, *C*-6*_purine_*), 151.2 (1C, *C*-4*_purine_*), 137.9 (1C, *C*-1*_benzyl_*), 137.6 (1C, *C*-1*_benzyl_*), 137.0 (1C, *C*-8*_purine_*), 128.5 (4C, *C*-3, 5*_benzyl_*), 127.4 (4C, *C*-2, 6*_benzyl_*), 127.1 (2C, *C*-4*_purine_*), 116.5 (1C, *C*-5*_purine_*), 76.1 (1C, *C*-3), 70.9 (1C, *C*-2), 61.0 (1C, *C*-4), 59.8 (0.1C, *C*H_2_, solvent: ethyl acetate), 50.8 (1C, *C*H_2_ *_benzyl_*), 49.4 (1C, Cl*C*H_2_), 48.8 (1C, *C*H_2_ *_benzyl_*), 35.9 (1C, *C*-1), 25.5 (1C, *C*-5), 20.8 (0.1C, O*C*H_3_, solvent: ethyl acetate), 14.8 (1C, *C*-6), 14.1 (0.1C, CH_2_*C*H_3_, solvent: ethyl acetate), 13.8 (1C, S*C*H_3_). FT-IR (neat) *ṽ* (cm^−1^) = 3341 (O-H), 2978 (C-H_aliphat_.), 1562 (C = C_aromat_.), 1072 (C-O), 783 (C-Cl), 733, 694 (C-H_aromat_., out of plane).

*Di-(tert-butyl)-N-[9-((1R,2R,3S,4R,5S)-1-{[(tert-butyldiphenylsilyl)oxy]methyl}-2,3-dihydroxy-2,3-O-isopropylidenebicyclo[3.1.0]hex-4-yl)-2-chloro 9H-purin-6-yl]dicarbamate* (**35**). An amount of 2-chloroadenine (3.02 g, 17.8 mmol) was suspended in THF (88 mL) and di-*tert*-butyl dicarbonate (15.9 g, 73.1 mmol, 4.1 eq.) and DMAP (0.22 g, 1.82 mmol, 0.1 eq.) were added. The mixture was stirred at rt overnight. The solvent was evaporated and the residue redissolved in ethyl acetate. The organic phase was washed with 1 M HCl and brine. After drying over anh. Na_2_SO_4_, the solvent was evaporated. The residue was dissolved in CH_3_OH (177 mL) and saturated NaHCO_3_ solution (80 mL) was added. The mixture was stirred for 2.5 h at 50 °C. CH_3_OH was evaporated and the aqueous residue diluted with H_2_O. The aqueous phase was extracted four times with CH_2_Cl_2_ After drying over anh. Na_2_SO_4_, the solvent was evaporated. The residue was purified by fc (cyclohexane:ethyl acetate = 1:4, Ø = 6 cm, l = 10 cm, V = 65 mL), but only a mixture of product and byproducts were obtained. It was purified again by fc (cyclohexane:ethyl acetate = 1:1, Ø = 6 cm, l = 10 cm, V = 65 mL) to afford the pure product as a colorless solid (R_f_ = 0.17, cyclohexane:ethyl acetate = 1:1), yield 4.85 g (74%). C_15_H_20_ClN_5_O_4_ (369.81 g/mol). Melting point: 85.9 °C. Purity (HPLC: method B): 99% (t_R_ = 13.25 min). Exact mass (APCI): *m*/*z* calculated for C_15_H_21_ClN_5_O_4_ [M + H]^+^ 370.1277, found 370.1277. ^1^H-NMR (400 MHz, DMSO-*d*_6_) δ (ppm) = 13.60 (s, 1H, N*H*), 8.60 (s, 1H, 8-C*H_purine_*), 1.41 (s, 18H, C(C*H*_3_)_3_). ^13^C-NMR (101 MHz, DMSO-*d*_6_) δ (ppm) = 150.7 (1C, *C*-2*_purine_*), 149.0 (2C, *C* = O), 146.5 (1C, *C*-8*_purine_*), 83.5 (2C, *C*(CH_3_)_3_), 27.0 (6C, C(*C*H_3_)_3_); *C*-1, *C*-3 and *C*-5 were not visible. FT-IR (neat) *ṽ* (cm^−1^) = 3244 (N-H), 2978 (C-H_aliphat_.), 1778, 1736 (C = O), 1134, 1107 (C-O).

Next, the purine (1.14 g, 3.08 mmol, 1.1 eq.) and triphenylphospane (1.05 g, 4.00 mmol, 1.5 eq.) were dissolved in THF (25 mL) under nitrogen atmosphere. DIAD (0.78 mL, 3.97 mmol, 1.5 eq.) was added dropwise at 0 °C. The mixture was stirred for 30 min at rt. A solution of the alcohol **4** (1.19 g, 2.70 mmol) in THF (22 mL) was added and the solution was stirred overnight. The solvent was evaporated and the residue was purified by fc (cyclohexane:ethyl acetate = 5:1 + 0.5% triethylamine, Ø = 6 cm, l = 10 cm, V = 65 mL) to afford the product **35** as a colorless solid (R_f_ = 0.32, cyclohexane:ethyl acetate = 5:1), yield 1.80 g (84%). C_41_H_52_ClN_5_O_7_Si (790.43 g/mol). Melting point: 88.6 °C. Purity (HPLC: method C): 98% (t_R_ = 18.48 min). Exact mass (APCI): *m*/*z* calculated for C_31_H_37_ClN_5_O_3_Si [M + H^+^, -2 COOC(CH_3_)_3_, +2H]^+^ 590.2349, found 590.2362. ^1^H-NMR (400 MHz, DMSO-*d*_6_) δ (ppm) = 8.74 (s, 1H, 8-C*H_purine_*), 7.59 (ddd, *J* = 7.9, 6.4, 1.5 Hz, 4H, 2, 6-C*H_Ph_*), 7.47–7.31 (m, 6H, 3, 4, 5-C*H_Ph_*), 5.23 (dd, *J* = 7.1, 1.2 Hz, 1H, 2-C*H_bicyclohexane_*), 5.04 (s, 1H, 4-C*H_bicyclohexane_*), 4.83 (dd, *J* = 7.2, 1.6 Hz, 1H, 3-C*H_bicyclohexane_*), 4.06 (d, *J* = 10.6 Hz, 1H, OC*H*H), 3.83 (d, *J* = 10.6 Hz, 1H, OCH*H*), 1.72 (ddd, *J* = 9.2, 4.5, 1.5 Hz, 1H, 5-C*H_bicyclohexane_*), 1.46 (s, 3H, C(C*H*_3_)_2_), 1.41 (s, 18H, OC(C*H*_3_)_3_), 1.39 (s, 0.4H, C*H*_2_, solvent: cyclohexane), 1.20 (s, 3H, C(C*H*_3_)_2_), 0.99 (s, 11H, 6-C*H_2 bicyclohexane_*, SiC(C*H*_3_)_3_). ^13^C-NMR (101 MHz, DMSO-*d*_6_) δ (ppm) = 153.8 (1C, *C*-4*_purine_*), 151.1 (1C, *C*-2*_purine_*), 149.9 (1C, *C*-6*_purine_*), 149.6 (2C, *C* = O), 144.8 (1C, *C*-8*_purine_*), 135.0 (4C, *C*-2, 6*_Ph_*), 132.7 (2C, *C*-1*_Ph_*), 129.8 (2C, *C*-4*_Ph_*), 127.8 (4C, *C*-3, 5*_Ph_*), 126.9 (1C, *C*-5*_purine_*), 111.5 (1C, *C*(CH_3_)_2_), 87.6 (1C, *C*-3*_bicyclohexane_*), 84.1 (2C, O*C*(CH_3_)_3_), 81.4 (1C, *C*-2*_bicyclohexane_*), 64.3 (1C, O*C*H_2_), 59.4 (1C, *C*-4*_bicyclohexane_*), 38.3 (1C, *C*-1*_bicyclohexane_*), 29.4 (1C, *C*-5*_bicyclohexane_*), 27.2 (6C, OC(*C*H_3_)_3_), 26.7 (3C, SiC(*C*H_3_)_3_), 26.3 (0.1C, *C*H_2_, solvent: cyclohexane), 25.8 (1C, C(*C*H_3_)_2_), 24.3 (1C, C(*C*H_3_)_2_), 18.8 (1C, Si*C*(CH_3_)_3_), 11.9 (1C, *C*-6*_bicyclohexane_*). FT-IR (neat) *ṽ* (cm^−1^) = 2978, 2932 (C-H_aliphat_.), 1759 (C = O), 1593, 1574 (C = C_aromat_.), 1107, 1069, 1038 (C-O), 741, 702 (C-H_aromat_., out of plane).

*Tert-Butyl-N-{9-[(1R,2R,3S,4R,5S)-2,3-dihydroxy-1-(hydroxymethyl)-2,3-O-isopropylidenebicyclo[3.1.0]hex-4-yl]-2-methylthio-9H-purin-6-ylcarbamate* (**37**). Compound **35** (1.00 g, 1.27 mmol) was dissolved in THF (20 mL). Tetrabutylammonium fluoride trihydrate (TBAF x 3H_2_O, 0.60 g, 1.91 mmol, 1.5 eq.) was added and the mixture was stirred at rt for 1 h. The solvent was evaporated, and the residue was dissolved in DMF (20 mL). NaSCH_3_ (1.35 g, 19.3 mmol, 15 eq.) was added, and the slurry was stirred overnight. Next, H_2_O (0.5 mL) was added, and the mixture was heated to 70 °C for 2 h. The reaction was concentrated in vacuo and the residue was purified by fc (CH_3_CN:H_2_O = 5:95 ⭢ 100:0, 50 mL/min, Biotage^®®^ SNAP C18, 120 g, V = 20 mL) to afford the alcohol **37** as a colorless solid (R_f_ = 0.24, ethyl acetate = 100%), yield 0.37 g (63%). C_21_H_29_N_5_O_5_S (463.55 g/mol). Melting point: 110.1 °C. Purity (HPLC: method B): 94% (t_R_ = 12.27 min). Exact mass (LC-MS-ESI): *m*/*z* calculated for C_21_H_30_N_5_O_5_S [M + H]^+^ 464.1962, found 464.1966. ^1^H-NMR (600 MHz, DMSO-*d*_6_) δ (ppm) = 10.90 (s, 1H, N*H*), 8.42 (s, 1H, 8-C*H_purine_*), 5.75 (s, 0.6H, C*H*_2_Cl_2_, solvent: dichloromethane), 5.23 (dd, *J* = 7.1, 1.3 Hz, 1H, 2C*H_bicyclohexane_*), 4.99 (s, 1H, O*H*), 4.95 (s, 1H, 4-C*H_bicyclohexane_*), 4.65 (dd, *J* = 7.1, 1.5 Hz, 1H, 3-C*H_bicyclohexane_*), 3.84 (dd, *J* = 11.5, 4.0 Hz, 1H, OC*H*H), 3.37 (dd, *J* = 11.5, 3.9 Hz, 1H, OCH*H*), 2.59 (s, 3H, SC*H*_3_), 1.64 (ddd, *J* = 9.2, 4.4, 1.5 Hz, 1H, 5C*H_bicyclohexane_*), 1.48 (s, 9H, C(C*H*_3_)_3_), 1.45 (s, 3H, C(C*H*_3_)_2_), 1.18 (s, 3H, C(C*H*_3_)_2_), 0.98 (t, *J* = 4.8 Hz, 1H, 6C*H*H*_bicyclohexane_*), 0.89 (ddd, *J* = 9.1, 5.1, 1.5 Hz, 1H, 6CH*H_bicyclohexane_*); the ^1^HNMR spectrum displayed small impurities in the range of about 5%. ^13^C-NMR (151 MHz, DMSO-*d*_6_) δ (ppm) = 163.8 (1C, *C*-2*_purine_*), 151.9 (1C, *C*-4*_purine_*), 151.0 (1C, *C* = O), 149.7 (1C, *C*-6*_purine_*), 140.9 (1C, *C*-8*_purine_*), 120.6 (1C, *C*-5*_purine_*), 111.3 (1C, *C*(CH_3_)_2_), 88.1 (1C, *C*-3*_bicyclohexane_*), 80.9 (1C, *C*-2*_bicyclohexane_*), 80.2 (1C, *C*(CH_3_)_3_), 62.5 (1C, O*C*H_2_), 58.4 (1C, *C*-4*_bicyclohexane_*), 54.9 (0.3C, *C*H_2_Cl_2_, solvent: dichloromethane), 38.8 (1C, *C*-1*_bicyclohexane_*), 29.6 (1C, *C*5*_bicyclohexane_*), 27.9 (3C, C(*C*H_3_)_3_), 25.6 (1C, C(*C*H_3_)_2_), 24.2 (1C, C(*C*H_3_)_2_), 14.0 (1C, S*C*H_3_), 12.6 (1C, *C*6*_bicyclohexane_*); the ^13^C-NMR spectrum displayed small impurities in the range of about 5%. FT-IR (neat) *ṽ* (cm^−1^) = 3341 (O-H), 2978 (C-H_aliphat_.), 1759 (C = O), 1609, 1582 (C = C_aromat_.), 1134, 1061, 1015 (C-O).

*Tert-Butyl-N-{9-[(1R,2R,3S,4R,5S)-1-(azidomethyl)-2,3-dihydroxy-2,3-O-isopropylidenebicyclo[3.1.0]hex-4-yl]-2-methylthio-9H-purin-6-yl}carbamate* (**38**). The alcohol **37** (2.19 g, 4.72 mmol) was suspended in CH_2_Cl_2_ (110 mL), tosyl chloride (1.81 g, 9.49 mmol, 2 eq.), triethylamine (1.5 mL, 10.8 mmol, 2.3 eq.), and DMAP (0.066 g, 0.54 mmol, 0.1 eq.) were added. The mixture was stirred at rt overnight. Water was added and the mixture was extracted four times with CH_2_Cl_2_. The organic phase was dried over anh. Na_2_SO_4_ and concentrated in vacuo. The residue was dissolved in DMF (60 mL) and NaN_3_ (4.60 g, 70.8 mmol, 15 eq.) was added. The mixture was heated to 70 °C for 2 h. Water and brine were added and the reaction mixture was extracted four times with CH_2_Cl_2_. The organic phase was dried over anh. Na_2_SO_4_ and concentrated in vacuo. The residue was purified by fc (CH_3_CN:H_2_O = 5:95 ⭢ 80:20, 50 mL/min, Biotage^®®^ SNAP C18, 120 g, V = 20 mL) to afford the azide **38** as a colorless solid (R_f_ = 0.27, cyclohexane:ethyl acetate = 1:1), yield 1.26 g (55%). C_21_H_28_N_8_O_4_S (488.57 g/mol). Melting point: 83.8 °C. Purity (HPLC: method B): 98% (t_R_ = 16.55 min). Exact mass (APCI): *m*/*z* calculated for C_31_H_37_ClN_5_O_3_Si [M + H]^+^ 489.2027, found 489.2027. ^1^H-NMR (400 MHz, DMSO-*d*_6_) δ (ppm) = 10.08 (s, 1H, N*H*), 8.31 (s, 1H, 8-C*H_purine_*), 5.75 (s, 0.2H, C*H*_2_Cl_2_, solvent: dichloromethane), 5.25 (dd, *J* = 7.1, 1.3 Hz, 1H, 2C*H_bicyclohexane_*), 4.97 (s, 1H, 4-C*H_bicyclohexane_*), 4.81 (dd, *J* = 7.1, 1.3 Hz, 1H, 3C*H_bicyclohexane_*), 3.74 (d, *J* = 13.0 Hz, 1H, NC*H*H), 3.47 (d, *J* = 13.0 Hz, 1H, NCH*H*), 2.60 (s, 3H, SC*H*_3_), 2.08 (s, 0.1H, C*H*_3_CN, solvent: acetonitrile), 1.71 (ddd, *J* = 9.3, 4.6, 1.5 Hz, 1H, 5C*H_bicyclohexane_*), 1.48 (s, 9H, C(C*H*_3_)_3_), 1.47 (s, 3H, C(C*H*_3_)_2_), 1.20 (s, 3H, C(C*H*_3_)_2_), 1.09 (t, *J* = 5.0 Hz, 1H, 6C*H*H*_bicyclohexane_*), 1.04 (ddd, *J* = 9.2, 5.3, 1.5 Hz, 1H, 6-CH*H_bicyclohexane_*). ^13^C-NMR (101 MHz, DMSO-*d*_6_) δ (ppm) = 163.7 (1C, *C*-2*_purine_*), 152.0 (1C, *C*-4*_purine_*), 150.8 (1C, *C* = O), 149.6 (1C, *C*-6*_purine_*), 141.5 (1C, *C*-8*_purine_*), 120.8 (1C, *C*-5*_purine_*), 111.5 (1C, *C*(CH_3_)_2_), 88.1 (1C, *C*-3*_bicyclohexane_*), 82.8 (1C, *C*-2*_bicyclohexane_*), 80.2 (1C, *C*(CH_3_)_3_), 59.0 (1C, *C*-4*_bicyclohexane_*), 54.1 (1C, N*C*H_2_), 36.3 (1C, *C*-1*_bicyclohexane_*), 30.0 (1C, *C*5*_bicyclohexane_*), 27.8 (3C, C(*C*H_3_)_3_), 25.8 (1C, C(*C*H_3_)_2_), 24.1 (1C, C(*C*H_3_)_2_), 14.0 (1C, S*C*H_3_), 13.9 (1C, *C*-6*_bicyclohexane_*). FT-IR (neat) *ṽ* (cm^−1^) = 2982, 2924 (C-H_aliphat_.), 2099 (N = N=N), 1751, 1712 (C = O), 1605, 1578 (C = C_aromat_.), 1138, 1053 (C-O).

*(1R,2R,3S,4R,5S)-4-(6-Amino-2-methylthio-9H-purin-9-yl)-1-{[4-(hydroxymethyl)-1H-1,2,3-triazol-1-yl]methyl}bicyclo[3.1.0]hexane-2,3-diol* (**39**). The azide **38** (0.030 g, 0.06 mmol) was dissolved in *tert*-butanol (0.5 mL). Propargyl alcohol (0.015 mL, 0.26 mmol, 4.2 eq.), copper(II) acetylacetonate (0.001 g, 0.004 mmol, 0.06 eq.), sodium ascorbate (0.007 g, 0.04 mmol, 0.6 eq.), and H_2_O (0.5 mL) were added. The mixture was stirred for 5 h at rt. The solvent was evaporated and the residue was purified by fc (CH_3_CN:H_2_O = 5:95 ⭢ 100:0, 12 mL/min, Biotage^®®^ SNAP Ultra C18, 12 g, V = 20 mL) to afford the Boc-protected triazole as a colorless solid (R_f_ = 0.30, CH_2_Cl_2_:CH_3_OH = 95:5), yield 0.020 g (59%). C_24_H_32_N_8_O_5_S (544.63 g/mol). Purity (HPLC: method B): 97% (t_R_ = 11.30 min). Exact mass (LC-MS-ESI): *m*/*z* calculated for C_24_H_32_DN_8_O_5_S [M + H]^+^ 546.2352, found 546.2337. Exact mass (APCI): *m*/*z* calculated for C_19_H_25_N_8_O_3_S [M + 2H^+^, -COOC(CH_3_)_3_]^+^ 445.1765, found 445.1765. ^1^H-NMR (400 MHz, CD_3_OD) δ (ppm) = 7.97 (s, 1H, 5-C*H_triazole_*), 5.34 (dd, *J* = 7.1, 1.4 Hz, 1H, 2-C*H_bicyclohexane_*), 5.00 (s, 1H, 4-C*H_bicyclohexane_*), 4.95 (d, *J* = 14.6 Hz, 1H, NC*H*H), 4.87 (dd, *J* = 7.3, 1.5 Hz, 1H, 3-C*H_bicyclohexane_*), 4.69 (dd, *J* = 13.2, 2.1 Hz, 2H, OC*H*_2_), 4.52 (d, *J* = 14.6 Hz, 1H, NCH*H*), 3.35 (s, 0.8H, C*H*_3_OH, solvent: methanol), 2.63 (s, 3H, SC*H*_3_), 1.89 (ddd, *J* = 9.4, 4.7, 1.6 Hz, 1H, 5C*H_bicyclohexane_*), 1.58 (s, 9H, C(C*H*_3_)_3_), 1.47 (s, 3H, C(C*H*_3_)_2_), 1.26 (t, *J* = 5.2 Hz, 1H, 6-C*H*H*_bicyclohexane_*), 1.22 (s, 3H, C(C*H*_3_)_2_), 1.15 (ddd, *J* = 9.4, 5.7, 1.5 Hz, 1H, 6-CH*H_bicyclohexane_*); 8-C*H_purine_* was not visible due to occurrence of deuterium exchange at this position. ^13^C-NMR (101 MHz, CD_3_OD) δ (ppm) = 167.3 (1C, *C*-2*_purine_*), 153.3 (1C, *C*-4*_purine_*), 152.5 (1C, *C* = O), 150.9 (1C, *C*-6*_purine_*), 149.2 (1C, *C*-4*_triazole_*), 124.4 (1C, *C*-5*_triazole_*), 120.8 (1C, *C*-5*_purine_*), 113.6 (1C, *C*(CH_3_)_2_), 90.2 (1C, *C*-3*_bicyclohexane_*), 84.6 (1C, *C*2*_bicyclohexane_*), 82.7 (1C, *C*(CH_3_)_3_), 61.7 (1C, *C*-4*_bicyclohexane_*), 56.6 (1C, O*C*H_2_), 54.8 (1C, N*C*H_2_), 38.2 (1C, *C*-1*_bicyclohexane_*), 33.4 (1C, *C*5*_bicyclohexane_*), 28.5 (3C, C(*C*H_3_)_3_), 26.2 (1C, C(*C*H_3_)_2_), 24.4 (1C, C(*C*H_3_)_2_), 15.2 (1C, *C*6*_bicyclohexane_*), 14.8 (1C, S*C*H_3_); *C*8*_purine_* was not visible. FT-IR (neat) *ṽ* (cm^−1^) = 3341 (O-H), 3148 (N-H), 2978 (C-H_aliphat_.), 1751, 1717 (C = O), 1605, 1578 (C = C_aromat_.), 1142, 1053 (C-O).

The triazole (0.015 g, 0.03 mmol) was dissolved in CH_3_OH (0.8 mL) and trifluoroacetic acid (0.10 mL) and H_2_O (0.10 mL) were added. The mixture was heated to 70 °C for 6 h. The solvent was evaporated and the residue was purified by semi-preparative HPLC (method B) to afford the product **39** as a colorless solid (R_f_ = 0.30, CH_2_Cl_2_:CH_3_OH = 8:2), yield 0.006 g (54%). C_16_H_20_N_8_O_3_S (404.14 g/mol). Purity (HPLC: method B): 97% (t_R_ = 3.55 min). Exact mass (LC-MS-ESI): *m*/*z* calculated for C_16_H_20_DN_8_O_3_S [M + H]^+^ 406.1515, found 406.1515 and for C_16_H_21_N_8_O_3_S [M + H]^+^ 405.1452, found 405.1454. ^1^H-NMR (600 MHz, DMSO-*d*_6_) δ (ppm) = 8.00 (s, 1H, 5-C*H_triazole_*), 7.42 (s, 0.3H, 8C*H_purine_*), 7.32 (s, 1H, N*H*_2_), 4.73 (d, *J* = 14.5 Hz, 1H, NC*H*H), 4.61 (d, *J* = 1.8 Hz, 1H, 4-C*H*), 4.55–4.49 (m, 3H, NCH*H*, OC*H*_2_), 4.52 (dd, *J* = 6.6, 1.4 Hz, 1H, 2-C*H*), 4.03 (q, *J* = 7.1 Hz, 0.1H, C*H*_2_, solvent: ethyl acetate),3.81 (dt, *J* = 6.5, 1.6 Hz, 1H, 3-C*H*), 2.47 (s, 3H, SC*H*_3_), 1.99 (s, 0.1H, OC*H*_3_, solvent: ethyl acetate), 1.67 (dd, *J* = 8.5, 4.0 Hz, 1H, 5C*H*), 1.42 (t, *J* = 4.5 Hz, 1H, 6-C*H*H), 1.17 (t, *J* = 7.1 Hz, 0.1H, CH_2_C*H*_3_, solvent: ethyl acetate), 0.83 (ddd, *J* = 8.7, 4.9, 1.6 Hz, 1H, 6-CH*H*); 8-C*H_purine_* showed a reduced intensity due to occurrence of deuterium exchange at this position. ^13^C-NMR (151 MHz, DMSO-*d*_6_) δ (ppm) = 163.8 (1C, *C*-2*_purine_*), 155.4 (1C, *C*-6*_Purine_*), 149.7 (1C, *C*-4*_purine_*), 148.0 (1C, *C*-4*_triazole_*), 137.4 (1C, *C*-8*_purine_*), 123.4 (1C, *C*-5*_triazole_*), 116.4 (1C, *C*-5*_purine_*), 76.5 (1C, *C*-3), 71.8 (1C, *C*-2), 61.1 (1C, *C*-4), 55.1 (1C, O*C*H_2_) 52.2 (1C, N*C*H_2_), 34.7 (1C, *C*1), 24.3 (1C, *C*-5), 13.7 (1C, S*C*H_3_), 12.8 (1C, *C*-6).

*Methyl 1-{[(1R,2R,3S,4R,5S)-4-(6-amino-2-methylthio-9H-purin-9-yl)-2,3-dihydroxybicyclo[3.1.0]hex-1-yl]methyl}-1H-1,2,3-triazole-4-carboxylate* (**40**). The azide **38** (0.054 g, 0.11 mmol) was dissolved in *tert*-butanol (0.8 mL) and methyl propiolate (0.045 mL, 0.51 mmol, 4.6 eq.), copper(II) acetylacetonate (0.004 g, 0.02 mmol, 0.1 eq.), sodium ascorbate (0.010 g, 0.05 mmol, 0.5 eq.), and H_2_O (0.8 mL) were added. The mixture was stirred at 80° C for 1.5 h. The solvent was evaporated and the residue was purified by fc (CH_3_CN:H_2_O = 5:95 ⭢ 100:0, 12 mL/min, Biotage^®®^ SNAP Ultra C18, 12 g, V = 20 mL) to afford the triazole **125** as a colorless solid (R_f_ = 0.34, ethyl acetate = 100%), yield 0.042 g (66%). C_25_H_32_N_8_O_6_S (572.64 g/mol). Purity (HPLC: method B): 91% (t_R_ = 14.38 min). Exact mass (APCI): *m*/*z* calculated for C_20_H_25_N_8_O_4_S [M + 2H, -COOC(CH_3_)_3_]^+^ 473.1714, found 473.1709. ^1^H-NMR (600 MHz, DMSO-*d*_6_) δ (ppm) = 10.11 (s, 1H, N*H*), 8.77 (s, 1H, 5-C*H*), 8.18 (s, 1H, 8C*H_purine_*), 5.75 (s, 0.2H, C*H*_2_Cl_2_, solvent: dichloromethane), 5.24 (dd, *J* = 7.1, 1.4 Hz, 1H, 2C*H_bicyclohexane_*), 4.98 (s, 1H, 4-C*H_bicyclohexane_*), 4.97 (d, *J* = 14.5 Hz, 1H, NC*H*H), 4.83 (dd, *J* = 7.3, 1.5 Hz, 1H, 3-C*H_bicyclohexane_*), 4.49 (d, *J* = 14.5 Hz, 1H, NCH*H*), 3.84 (s, 3H, OC*H*_3_), 2.57 (s, 3H, SC*H*_3_), 2.01 (ddd, *J* = 9.4, 4.7, 1.6 Hz, 1H, 5C*H_bicyclohexane_*), 1.48 (s, 9H, C(C*H*_3_)_3_), 1.39 (s, 3H, C(C*H*_3_)_2_), 1.25 (ddd, *J* = 9.2, 5.4, 1.6 Hz, 1H, 6C*H*H*_bicyclohexane_*), 1.14 (s, 3H, C(C*H*_3_)_2_), 1.08 (t, *J* = 5.0 Hz, 1H, 6CH*H_bicyclohexane_*); the ^1^HNMR spectrum displayed small impurities in the range of about 5%. ^13^C-NMR (151 MHz, DMSO-*d*_6_) δ (ppm) = 163.8 (1C, *C*-2*_purine_*), 160.7 (1C, *C*-4*_carbonyl_*), 152.0 (1C, *C*-4*_purine_*), 150.8 (1C, *C*-N*_carbonyl_*), 149.6 (1C, *C*-6*_purine_*), 141.4 (1C, *C*-8*_purine_*), 138.7 (1C, *C*4), 129.1 (1C, *C*-5), 120.7 (1C, *C*-5*_purine_*), 111.5 (1C, *C*(CH_3_)_2_), 88.2 (1C, *C*-3*_bicyclohexane_*), 82.4 (1C, *C*-2*_bicyclohexane_*), 80.2 (1C, *C*(CH_3_)_3_), 59.1 (1C, *C*-4*_bicyclohexane_*), 54.9 (0.1C, *C*H_2_Cl_2_, solvent: dichloromethane), 53.3 (1C, N*C*H_2_), 51.8 (1C, O*C*H_3_), 36.7 (1C, *C*-1*_bicyclohexane_*), 31.8 (1C, *C*5*_bicyclohexane_*), 27.9 (3C, C(*C*H_3_)_3_), 25.8 (1C, C(*C*H_3_)_2_), 24.1 (1C, C(*C*H_3_)_2_), 14.1 (1C, *C*6*_bicyclohexane_*), 14.0 (1C, S*C*H_3_); the ^13^CNMR spectrum displayed small impurities in the range of about 5%. FT-IR (neat) *ṽ* (cm^−1^) = 2978 (C-H_aliphat_.), 1732 (C = O), 1605, 1578 (C = C_aromat_.), 1142, 1069, 1053 (C-O).

The triazole (0.038 g, 0.07 mmol) was dissolved in CH_3_OH (1.6 mL), trifluoroacetic acid (0.20 mL) and H_2_O (0.20 mL) were added. The mixture was heated to 70 °C overnight. The solvent was evaporated and the residue was purified by fc (CH_3_CN:H_2_O = 5:95 ⭢ 100:0, 12 mL/min, Biotage^®®^ SNAP C18, 12 g, V = 20 mL) to afford the product **40** as a colorless solid (R_f_ = 0.32, CH_2_Cl_2_:CH_3_OH = 9:1), yield 0.013 g (44%). C_17_H_20_N_8_O_4_S (432.46 g/mol). Purity (HPLC: method B): 96% (t_R_ = 4.86 min). Exact mass (LC-MS-ESI): *m*/*z* calculated for C_17_H_21_N_8_O_4_S [M + H]^+^ 433.1401, found 433.1402. ^1^H-NMR (600 MHz, DMSO-*d*_6_) δ (ppm) = 8.76 (s, 1H, 5-C*H*), 7.55 (s, 1H, 8-C*H_purine_*), 7.31 (s, 2H, N*H*_2_), 5.26 (d, *J* = 4.8 Hz, 1H, 3-O*H_bicyclohexane_*), 4.76–4.72 (m, 2H, 2-O*H_bicyclohexane_*, NC*H*H), 4.69 (d, *J* = 14.5 Hz, 1H, NCH*H*), 4.63 (s, 1H, 4-C*H_bicyclohexane_*), 4.52 (td, *J* = 7.3, 1.5 Hz, 1H, 2-C*H_bicyclohexane_*), 4.09 (q, *J* = 5.3 Hz, 0.4H, C*H*_3_OH, solvent: methanol), 3.90 (ddt, *J* = 6.5, 4.8, 1.6 Hz, 1H, 3-C*H_bicyclohexane_*), 3.83 (s, 3H, OC*H*_3_), 3.17 (d, *J* = 5.2 Hz, 0.8H, C*H*_3_OH, solvent: methanol), 2.45 (s, 3H, SC*H*_3_), 1.77 (dd, *J* = 8.5, 4.0 Hz, 1H, 5-C*H_bicyclohexane_*), 1.43 (t, *J* = 4.5 Hz, 1H, 6-C*H*H*_bicyclohexane_*), 0.91 (ddd, *J* = 8.7, 4.9, 1.6 Hz, 1H, 6-CH*H_bicyclohexane_*). ^13^C-NMR (151 MHz, DMSO-*d*_6_) δ (ppm) = 163.9 (1C, *C*-2*_purine_*), 160.7 (1C, *C* = O), 155.4 (1C, *C*-6*_purine_*), 149.7 (1C, *C*-4*_purine_*), 138.5 (1C, *C*-4), 137.6 (1C, *C*-8*_purine_*), 129.4 (1C, *C*-5), 116.6 (1C, *C*-5*_purine_*), 76.3 (1C, *C*-3*_bicyclohexane_*), 72.2 (1C, *C*-2*_bicyclohexane_*), 61.4 (1C, *C*-4*_bicyclohexane_*), 53.2 (1C, N*C*H_2_), 51.7 (1C, O*C*H_3_), 48.6 (0.2C, *C*H_3_OH, solvent: methanol), 34.4 (1C, *C*-1*_bicyclohexane_*), 24.8 (1C, *C*-5*_bicyclohexane_*), 13.7 (1C, S*C*H_3_), 12.9 (1C, *C*-6*_bicyclohexane_*). FT-IR (neat) *ṽ* (cm^−1^) = 3341 (O-H), 3148 (N-H), 2978 (C-H_aliphat_.), 1678 (C = O), 1589 (C = C_aromat_.), 1130, 1080, 1053 (C-O).

*Methyl 2-(1-{[(1R,2R,3S,4R,5S)-4-(6-amino-2-methylthio-9H-purin-9-yl)-2,3-dihydroxybicyclo[3.1.0]hex-1-yl]methyl}-1H-1,2,3-triazol-4-yl)acetate* (**41**). The azide **38** (0.045 g, 0.09 mmol) was dissolved in *tert*-butanol (0.75 mL) and 3-butynoic acid (0.031 g, 0.37 mmol, 4 eq.), copper(II) acetylacetonate (0.001 g, 0.004 mmol, 0.04 eq.), sodium ascorbate (0.007 g, 0.04 mmol, 0.4 eq.), and H_2_O (0.75 mL) were added. The mixture was stirred at rt for 6 h. The solvent was evaporated and the residue was purified by fc (CH_3_CN:H_2_O = 5:95 ⭢ 100:0, 12 mL/min, Biotage^®®^ SNAP Ultra C18, 12 g, V = 20 mL) to afford the triazole as a colorless solid (R_f_ = 0.18, CH_2_Cl_2_:CH_3_OH = 9:1), yield 0.017 g (33%). C_25_H_32_N_8_O_6_S (572.64 g/mol). Purity (HPLC: method B): 84% (t_R_ = 12.93 min). Exact mass (LC-MS-ESI): *m*/*z* calculated for C_25_H_33_N_8_O_6_S [M + H]^+^ 573.2238, found 573.2223. ^1^H-NMR (600 MHz, DMSO-*d*_6_) δ (ppm) = 10.11 (s, 1H, N*H*), 8.14 (s, 1H, 8-C*H_purine_*), 8.00 (s, 1H, 5-C*H_triazole_*), 5.19 (s, 1H, 2-C*H_bicyclohexane_*), 4.97 (s, 1H, 4-C*H_bicyclohexane_*), 4.83 (d, *J* = 13.7 Hz, 1H, NC*H*H), 4.79 (d, *J* = 7.0 Hz, 1H, 3-C*H_bicyclohexane_*), 4.50 (d, *J* = 14.5 Hz, 1H, NCH*H*), 3.66 (s, 2H, 2-C*H*_2_), 3.17 (s, 0.1H, C*H*_3_OH, solvent: methanol), 2.58 (s, 3H, SC*H*_3_), 1.90 (s, 1H, 5-C*H_bicyclohexane_*), 1.49 (s, 9H, C(C*H*_3_)_3_), 1.42 (s, 3H, C(C*H*_3_)_2_), 1.15 (s, 4H, C(C*H*_3_)_2_, 6-C*H*H*_bicyclohexane_*), 1.09 (s, 1H, 6-CH*H_bicyclohexane_*). ^13^C-NMR (151 MHz, DMSO-*d*_6_) δ (ppm) = 171.5 (1C, *C*-1), 163.8 (1C, *C*-2*_purine_*), 152.0 (1C, *C*-4*_purine_*), 150.8 (1C, *C*-N*_carbonyl_*), 149.6 (1C, *C*-6*_purine_*), 141.3 (1C, *C*-8*_purine_*), 140.5 (1C, *C*-4*_triazole_*), 123.7 (1C, *C*-5*_triazole_*), 120.5 (1C, *C*-5*_purine_*), 111.5 (1C, *C*(CH_3_)_2_), 88.3 (1C, *C*-3*_bicyclohexane_*), 82.3 (1C, *C*-2*_bicyclohexane_*), 80.3 (1C, *C*(CH_3_)_3_), 58.9 (1C, *C*-4*_bicyclohexane_*), 52.5 (1C, N*C*H_2_), 36.6 (1C, *C*-1*_bicyclohexane_*), 31.7 (1C, *C*-5*_bicyclohexane_*), 31.6 (1C, *C*-2), 27.9 (3C, C(*C*H_3_)_3_), 25.8 (1C, C(*C*H_3_)_2_), 24.1 (1C, C(*C*H_3_)_2_), 14.0 (2C, *C*-6*_bicyclohexane_*, S*C*H_3_). FT-IR (neat) *ṽ* (cm^−1^) = 2986 (C-H_aliphat_.), 1721 (C = O), 1605, 1578 (C = C_aromat_.), 1142, 1053 (C-O).

The triazole (0.065 g, 0.11 mmol) was dissolved in CH_3_OH (1.7 mL) and trifluoroacetic acid (0.20 mL) and H_2_O (0.10 mL) were added. The mixture was heated to 60 °C for 1 d and then at rt overnight. The solvent was evaporated and the residue was purified by semi-preparative HPLC (method F) to afford the ester **41** as a colorless solid (R_f_ = 0.24, CH_2_Cl_2_:CH_3_OH = 9:1), yield 0.024 g (48%). C_18_H_22_N_8_O_4_S (446.15 g/mol). Melting point: 214.6 °C. Purity (HPLC: method B): 98% (t_R_ = 5.15 min). Exact mass (LC-MS-ESI): *m*/*z* calculated for C_18_H_23_N_8_O_4_S [M + H]^+^ 447.1557, found 447.1558. The compound **41** shows two different rotamers a and b in the NMR spectra in a ratio of approximately 10:1. ^1^H-NMR (400 MHz, DMSO-*d*_6_) δ (ppm) = 8.58 (s, 0.1H, 5-C*H_triazole, rotam_. _b_*), 8.04 (s, 1H, 5-C*H_triazole, rotam_. _a_*), 7.72 (s, 0.1H, 8-C*H_purine, rotam_. _b_*), 7.42 (s, 1H, 8-C*H_purine, rotam_. _a_*), 7.30 (s, 2.1H, N*H_2, rotam_. _a, b_*), 5.25 (d, *J* = 4.3 Hz, 1H, 3a-O*H_bicyclohexane_*), 4.90 (d, *J* = 14.4 Hz, 0.1H, NC*H*H*_rotam_. _b_*), 4.82 (d, *J* = 7.3 Hz, 0.1H, 2-C*H_bicyclohexane, rotam_. _b_*), 4.81–4.72 (m, 2H, 2-O*H_bicyclohexane, rotam_. _a_*, NC*H*H*_rotam_. _a_*), 4.68 (s, 0.1H, 4-C*H_bicyclohexane, rotam_. _b_*), 4.61 (s, 1H, 4-C*H_bicyclohexane, rotam_. _a_*), 4.52 (d, *J* = 14.5 Hz, 1H, NCH*H_rotam_. _a_*), 4.42 (t, *J* = 5.7 Hz, 1.1H, 2-C*H_bicyclohexane, rotam_. _a_*, NCH*H_rotam_. _b_*), 3.97 (dd, *J* = 7.0, 1.7 Hz, 0.1H, 3-C*H_bicyclohexane, rotam_. _b_*), 3.86-3.72 (m, 3H, 3-C*H_bicyclohexane, rotam_. _a_*, 2-C*H_2, rotam_. _a_*), 3.63 (s, 3H, OC*H_3, rotam_. _a_*), 3.58 (s, 0.2H, OC*H_3, rotam_. _b_*), 3.52 (d, *J* = 16.8 Hz, 0.1H, 2-C*H*H*_rotam_. _b_*), 2.47 (s, 2.9H, SC*H_3, rotam_. _a, b_*), 1.85 (t, *J* = 4.7 Hz, 0.1H, 6-C*H*H*_bicyclohexane, rotam_. _b_*), 1.67 (dd, *J* = 8.7, 4.0 Hz, 1H, 5-C*H_bicyclohexane, rotam_. _a_*), 1.60 (dd, *J* = 9.1, 4.6 Hz, 0.1H, 5-C*H_bicyclohexane, rotam_. _b_*), 1.42 (t, *J* = 4.5 Hz, 1H, 6-C*H*H*_bicyclohexane, rotam_. _a_*), 0.83 (ddd, *J* = 8.7, 4.9, 1.5 Hz, 1H, 6-CH*H_bicyclohexane, rotam_. _a_*), 0.67–0.57 (m, 0.1H, 6-CH*H_bicyclohexane, rotam_. _b_*); the signal for 2-CH*H_rotam_. _b_* is located under the H_2_O signal and therefore only visible in 2D spectra. ^13^C-NMR (101 MHz, DMSO-*d*_6_) δ (ppm) = 170.4 (1C, *C* = O), 163.8 (1C, *C*-2*_purine_*), 155.4 (1C, *C*-6*_purine_*), 149.8 (1C, *C*-4*_purine_*), 139.8 (1C, *C*-4*_triazole_*), 137.4 (1C, *C*-8*_purine_*), 124.2 (1C, *C*-5*_triazole_*), 116.5 (1C, *C*-5*_purine_*), 76.5 (1C, *C*-3*_bicyclohexane_*), 71.8 (1C, *C*-2*_bicyclohexane_*), 61.2 (1C, *C*-4*_bicyclohexane_*), 52.2 (1C, N*C*H_2_), 51.9 (1C, O*C*H_3_), 34.7 (1C, *C*-1*_bicyclohexane_*), 31.1 (1C, *C*-2), 24.4 (1C, *C*-5*_bicyclohexane_*), 13.7 (1C, S*C*H_3_), 12.9 (1C, *C*-6*_bicyclohexane_*). The resolution was too low to identify the ^13^C signals for rotamer b, therefore only rotamer a is described here. FT-IR (neat) *ṽ* (cm^−1^) = 3352 (O-H), 3244 (N-H), 1751 (C = O), 1624, 1585 (C = C_aromat_.), 1142, 1119, 1084, 1057 (C-O).

*Methyl 3-(1-{[(1R,2R,3S,4R,5S)-4-(6-amino-2-methylthio-9H-purin-9-yl)-2,3-dihydroxybicyclo[3.1.0]hex-1-yl]methyl}-1H-1,2,3-triazol-4-yl)propanoate* (**42**). The azide **38** (0.028 g, 0.06 mmol) was dissolved in *tert*-butanol (0.5 mL) and 4-pentynoic acid (0.024 g, 0.24 mmol, 4.3 eq.), copper(II) acetylacetonate (0.001 g, 0.004 mmol, 0.07 eq.), sodium ascorbate (0.006 g, 0.03 mmol, 0.5 eq.), and H_2_O (0.5 mL) were added. The mixture was stirred at rt for 5 h. The solvent was evaporated and the residue was purified by fc (CH_3_CN:H_2_O = 5:95 ⭢ 100:0, 12 mL/min, Biotage^®®^ SNAP Ultra C18, 12 g, V = 20 mL) to afford the triazole as a colorless solid (R_f_ = 0.27, CH_2_Cl_2_:CH_3_OH = 9:1), yield 0.018 g (54%). C_26_H_34_N_8_O_6_S (586.67 g/mol). Purity (HPLC: method B): 96% (t_R_ = 12.64 min). Exact mass (LC-MS-ESI): *m*/*z* calculated for C_26_H_34_DN_8_O_6_S [M + H]^+^ 588.2458, found 588.2452. ^1^H-NMR (400 MHz, CD_3_OD) δ (ppm) = 7.99 (s, 0.1H, 8-C*H_purine_*), 7.82 (s, 1H, 5-C*H_triazole_*), 5.23 (dd, *J* = 7.1, 1.4 Hz, 1H, 2-C*H_bicyclohexane_*), 5.00 (s, 1H, 4-C*H_bicyclohexane_*), 4.88–4.82 (m, 1H, 3-C*H_bicyclohexane_*, NC*H*H), 4.52 (d, *J* = 14.6 Hz, 1H, NCH*H*), 3.35 (s, 0.2H, C*H*_3_OH, solvent: methanol), 2.99 (t, *J* = 7.4 Hz, 2H, 3-C*H*_2_), 2.67 (t, *J* = 7.4 Hz, 2H, 2-C*H*_2_), 2.63 (s, 3H, SC*H*_3_), 1.89 (ddd, *J* = 9.4, 4.6, 1.6 Hz, 1H, 5-C*H_bicyclohexane_*), 1.58 (s, 9H, C(C*H*_3_)_3_), 1.48 (s, 3H, C(C*H*_3_)_2_), 1.26 (dd, *J* = 5.7, 4.7 Hz, 1H, 6-C*H*H*_bicyclohexane_*), 1.22 (s, 3H, C(C*H*_3_)_2_), 1.13 (ddd, *J* = 9.4, 5.7, 1.5 Hz, 1H, 6-CH*H_bicyclohexane_*); 8-C*H_purine_* showed a reduced intensity and no coupling interaction due to occurrence of deuterium exchange at this position. ^13^C-NMR (101 MHz, CD_3_OD) δ (ppm) = 176.3 (1C, *C*-1), 167.4 (1C, *C*-2*_purine_*), 153.3 (1C, *C*-4*_purine_*), 152.5 (1C, *C*-N*_carbonyl_*), 150.9 (1C, *C*-6*_purine_*), 147.9 (1C, *C*-4*_triazole_*), 142.6 (1C, *C*-8*_purine_*), 123.9 (1C, *C*-5*_triazole_*), 120.7 (1C, *C*-5*_purine_*), 113.6 (1C, *C*(CH_3_)_2_), 90.0 (1C, *C*-3*_bicyclohexane_*), 84.4 (1C, *C*-2*_bicyclohexane_*), 82.7 (1C, *C*(CH_3_)_3_), 61.6 (1C, *C*-4*_bicyclohexane_*), 54.7 (1C, N*C*H_2_), 38.2 (1C, *C*-1*_bicyclohexane_*), 34.5 (1C, *C*-2), 33.2 (1C, *C*-5*_bicyclohexane_*), 28.5 (3C, C(*C*H_3_)_3_), 26.2 (1C, C(*C*H_3_)_2_), 24.4 (1C, C(*C*H_3_)_2_), 22.0 (1C, *C*-3), 15.3 (1C, *C*-6*_bicyclohexane_*), 14.8 (1C, S*C*H_3_). FT-IR (neat) *ṽ* (cm^−1^) = 2978 (C-H_aliphat_.), 1717 (C = O), 1605, 1578 (C = C_aromat_.), 1142, 1053 (C-O).

The triazole acid (0.015 g, 0.03 mmol) was dissolved in CH_3_OH (0.4 mL) and trifluoroacetic acid (0.05 mL) and H_2_O (0.05 mL) were added. The mixture was stirred at rt for 3 d. The solvent was evaporated and the residue was purified by semi-preparative HPLC (method D) to afford the ester **42** as a colorless solid (R_f_ = 0.24, CH_2_Cl_2_:CH_3_OH = 9:1), yield 0.004 g (34%). C_19_H_24_N_8_O_4_S (460.16 g/mol). Purity (HPLC: method B): 98% (t_R_ = 5.67 min). Exact mass (LC-MS-ESI): *m*/*z* calculated for C_19_H_24_DN_8_O_4_S [M + H]^+^ 462.1777, found 462.1775 and for C_19_H_25_N_8_O_4_S [M + H]^+^ 461.1714, found 461.1709. ^1^H-NMR (600 MHz, DMSO-*d*_6_) δ (ppm) = 7.88 (s, 1H, 5-C*H_triazole_*), 7.30 (s, 0.4H, 8C*H_purine_*), 7.29 (s, 2H, N*H*_2_), 5.24 (d, *J* = 4.7 Hz, 1H, 3-O*H_bicyclohexane_*), 4.76 (d, *J* = 7.3 Hz, 1H, 2O*H_bicyclohexane_*), 4.73 (d, *J* = 14.5 Hz, 1H, NC*H*H), 4.61 (d, *J* = 1.7 Hz, 1H, 4-C*H_bicyclohexane_*), 4.44 (d, *J* = 14.5 Hz, 1H, NCH*H*), 4.40 (td, *J* = 7.0, 1.7 Hz, 1H, 2C*H_bicyclohexane_*), 4.03 (q, *J* = 7.1 Hz, 0.1H, C*H*_2_, solvent: ethyl acetate), 3.81 (tt, *J* = 6.3, 1.6 Hz, 1H, 3-C*H_bicyclohexane_*), 3.58 (s, 3H, OC*H*_3_), 2.88 (t, *J* = 7.5 Hz, 2H, 3-C*H*_2_), 2.67 (t, *J* = 7.5 Hz, 2H, 2-C*H*_2_), 2.46 (s, 3H, SC*H*_3_), 1.99 (s, 0.1H, OC*H*_3_, solvent: ethyl acetate), 1.65 (dd, *J* = 8.3, 4.1 Hz, 1H, 5C*H_bicyclohexane_*), 1.42 (t, *J* = 4.5 Hz, 1H, 6C*H*H*_bicyclohexane_*), 1.17 (t, *J* = 7.1 Hz, 0.1H, CH_2_C*H*_3_, solvent: ethyl acetate), 0.81 (ddd, *J* = 8.7, 4.9, 1.6 Hz, 1H, 6-CH*H_bicyclohexane_*); 8-C*H_purine_* showed a reduced intensity due to occurrence of deuterium exchange at this position. ^13^C-NMR (151 MHz, DMSO-*d*_6_) δ (ppm) = 172.6 (1C, *C*-1), 163.8 (1C, *C*-2*_purine_*), 155.4 (1C, *C*6*_purine_*), 149.7 (1C, *C*-4*_purine_*), 145.4 (1C, *C*-4*_triazole_*), 137.3 (1C, *C*-8*_purine_*), 122.7 (1C, *C*-5*_triazole_*), 116.4 (1C, *C*-5*_purine_*), 76.3 (1C, *C*-3*_bicyclohexane_*), 71.7 (1C, *C*-2*_bicyclohexane_*), 61.1 (1C, *C*-4*_bicyclohexane_*), 52.0 (1C, N*C*H_2_), 51.4 (1C, O*C*H_3_), 34.8 (1C, *C*-1*_bicyclohexane_*), 32.8 (1C, *C*-2), 24.2 (1C, *C*-5*_bicyclohexane_*), 20.7 (1C, *C*-3), 13.6 (1C, S*C*H_3_), 12.9 (1C, *C*6*_bicyclohexane_*).

*(1R,2R,3S,4R,5S)-4-(6-Amino-2-methylthio-9H-purin-9-yl)-1-{[4-(aminomethyl)-1H-1,2,3-triazol-1-yl]methyl}bicyclo[3.1.0]hexane-2,3-diol* (**43**). The azide **38** (0.029 g, 0.06 mmol) was dissolved in *tert*-butanol (0.5 mL) and propargylamine (0.016 mL, 0.25 mmol, 4.2 eq.), copper(II) acetylacetonate (0.001 g, 0.004 mmol, 0.06 eq.), sodium ascorbate (0.007 g, 0.04 mmol, 0.6 eq.), and H_2_O (0.5 mL) were added. The mixture was stirred for 5 h at rt. The solvent was evaporated and the residue was purified by fc (CH_3_CN:H_2_O = 5:95 ⭢ 100:0 + 0.1% trifluoroacetic acid, 12 mL/min, Biotage^®®^ SNAP Ultra C18, 12 g, V = 20 mL). The intermediate already partly decomposed (deprotection) during this purification and was therefore dissolved in CH_3_OH (0.5 mL). Next, triflouroacetic acid (0.05 mL) and H_2_O (0.10 mL) were added and the mixture was heated to 70 °C for 1 d. The solvent was evaporated and the residue was purified by fc (CH_3_CN:H_2_O = 5:95 ⭢ 100:0 + 0.1% trifluoroacetic acid, 12 mL/min, Biotage^®®^ SNAP Ultra C18, 12 g, V = 20 mL), but the product remained impure. The impure product was purified by semi-preparative HPLC (method D) to afford the pure product **43** as a colorless solid (R_f_ = 0.15, CH_3_OH = 100% + 1% triethylamine), yield 0.002 g (8%). C_16_H_21_N_9_O_2_S (403.47 g/mol). Purity (HPLC: method D): 95% (t_R_ = 8.88 min). Exact mass (LC-MS-ESI): *m*/*z* calculated for C_16_H_22_N_9_O_2_S [M + H]^+^ 404.1612, found 404.1624. ^1^H-NMR (600 MHz, DMSO-*d*_6_) δ (ppm) = 7.94 (s, 1H, 5-C*H_triazole_*), 7.30 (s, 2H, N*H*_2_), 7.29 (s, 1H, 8-C*H_purine_*), 4.76 (d, *J* = 14.5 Hz, 1H, NC*H*H), 4.62 (s, 1H, 4-C*H*), 4.46 (d, *J* = 14.4 Hz, 1H, NCH*H*), 4.41 (dd, *J* = 6.6, 1.5 Hz, 1H, 2-C*H*), 3.81 (dt, *J* = 6.6, 1.6 Hz, 1H, 3-C*H*), 3.77 (s, 2H, NH_2_C*H*_2_), 2.46 (s, 3H, SC*H*_3_), 1.66 (dd, *J* = 8.7, 4.0 Hz, 1H, 5C*H*), 1.43 (t, *J* = 4.5 Hz, 1H, 6-C*H*H), 0.83 (ddd, *J* = 8.7, 4.9, 1.6 Hz, 1H, 6-CH*H*). ^13^C-NMR (151 MHz, DMSO-*d*_6_) δ (ppm) = 163.8 (1C, *C*-2*_purine_*), 155.4 (1C, *C*-6*_purine_*), 149.7 (1C, *C*-4*_purine_*), 149.1 (1C, *C*-4*_triazole_*), 137.3 (1C, *C*-8*_purine_*), 122.7 (1C, *C*-5*_triazole_*), 116.5 (1C, *C*-5*_purine_*), 76.3 (1C, *C*-3), 71.7 (1C, *C*-2), 61.1 (1C, *C*-4), 52.0 (1C, N*C*H_2_), 37.1 (1C, NH_2_*C*H_2_), 34.9 (1C, *C*-1), 24.3 (1C, *C*-5), 13.6 (1C, S*C*H_3_), 12.9 (1C, *C*-6).

*1-{[(1R,2R,3S,4R,5S)-4-(6-Amino-2-methylthio-9H-purin-9-yl)-2,3-dihydroxybicyclo[3.1.0]hex-1-yl]methyl}-1H-1,2,3-triazole-4-carboxylic acid* (**44**). The ester **40** (0.082 g, 0.19 mmol) was suspended in CH_3_CN (0.7 mL) and H_2_O (2 mL), and NaOH solution-(2 M, 0.30 mL) was added. The mixture was heated to 60 °C overnight. The solvent was evaporated and the residue was purified by semi preparative HPLC (method A) to afford the carboxylic acid **44** as a colorless solid (R_f_ = 0.44, CH_2_Cl_2_:CH_3_OH = 1:1), yield 0.036 g (45%). C_16_H_18_N_8_O_4_S (418.43 g/mol). Melting point: 139.3 °C. Purity (HPLC: method B): > 99% (t_R_ = 7.05 min). Exact mass (LC-MS-ESI): *m*/*z* calculated for C_16_H_19_N_8_O_4_S [M + H]^+^ 419.1244, found 419.1241. ^1^H-NMR (600 MHz, DMSO-*d*_6_) δ (ppm) = 8.27 (s, 1H, 5-C*H*), 7.35 (s, 2H, N*H*_2_), 7.24 (s, 1H, 8-C*H_purine_*), 5.50 (s, 2H, 2-O*H_bicyclohexane_*, 3-O*H_bicyclohexane_*), 4.71 (d, *J* = 14.6 Hz, 1H, NC*H*H), 4.63 (d, *J* = 2.0 Hz, 1H, 4-C*H_bicyclohexane_*), 4.53 (d, *J* = 14.5 Hz, 1H, NCH*H*), 4.44 (dd, *J* = 6.5, 1.5 Hz, 1H, 2-C*H_bicyclohexane_*), 3.76 (dt, *J* = 6.6, 1.5 Hz, 1H, 3-C*H_bicyclohexane_*), 2.47 (s, 3H, SC*H*_3_), 2.07 (s, 0.1H, C*H*_3_CN, solvent: acetonitrile), 1.67 (dd, *J* = 8.4, 3.7 Hz, 1H, 5-C*H_bicyclohexane_*), 1.46 (t, *J* = 4.4 Hz, 1H, 6-C*H*H*_bicyclohexane_*), 0.85 (ddd, *J* = 8.7, 4.8, 1.6 Hz, 1H, 6-CH*H_bicyclohexane_*). ^13^C-NMR (151 MHz, DMSO-*d*_6_) δ (ppm) = 164.2 (1C, *C* = O), 163.9 (1C, *C*-2*_purine_*), 155.4 (1C, *C*-6*_purine_*), 149.7 (1C, *C*-4*_purine_*), 148.3 (1C, *C*-4), 137.1 (1C, *C*-8*_purine_*), 126.5 (1C, *C*-5), 116.4 (1C, *C*-5*_purine_*), 76.8 (1C, *C*-3*_bicyclohexane_*), 71.7 (1C, *C*-2*_bicyclohexane_*), 60.8 (1C, *C*-4*_bicyclohexane_*), 52.2 (1C, N*C*H_2_), 34.7 (1C, *C*-1*_bicyclohexane_*), 24.1 (1C, *C*-5*_bicyclohexane_*), 13.7 (1C, S*C*H_3_), 13.0 (1C, *C*-6*_bicyclohexane_*). FT-IR (neat) *ṽ* (cm^−1^) = 3341 (O-H), 3198 (N-H), 1585 (C = O), 1539 (C = C_aromat_.), 1057 (C-O).

*2-(1-{[(1R,2R,3S,4R,5S)-4-(6-Amino-2-methylthio-9H-purin-9-yl)-2,3-dihydroxybicyclo[3.1.0]hex-1-yl]methyl}-1H-1,2,3-triazol-4-yl)acetic acid* (**45**). The azide **38** (0.045 g, 0.09 mmol) was dissolved in *tert*-butanol (0.75 mL) and 3-butynoic acid (0.031 g, 0.37 mmol, 4 eq.), copper(II) acetylacetonate (0.001 g, 0.004 mmol, 0.04 eq.), sodium ascorbate (0.007 g, 0.04 mmol, 0.4 eq.), and H_2_O (0.75 mL) were added. The mixture was stirred at rt for 6 h. The solvent was evaporated and the residue was purified by fc (CH_3_CN:H_2_O = 5:95 ⭢ 100:0, 12 mL/min, Biotage^®®^ SNAP Ultra C18, 12 g, V = 20 mL) to afford the triazole as a colorless solid (R_f_ = 0.18, CH_2_Cl_2_:CH_3_OH = 9:1), yield 0.017 g (33%). C_25_H_32_N_8_O_6_S (572.64 g/mol). Purity (HPLC: method B): 84% (t_R_ = 12.93 min). Exact mass (LC-MS-ESI): m/z calculated for C_25_H_33_N_8_O_6_S [M + H]^+^ 573.2238, found 573.2223. ^1^H-NMR (600 MHz, DMSO-*d*_6_) δ (ppm) = 10.11 (s, 1H, N*H*), 8.14 (s, 1H, 8-C*H_purine_*), 8.00 (s, 1H, 5-C*H_triazole_*), 5.19 (s, 1H, 2-C*H_bicyclohexane_*), 4.97 (s, 1H, 4C*H_bicyclohexane_*), 4.83 (d, *J* = 13.7 Hz, 1H, NC*H*H), 4.79 (d, *J* = 7.0 Hz, 1H, 3-C*H_bicyclohexane_*), 4.50 (d, *J* = 14.5 Hz, 1H, NCH*H*), 3.66 (s, 2H, 2-C*H*_2_), 3.17 (s, 0.1H, C*H*_3_OH, solvent: methanol), 2.58 (s, 3H, SC*H*_3_), 1.90 (s, 1H, 5C*H_bicyclohexane_*), 1.49 (s, 9H, C(C*H*_3_)_3_), 1.42 (s, 3H, C(C*H*_3_)_2_), 1.15 (s, 4H, C(C*H*_3_)_2_, 6C*H*H*_bicyclohexane_*), 1.09 (s, 1H, 6CH*H_bicyclohexane_*). ^13^C-NMR (151 MHz, DMSO-*d*_6_) δ (ppm) = 171.5 (1C, *C*-1), 163.8 (1C, *C*-2*_purine_*), 152.0 (1C, *C*4*_purine_*), 150.8 (1C, *C*-N*_carbonyl_*), 149.6 (1C, *C*-6*_purine_*), 141.3 (1C, *C*-8*_purine_*), 140.5 (1C, *C*-4*_triazole_*), 123.7 (1C, *C*-5*_triazole_*), 120.5 (1C, *C*-5*_purine_*), 111.5 (1C, *C*(CH_3_)_2_), 88.3 (1C, *C*-3*_bicyclohexane_*), 82.3 (1C, *C*-2*_bicyclohexane_*), 80.3 (1C, *C*(CH_3_)_3_), 58.9 (1C, *C*4*_bicyclohexane_*), 52.5 (1C, N*C*H_2_), 36.6 (1C, *C*1*_bicyclohexane_*), 31.7 (1C, *C*-5*_bicyclohexane_*), 31.6 (1C, *C*-2), 27.9 (3C, C(*C*H_3_)_3_), 25.8 (1C, C(*C*H_3_)_2_), 24.1 (1C, C(*C*H_3_)_2_), 14.0 (2C, *C*6*_bicyclohexane_*, S*C*H_3_). FT-IR (neat) *ṽ* (cm^−1^) = 2986 (C-H_aliphat_.), 1721 (C = O), 1605, 1578 (C = C_aromat_.), 1142, 1053 (CO).

The triazole (0.048 g, 0.08 mmol) was dissolved in CH_3_CN (0.4 mL) and H_2_O (1.4 mL), and trifluoroacetic acid (0.20 mL) was added. The mixture was heated to 60 °C overnight. The solvent was evaporated and the was purified by semi-preparative HPLC (method E) to afford the product **45** as a colorless solid, yield 0.019 g (51%). C_17_H_20_N_8_O_4_S (432.46 g/mol). Melting point: 179.2 °C. Purity (HPLC: D): 99% (t_R_ = 9.71 min). Exact mass (LC-MS-ESI): *m*/*z* calculated for C_17_H_21_N_8_O_4_S [M + H]^+^ 433.1401, found 433.1411. The compound **45** shows two different rotamers a and b in the NMR spectra in a ratio of approximately 7:1. ^1^H-NMR (400 MHz, DMSO-*d*_6_) δ (ppm) = 8.54 (s, 0.2H, 5-C*H_triazole, rotam_. _b_*), 8.02 (s, 1H, 5-C*H_triazole, rotam_. _a_*), 7.70 (s, 0.1H, 8-C*H_purine, rotam_. _b_*), 7.43 (s, 1H, 8-C*H_purine, rotam_. _a_*), 7.30 (s, 2.4H, N*H_2, rotam_. _a, b_*), 4.86 (d, *J* = 14.4 Hz, 0.2H, NC*H*H*_rotam_. _b_*), 4.81 (d, *J* = 6.5 Hz, 0.2H, 2-C*H_bicyclohexane, rotam_. _b_*), 4.75 (d, 1H, *J* = 14.5 Hz, NC*H*H*_rotam_. _a_*), 4.68 (s, 0.2H, 4-C*H_bicyclohexane, rotam_. _b_*), 4.61 (s, 1H, 4-C*H_bicyclohexane, rotam_. _a_*), 4.52 (d, *J* = 14.5 Hz, 1H, NCH*H_rotam_. _a_*), 4.47 (s, 0.2H, NCH*H_rotam_. _b_*), 4.42 (d, *J* = 6.6 Hz, 1H, 2-C*H_bicyclohexane, rotam_. _a_*), 3.95 (d, *J* = 6.7 Hz, 0.2H, 3-C*H_bicyclohexane, rotam_. _b_*), 3.81 (d, 1H, *J* = 6.4 Hz, 3-C*H_bicyclohexane, rotam_. _a_*) 3.66 (t, *J* = 19.2 Hz, 2H, 2-C*H_2, rotam_. _a_*), 3.43 (d, *J* = 16.7 Hz, 0.1H, 2-C*H*H*_rotam_. _b_*), 3.18 (d, *J* = 16.9 Hz, 0.1H, 2-CH*H_rotam_. _b_*), 2.47 (s, 3.5H, SC*H_3, rotam_. _a, b_*), 1.85 (s, 0.2H, 6-C*H*H*_bicyclohexane, rotam_. _b_*), 1.66 (dd, *J* = 8.9, 3.9 Hz, 1H, 5-C*H_bicyclohexane, rotam_. _a_*), 1.59 (s, 0.2H, 5-C*H_bicyclohexane, rotam_. _b_*), 1.41 (t, *J* = 4.5 Hz, 1H, 6-C*H*H*_bicyclohexane, rotam_. _a_*), 0.82 (dd, *J* = 9.0, 4.9 Hz, 1H, 6-CH*H_bicyclohexane, rotam_. _a_*), 0.63 (s, 0.2H, 6-CH*H_bicyclohexane, rotam_. _b_*). ^13^C-NMR (101 MHz, DMSO-*d*_6_) δ (ppm) = 171.6 (1C, *C* = O), 163.8 (1C, *C*-2*_purine_*), 155.4 (1C, *C*-6*_purine_*), 149.8 (1C, *C*-4*_purine_*), 140.6 (1C, *C*-4*_triazole_*), 137.4 (1C, *C*-8*_purine_*), 124.1 (1C, *C*-5*_triazole_*), 116.5 (1C, *C*-5*_purine_*), 76.6 (1C, *C*-3*_bicyclohexane_*), 71.8 (1C, *C*-2*_bicyclohexane_*), 61.1 (1C, *C*-4*_bicyclohexane_*), 52.2 (1C, N*C*H_2_), 34.7 (1C, *C*-1*_bicyclohexane_*), 31.7 (1C, *C*-2), 24.4 (1C, *C*-5*_bicyclohexane_*), 13.7 (1C, S*C*H_3_), 12.9 (1C, *C*-6*_bicyclohexane_*); the resolution was too low to identify the ^13^C signals for rotamer b, therefore only rotamer a is described here. FT-IR (neat) *ṽ* (cm^−1^) = 3518, 3329, 3198 (O-H), 2920 (C-H_aliphat_.), 1651 (C = O), 1586 (C = C_aromat_.), 1123, 1092 (C-O).

*3-[1-({(1R,2R,3S,4R,5S)-4-[6-(tert-Butoxycarbonyl)amino-2-methylthio-9H-purin-9-yl]-2,3-dihydroxy-2,3-O-isopropylidenebicyclo[3.1.0]hex-1-yl}methyl)-1H-1,2,3-triazol-4-yl]propanoic acid* (**46**). The azide **38** (0.028 g, 0.06 mmol) was dissolved in *tert*-butanol (0.5 mL) and 4p-entynoic acid (0.024 g, 0.24 mmol, 4.3 eq.), copper(II) acetylacetonate (0.001 g, 0.004 mmol, 0.07 eq.), sodium ascorbate (0.006 g, 0.03 mmol, 0.5 eq.), and H_2_O (0.5 mL) were added. The mixture was stirred at rt for 5 h. The solvent was evaporated and the residue was purified by fc (CH_3_CN:H_2_O = 5:95 ⭢ 100:0, 12 mL/min, Biotage^®®^ SNAP Ultra C18, 12 g, V = 20 mL) to afford the triazole as a colorless solid (R_f_ = 0.27, CH_2_Cl_2_:CH_3_OH = 9:1), yield 0.018 g (54%). C_26_H_34_N_8_O_6_S (586.67 g/mol). Purity (HPLC: method B): 96% (t_R_ = 12.64 min). Exact mass (LC-MS-ESI): *m*/*z* calculated for C_26_H_34_DN_8_O_6_S [M + H]^+^ 588.2458, found 588.2452. ^1^H-NMR (400 MHz, CD_3_OD) δ (ppm) = 7.99 (s, 0.1H, 8-C*H_purine_*), 7.82 (s, 1H, 5-C*H_triazole_*), 5.23 (dd, *J* = 7.1, 1.4 Hz, 1H, 2-C*H_bicyclohexane_*), 5.00 (s, 1H, 4-C*H_bicyclohexane_*), 4.88–4.82 (m, 1H, 3-C*H_bicyclohexane_*, NC*H*H), 4.52 (d, *J* = 14.6 Hz, 1H, NCH*H*), 3.35 (s, 0.2H, C*H*_3_OH, solvent: methanol), 2.99 (t, *J* = 7.4 Hz, 2H, 3-C*H*_2_), 2.67 (t, *J* = 7.4 Hz, 2H, 2-C*H*_2_), 2.63 (s, 3H, SC*H*_3_), 1.89 (ddd, *J* = 9.4, 4.6, 1.6 Hz, 1H, 5-C*H_bicyclohexane_*), 1.58 (s, 9H, C(C*H*_3_)_3_), 1.48 (s, 3H, C(C*H*_3_)_2_), 1.26 (dd, *J* = 5.7, 4.7 Hz, 1H, 6-C*H*H*_bicyclohexane_*), 1.22 (s, 3H, C(C*H*_3_)_2_), 1.13 (ddd, *J* = 9.4, 5.7, 1.5 Hz, 1H, 6-CH*H_bicyclohexane_*); 8-C*H_purine_* showed a reduced intensity and no coupling interaction due to occurrence of deuterium exchange at this position. ^13^C-NMR (101 MHz, CD_3_OD) δ (ppm) = 176.3 (1C, *C*-1), 167.4 (1C, *C*-2*_purine_*), 153.3 (1C, *C*-4*_purine_*), 152.5 (1C, *C*-N*_carbonyl_*), 150.9 (1C, *C*-6*_purine_*), 147.9 (1C, *C*-4*_triazole_*), 142.6 (1C, *C*-8*_purine_*), 123.9 (1C, *C*-5*_triazole_*), 120.7 (1C, *C*-5*_purine_*), 113.6 (1C, *C*(CH_3_)_2_), 90.0 (1C, *C*-3*_bicyclohexane_*), 84.4 (1C, *C*-2*_bicyclohexane_*), 82.7 (1C, *C*(CH_3_)_3_), 61.6 (1C, *C*-4*_bicyclohexane_*), 54.7 (1C, N*C*H_2_), 38.2 (1C, *C*-1*_bicyclohexane_*), 34.5 (1C, *C*-2), 33.2 (1C, *C*-5*_bicyclohexane_*), 28.5 (3C, C(*C*H_3_)_3_), 26.2 (1C, C(*C*H_3_)_2_), 24.4 (1C, C(*C*H_3_)_2_), 22.0 (1C, *C*-3), 15.3 (1C, *C*-6*_bicyclohexane_*), 14.8 (1C, S*C*H_3_). FT-IR (neat) *ṽ* (cm^−1^) = 2978 (C-H_aliphat_.), 1717 (C = O), 1605, 1578 (C = C_aromat_.), 1142, 1053 (C-O).

The triazole (0.070 g, 0.12 mmol) was dissolved in CH_3_CN (0.7 mL) and H_2_O (2 mL), and trifluoroacetic acid (0.30 mL) was added. The mixture was stirred at rt for 1 d. The solvent was evaporated and the residue was purified by semi-preparative HPLC (method E) to afford the carboxylic acid **46** as a colorless solid, yield 0.010 g (19%). C_18_H_22_N_8_O_4_S (446.49 g/mol). Melting point: 133.3 °C Purity (HPLC: D): 99% (t_R_ = 10.32 min). Exact mass (LC-MS-ESI): m/z calculated for C_18_H_23_N_8_O_4_S [M + H]^+^ 447.1557, found 447.1557. The compound **46** shows three different rotamers a, b and c in the NMR spectra in a ratio of approximately 10:6:3. ^1^H-NMR (600 MHz, DMSO-*d*_6_) δ (ppm) = 8.40 (s, 0.6H, 5-C*H_triazole, rotam_. _b_*), 7.97 (s, 0.3H, 5-C*H_triazole, rotam_. _c_*), 7.89 (s, 1H, 5-C*H_triazole, rotam_. _a_*), 7.58 (s, 0.6H, 8-C*H_purine, rotam_. _b_*), 7.36 (s, 0.3H, 8-C*H_purine, rotam_. _c_*), 7.30 (s, 2.5H, 8-C*H_purine, rotam_. _a_*, N*H_2, rotam_. _b, c_*), 7.26 (s, 2H, N*H_2, rotam_. _a_*), 5.24 (s, 0.6H, 3-O*H_bicyclohexane_*), 4.80-4.75 (m, 1.3H, 2-C*H_bicyclohexane, rotam_. _b_*, NC*H*H*_rotam_. _b_*), 4.73 (d, *J* = 14.5 Hz, 1H, NC*H*H*_rotam_. _a_*), 4.69 (s, 0.3H, 4-C*H_bicyclohexane, rotam_. _c_*), 4.67 (s, 0.6H, 4-C*H_bicyclohexane, rotam_. _b_*), 4.64 (d, *J* = 7.1 Hz, 0.3H, 2-C*H_bicyclohexane, rotam_. _c_*), 4.62 (d, *J* = 1.7 Hz, 1H, 4-C*H_bicyclohexane, rotam_. _a_*), 4.57 (d, *J* = 14.5 Hz, 0.3H, NC*H*H*_rotam_. _c_*), 4.49–4.42 (m, 1.8H, NCH*H_rotam_. _a, b, c_*), 4.41 (dd, *J* = 6.7, 1.5 Hz, 1H, 2-C*H_bicyclohexane, rotam_. _a_*), 3.98 (d, *J* = 7.0 Hz, 0.2H, 3-C*H_bicyclohexane, rotam_. _c_*), 3.92 (dd, *J* = 6.9, 1.7 Hz, 0.6H, 3-C*H_bicyclohexane, rotam_. _b_*), 3.79 (dt, *J* = 6.6, 1.6 Hz, 1H, 3-C*H_bicyclohexane, rotam_. _a_*), 2.85 (t, *J* = 7.6 Hz, 2H, 3-C*H_2, rotam_. _a_*), 2.80 (t, *J* = 7.6 Hz, 0.6H, 3-C*H_2, rotam_. _c_*), 2.66 (t, *J* = 7.6 Hz, 1.2H, 3-C*H_2, rotam_. _b_*), 2.58 (dd, *J* = 8.3, 6.9 Hz, 2H, 2-C*H_2, rotam_. _a_*), 2.53 (t, *J* = 7.0 Hz, 0.6H, 2-C*H_2, rotam_. _c_*), 2.48 (s, 1.2H, 2-C*H_2, rotam_. _b_*), 2.46 (s, 4.5H, SC*H_3, rotam_. _a, b, c_*), 2.07 (s, 0.1H, C*H*_3_CN, solvent: acetonitrile), 1.85 (t, *J* = 4.6 Hz, 0.6H, 6-C*H*H*_bicyclohexane, rotam_. _b_*), 1.68–1.62 (m, 1.3H, 5-C*H_bicyclohexane, rotam_. _a_*, 6-C*H*H*_bicyclohexane, rotam_. _c_*), 1.58 (ddd, *J* = 9.1, 4.5, 1.7 Hz, 0.6H, 5-C*H_bicyclohexane, rotam_. _b_*), 1.44 (dd, *J* = 4.6, 3.6 Hz, 0.3H, 5-C*H_bicyclohexane, rotam_. _c_*), 1.42 (t, *J* = 4.5 Hz, 1H, 6-C*H*H*_bicyclohexane, rotam_. _a_*), 0.81 (ddd, *J* = 8.7, 4.9, 1.6 Hz, 1H, 6-CH*H_bicyclohexane, rotam_. _a_*), 0.68 (ddd, *J* = 8.5, 3.0, 1.6 Hz, 0.6H, 6-CH*H_bicyclohexane, rotam_. _b_*), 0.60 (dd, *J* = 9.4, 4.7 Hz, 0.3H, 6-CH*H_bicyclohexane, rotam_. _c_*); 3-O*H_bicyclohexane_* could not be clearly assigned to one of the three rotamers. ^13^C-NMR (151 MHz, DMSO-*d*_6_) δ (ppm) = 173.7 (0.6C, *C*-1*_rotam_. _a, c_*), 173.7 (0.3C, *C*-1*_rotam_. _b_*), 163.8 (1C, *C*-2*_purine, rotam_. _a_*), 163.7 (0.3C, *C*-2*_purine, rotam_. _b_*), 163.6 (0.2C, *C*-2*_purine, rotam_. _c_*), 155.4 (1C, *C*-6*_purine, rotam_. _a_*), 155.4 (0.5C, *C*-6*_purine, rotam_. _b, c_*), 149.7 (1C, *C*-4*_purine, rotam_. _a_*), 149.6 (0.5C, *C*-4*_purine, rotam_. _b c_*), 145.8 (0.6C, *C*-4*_triazole, rotam_. _a_*), 145.4 (0.1C, *C*-4*_triazole, rotam_. _c_*), 145.1 (0.4C, *C*-4*_triazole, rotam_. _b_*), 137.8 (0.3C, *C*-8*_purine, rotam_. _b_*), 137.6 (0.1C, *C*-8*_purine, rotam_. _c_*), 137.3 (1C, *C*-8*_purine, rotam_. _a_*), 122.9 (0.4C, *C*-5*_triazole, rotam_. _b_*), 122.8 (0.2C, *C*-5*_triazole, rotam_. _c_*), 122.7 (1C, *C*-5*_triazole, rotam_. _a_*), 116.5 (1C, *C*-5*_purine, rotam_. _a_*), 116.4 (0.4C, *C*-5*_purine, rotam_. _b_*), 116.4 (0.1C, *C*-5*_purine, rotam_. _c_*), 86.4 (0.4C, *C*-3*_bicyclohexane, rotam_. _b_*), 86.1 (0.1C, *C*-3*_bicyclohexane, rotam_. _c_*), 79.7 (0.5C, *C*-2*_bicyclohexane, rotam_. _b_*), 78.6 (0.1C, *C*-2*_bicyclohexane, rotam_. _c_*), 76.3 (1C, *C*-3*_bicyclohexane, rotam_. _a_*), 71.7 (1C, *C*-2*_bicyclohexane, rotam_. _a_*), 61.1 (1C, *C*-4*_bicyclohexane, rotam_. _a_*), 61.0 (0.2C, *C*-4*_bicyclohexane, rotam_. _c_*), 60.7 (0.5C, *C*-4*_bicyclohexane, rotam_. _b_*), 54.4 (0.4C, N*C*H_2,_*_rotam_. _b_*), 53.2 (0.2C, N*C*H_2,_ *_rotam_. _c_*), 52.0 (1C, N*C*H_2,_ *_rotam_. _a_*), 37.4 (0.1C, *C*-1*_bicyclohexane, rotam_. _c_*), 36.4 (0.4C, *C*-1*_bicyclohexane, rotam_. _b_*), 34.8 (1C, *C*-1*_bicyclohexane, rotam_. _a_*), 33.4 (0.9C, *C*-2*_rotam_. _b, c_*), 33.3 (1C, *C*-2*_rotam_. _a_*), 30.1 (0.5C, b-*C*-5*_bicyclohexane, rotam_. _b_*), 29.1 (0.2C, *C*-5*_bicyclohexane, rotam_. _c_*), 24.2 (1C, *C*-5*_bicyclohexane, rotam_. _a_*), 20.8 (0.3C, *C*-3*_rotam_. _c_*), 20.8 (1C, *C*-3*_rotam_. _a_*), 20.7 (0.6C, *C*-3*_rotam_. _b_*), 13.7 (1C, S*C*H_3,_ *_rotam_. _a, c_*), 13.6 (0.4C, S*C*H_3,_*_rotam_. _b_*), 12.9 (1C, *C*-6*_bicyclohexane, rotam_. _a_*), 12.7 (0.2C, *C*-6*_bicyclohexane, rotam_. _c_*), 12.5 (1C, *C*-6*_bicyclohexane, rotam_. _b_*). FT-IR (neat) *ṽ* (cm^−1^) = 3098 (N-H), 2943 (C-H_aliphat_.), 1713, 1670 (C = O), 1539 (C = C_aromat_.), 1192, 1142 (C-O).

*Triethylammonium 2-{[(1R,2R,3S,4R,5S)-4-(6-Amino-2-methylthio-9H-purin-9-yl)-2,3-dihydroxybicyclo[3.1.0]hex-1-yl]methyl}amino-3,4-dioxocyclobut-1-en-1-olate* (**47**). The azide **38** (0.032 g, 0.07 mmol) was dissolved in CH_3_OH (2 mL) and Pd/C (10 wt%, 0.003 g, 0.003 mmol, 0.004 eq.) was added; the mixture was flushed several times with H_2_ gas. The reaction was stirred overnight at 5 bar H_2_ atmosphere. The solvent was evaporated and the residue was purified by fc (CH_2_Cl_2_:CH_3_OH = 95:5 + 1% triethylamine, Ø = 2 cm, l = 20 cm, V = 10 mL) to afford the amine as a colorless solid (R_f_ = 0.38, CH_2_Cl_2_:CH_3_OH = 95:5 + 1% triethylamine), yield 0.018 g (60%). C_21_H_30_N_6_O_4_S (462.57 g/mol). Melting point: 111.4 °C. Purity (HPLC: method D): 90% (t_R_ = 15.74 min). Exact mass (LC-MS-ESI): *m*/*z* calculated for C_21_H_31_N_6_O_4_S [M + H]^+^ 463.2122, found 463.2125. ^1^H-NMR (600 MHz, DMSO-*d*_6_) δ (ppm) = 8.45 (s, 1H, 8-C*H_purine_*), 5.26 (d, *J* = 7.1 Hz, 1H, 2C*H_bicyclohexane_*), 4.93 (s, 1H, 4-C*H_bicyclohexane_*), 4.73 (dd, *J* = 7.3, 1.5 Hz, 1H, 3C*H_bicyclohexane_*), 3.17 (s, 0.3H, C*H*_3_OH, solvent: methanol), 2.95 (d, *J* = 13.3 Hz, 1H, NC*H*H), 2.82 (d, *J* = 13.3 Hz, 1H, NCH*H*), 2.59 (s, 3H, SC*H*_3_), 1.68 (ddd, *J* = 8.3, 5.2, 1.5 Hz, 1H, 5C*H_bicyclohexane_*), 1.48 (s, 9H, C(C*H*_3_)_3_), 1.45 (s, 3H, C(C*H*_3_)_2_), 1.18 (s, 3H, C(C*H*_3_)_2_), 0.94–0.90 (m, 2H, 6C*H_2 bicyclohexane_*). ^13^C-NMR (151 MHz, DMSO-*d*_6_) δ (ppm) = 163.7 (1C, *C*-2*_purine_*), 152.0 (1C, *C*-4*_purine_*), 150.9 (1C, *C* = O), 149.6 (1C, *C*-6*_purine_*), 141.5 (1C, *C*-8*_purine_*), 120.7 (1C, *C*-5*_purine_*), 111.3 (1C, *C*(CH_3_)_2_), 88.3 (1C, *C*-3*_bicyclohexane_*), 82.1 (1C, *C*-2*_bicyclohexane_*), 80.2 (1C, *C*(CH_3_)_3_), 59.2 (1C, *C*-4*_bicyclohexane_*), 43.6 (1C, N*C*H_2_), 38.4 (1C, *C*-1*_bicyclohexane_*), 29.7 (1C, *C*5*_bicyclohexane_*), 27.9 (3C, C(*C*H_3_)_3_), 25.9 (1C, C(*C*H_3_)_2_), 24.2 (1C, C(*C*H_3_)_2_), 14.0 (1C, S*C*H_3_), 13.3 (1C, *C*-6*_bicyclohexane_*). FT-IR (neat) *ṽ* (cm^−1^) = 2978, 2928 (C-H_aliphat_.), 1751, 1721 (C = O), 1605, 1574 (C = C_aromat_.), 1142, 1053 (C-O).

The amine (0.105 g, 0.23 mmol) was dissolved in CH_2_Cl_2_ (2 mL). Dimethyl squarate (0.104 g, 0.73 mmol, 3.2 eq.) and triethylamine (0.20 mL, 1.44 mmol, 6.4 eq.) were added and the mixture was stirred at rt overnight. The solvent was evaporated and the residue was purified by fc (CH_3_CN:H_2_O = 5:95 ⭢ 100:0, 12 mL/min, Biotage^®®^ SNAP Ultra C18, 12 g, V = 20 mL) to afford the squaramide as a colorless solid (R_f_ = 0.33, ethyl acetate = 100%), yield 0.087 g (67%). C_26_H_32_N_6_O_7_S (572.64 g/mol). Melting point: 148.4 °C. Purity (HPLC: method B): 90% (t_R_ = 13.02 min). Exact mass (LC-MS-ESI): *m*/*z* calculated for C_26_H_33_N_6_O_7_S [M + H]^+^ 573.2126, found 573.2130. The compound shows two different rotamers a and b in the NMR spectra in a ratio of approximately 5:4. ^1^H-NMR (600 MHz, DMSO-*d*_6_) δ (ppm) = 10.09 (s, 1H, N*H_purine, rotam_. _a/b_*), 8.94 (s, 0.5H, N*H_squaramide, rotam_. _a_*), 8.74 (s, 0.4H, N*H_squaramide, rotam_. _b_*), 8.26 (s, 1H, 8-C*H_purine, rotam_. _a/b_*), 5.25 (t, *J* = 6.6 Hz, 1H, 2-C*H_bicyclohexane, rotam_. _a/b_*), 4.93 (s, 1H, 4-C*H_bicyclohexane, rotam_. _a/b_*), 4.88 (d, *J* = 7.1 Hz, 0.4H, 3-C*H_bicyclohexane, rotam_. _b_*), 4.83 (d, *J* = 7.2 Hz, 0.5H, 3-C*H_bicyclohexane, rotam_. _a_*), 4.29 (s, 1.2H, OC*H_3, rotam_. _b_*), 4.24 (s, 1.5H, OC*H_3, rotam_. _a_*), 3.77 (q, *J* = 14.2 Hz, 0.9H, NC*H_2, rotam_. _b_*), 3.69 (d, *J* = 13.2 Hz, 1H, NC*H*H*_rotam_. _a_*), 3.50 (d, *J* = 13.2 Hz, 1H, NCH*H_rotam_. _a_*), 3.17 (s, 0.2H, C*H*_3_OH, solvent: methanol), 2.58 (d, *J* = 4.0 Hz, 3H, SC*H_3, rotam_. _a/b_*), 1.77 (dt, *J* = 13.0, 6.7 Hz, 1H, 5-C*H_bicyclohexane, rotam_. _a/b_*), 1.48 (s, 9H, C(C*H*_3_)*_3, rotam_. _a/b_*), 1.45 (s, 3H, C(C*H*_3_)_2*,*_*_rotam_. _a/b_*), 1.18 (s, 3H, C(C*H*_3_)_2*,*_*_rotam_. _a/b_*), 0.98 (d, *J* = 7.0 Hz, 1H, 6-C*H_2 bicyclohexane, rotam_. _a/b_*). ^13^C-NMR (151 MHz, DMSO-*d*_6_) δ (ppm) = 189.3 (1C, *C*-1*_squaramide, rotam_. _a/b_*), 182.5 (0.3C, *C*-2*_squaramide, rotam_. _a_*), 182.1 (0.3C, *C*-2*_squaramide, rotam_. _b_*), 177.7 (0.3C, *C*-4*_squaramide, rotam_. _b_*), 176.7 (0.4C, *C*-4*_squaramide, rotam_. _a_*), 172.4 (0.4C, *C*-3*_squaramide, rotam_. _a_*), 171.9 (0.3C, *C*-3*_squaramide, rotam_. _a_*), 163.8 (1C, *C*-2*_purine, rotam_. _a/b_*), 152.0 (1C, *C*-4*_purine, rotam_. _a/b_*), 150.8 (1C, *C*-N*_carbonyl, rotam_. _a/b_*), 149.6 (1C, *C*-6*_purine, rotam_. _a/b_*), 141.4 (1C, *C*-8*_purine, rotam_. _a/b_*), 120.8 (0.2C, *C*-5*_purine, rotam_. _b_*), 120.7 (0.3C, *C*-5*_purine, rotam_. _a_*), 111.4 (1C, *C*(CH_3_)_2*,*_*_rotam_. _a/b_*), 88.2 (0.6C, *C*-3*_bicyclohexane, rotam_. _a_*), 88.0 (0.5C, *C*-3*_bicyclohexane, rotam_. _b_*), 82.4 (0.8C, *C*-2*_bicyclohexane, rotam_. _a_*), 82.1 (0.6C, *C*-2*_bicyclohexane, rotam_. _b_*), 80.2 (1C, *C*(CH_3_)_3*,*_*_rotam_. _a/b_*), 60.1 (0.6C, O*C*H_3*,*_*_rotam_. _a_*), 60.0 (0.5C, O*C*H_3*,*_*_rotam_. _b_*), 59.3 (1C, *C*-4*_bicyclohexane, rotam_. _a/b_*), 46.7 (0.7C, N*C*H_2*,*_*_rotam_. _a_*), 46.1 (0.5C, N*C*H_2*,*_*_rotam_. _b_*), 37.5 (0.4C, *C*-1*_bicyclohexane, rotam_. _b_*), 37.2 (0.5C, *C*-1*_bicyclohexane, rotam_. _a_*), 30.3 (1C, *C*-5*_bicyclohexane, rotam_. _a/b_*), 27.9 (3C, C(*C*H_3_)*_3, rotam_. _a/b_*), 25.9 (1C, C(*C*H_3_)_2*,*_*_rotam_. _a/b_*), 24.1 (1C, C(*C*H_3_)_2*,*_*_rotam_. _a/b_*), 14.0 (1C, S*C*H_3*,*_*_rotam_. _a/b_*), 13.2 (0.5C, *C*-6*_bicyclohexane, rotam_. _b_*), 13.0 (0.6C, *C*-6*_bicyclohexane, rotam_. _a_*). FT-IR (neat) *ṽ* (cm^−1^) = 3202 (N-H), 2986, 2936 (C-H_aliphat_.), 1802, 1755, 1709 (C = O), 1601 (C = C_aromat_.), 1138, 1049 (C-O).

The squaramide (0.085 g, 0.15 mmol) was dissolved in CH_3_OH (3.2 mL) and trifluoroacetic acid (0.40 mL) and H_2_O (0.40 mL) were added. The mixture was stirred at rt for 5 d and was then heated to 70° C for 1 d. The solvent was evaporated and the residue was purified by semi-preparative HPLC (method G) to afford the product **47** as a colorless solid, yield 0.021 g (27%). C_23_H_33_N_7_O_5_S (519.62 g/mol). Purity (HPLC: method D): 99% (t_R_ = 6.26 min). Exact mass (LC-MS-ESI): *m*/*z* calculated for C_17_H_17_N_6_O_5_S [M-C_6_H_16_N]^−^ 417.0987, found 417.0984. The compound **47** shows three different rotamers a, b and c in the NMR spectra in a ratio of approximately 10:2:1. ^1^H-NMR (600 MHz, DMSO-*d*_6_) δ (ppm) = 9.36 (s, 0.9H, N*H_triethylammonium_*), 7.94 (s, 1H, 8-C*H_purine, rotam_. _a_*), 7.90 (s, 0.2H, 8-C*H_purine, rotam_. _b_*), 7.78 (s, 0.1H, 8-C*H_purine, rotam_. _c_*), 7.40 (t, *J* = 6.6 Hz, 1H, N*H_squaramide, rotam_. _a_*), 7.36 (s, 0.4H, N*H_squaramide, rotam_. _b, c_*), 5.34 (d, *J* = 7.7 Hz, 1H, 2-C*H_bicyclohexane, rotam_. _b_*), 5.16 (s, 0.4H, 3-O*H_bicyclohexane, rotam_. _b, c_*), 5.02 (s, 1H, 3-O*H_bicyclohexane, rotam_. _a_*), 4.85 (s, 0.2H, 4-C*H_bicyclohexane, rotam_. _b_*), 4.72 (d, *J* = 7.1 Hz, 0.1H, 2-C*H_bicyclohexane, rotam_. _c_*), 4.67 (s, 0.1H, 4-C*H_bicyclohexane, rotam_. _c_*), 4.63 (d, *J* = 8.1 Hz, 0.2H, 3-C*H_bicyclohexane, rotam_. _b_*), 4.61 (d, *J* = 1.6 Hz, 1H, 4-C*H_bicyclohexane, rotam_. _a_*), 4.44 (d, *J* = 6.3 Hz, 1H, 2-C*H_bicyclohexane, rotam_. _a_*), 3.99 (dd, *J* = 14.2, 6.8 Hz, 1H, NC*H*H*_rotam_. _a_*), 3.89–3.80 (m, 0.3H, 3-C*H_bicyclohexane, rotam_. _c_*, NC*H*H*_rotam_. _c_*), 3.80–3.72 (m, 1.3H, NC*H*H*_rotam_. _b_*, 3-C*H_bicyclohexane, rotam_. _a_*), 3.69 (dd, *J* = 14.1, 6.1 Hz, 1H, NCH*H_rotam_. _b_*), 3.513.47 (m, 0.2H, -NCH*H_rotam_. _c_*), 3.30 (dd, *J* = 14.3, 6.2 Hz, 1H, NCH*H_rotam_. _a_*), 3.09 (q, *J* = 7.3 Hz, 6.6H, C*H_2, triethylammonium_*), 2.49 (s, 0.6H, SC*H_3, rotam_. _b_*), 2.48 (s, 0.2H, SC*H_3, rotam_. _c_*), 2.47 (s, 3H, SC*H_3, rotam_. _a_*), 1.84 (ddd, *J* = 9.0, 4.2, 1.5 Hz, 0.2H, 5-C*H_bicyclohexane, rotam_. _b_*), 1.58 (dd, *J* = 8.3, 3.8 Hz, 1H, 5-C*H_bicyclohexane, rotam_. _a_*), 1.52 (s, 0.1H, 6-C*H*H*_bicyclohexane, rotam_. _c_*), 1.48 (dd, *J* = 8.4, 4.3 Hz, 0.1H, 5-C*H_bicyclohexane, rotam_. _c_*), 1.34 (t, *J* = 4.3 Hz, 1H, 6-C*H*H*_bicyclohexane, rotam_. _a_*), 1.16 (t, *J* = 7.3 Hz, 9.9H, 3-C*H_3, triethylammonium_*), 1.09–1.06 (m, 0.3H, 6-C*H*H*_bicyclohexane, rotam_. _c_*), 0.66 (ddd, *J* = 8.5, 4.6, 1.5 Hz, 1H, 6-CH*H_bicyclohexane, rotam_. _a_*), 0.65–0.62 (m, 0.2H, 6-CH*H_bicyclohexane, rotam_. _b_*), 0.57 (dd, *J* = 9.0, 4.5 Hz, 0.1H, 6-CH*H_bicyclohexane, rotam_. _c_*). ^13^C-NMR (151 MHz, DMSO-*d*_6_) δ (ppm) = 198.1 (0.5C, *C*-4*_squaramide, rotam_. _a, b_*), 188.7 (2C, *C*-1*_squaramide, rotam_. _a_*, *C*-3*_squaramide, rotam_. _a_*), 188.4 (0.4C, *C*-1*_squaramide, rotam_. _b_*, *C*-3*_squaramide, rotam_. _b_*), 181.5 (1C, *C*-2*_squaramide, rotam_. _a_*), 181.4 (0.3C, *C*-2*_squaramide, rotam_. _b_*), 164.1 (0.1C, *C*-2*_purine, rotam_. _b_*), 163.7 (1C, *C*-2*_purine, rotam_. _a_*), 155.4 (1C, *C*-6*_purine, rotam_. _a_*), 155.4 (0.2C, *C*-6*_purine, rotam_. _b_*), 149.8 (1C, *C*-4*_purine, rotam_. _a_*), 149.5 (0.2C, *C*-4*_purine, rotam_. _b_*), 137.5 (0.1C, *C*-8*_purine, rotam_. _b_*), 137.3 (1C, *C*-8*_purine, rotam_. _a_*), 116.5 (0.1C, *C*-5*_purine, rotam_. _b_*), 116.5 (1C, *C*-5*_purine, rotam_. _a_*), 87.7 (0.2C, *C*-3*_bicyclohexane, rotam_. _b_*), 80.8 (0.2C, *C*-2*_bicyclohexane, rotam_. _b_*), 76.7 (1C, *C*-3*_bicyclohexane, rotam_. _a_*), 71.0 (1C, *C*-2*_bicyclohexane, rotam_. _a_*), 60.6 (1C, *C*-4*_bicyclohexane, rotam_. _a_*), 60.6 (0.3C, *C*-4*_bicyclohexane, rotam_. _b_*), 45.7 (5.0C, *C*H_2,_ *_triethylammonium_*), 44.7 (1C, N*C*H_2,_ *_rotam_. _a_*), 44.6 (0.2C, N*C*H_2,_ *_rotam_. _b_*), 35.8 (1C, *C*-1*_bicyclohexane, rotam_. _a_*), 27.5 (0.2C, *C*-5*_bicyclohexane, rotam_. _b_*), 22.5 (C, *C*-5*_bicyclohexane, rotam_. _a_*), 13.7 (0.2C, S*C*H_3,_ *_rotam_. _b_*), 13.6 (1C, S*C*H_3,_ *_rotam_. _a_*), 11.8 (0.2C, *C*-6*_bicyclohexane, rotam_. _b_*), 11.7 (1C, *C*-6*_bicyclohexane, rotam_. _a_*), 8.6 (4.6C, *C*H_3,_ *_triethylammonium_*); the signal for *C*-1*_bicyclohexane, rotam_. _b_* is located under the DMSO-*d*_6_ signal and therefore only visible in 2D spectra. The resolution was too low to identify the ^13^C signals for rotamer c, therefore only rotamer a and b are described here. FT-IR (neat) *ṽ* (cm^−1^) = 3321 (O-H), 3190 (N-H), 2924 (C-H_aliphat_.), 1786 (C = O), 1528 (C = C_aromat_.), 1065 (C-O).

*Triethylammonium 2-(N-{[(1R,2R,3S,4R,5S)-4-(6-amino-2-methylthio-9H-purin-9-yl)-2,3-dihydroxybicyclo[3.1.0]hex-1-yl]methyl}sulfamoyl)acetate* (**48**). The azide **38** (0.032 g, 0.07 mmol) was dissolved in CH_3_OH (2 mL) and Pd/C (10 wt%, 0.003 g, 0.003 mmol, 0.004 eq.) was added; the mixture was flushed several times with H_2_ gas. The reaction was stirred overnight at 5 bar H_2_ atmosphere. The solvent was evaporated and the residue was purified by fc (CH_2_Cl_2_:CH_3_OH = 95:5 + 1% triethylamine, Ø = 2 cm, l = 20 cm, V = 10 mL) to afford the amine as a colorless solid (R_f_ = 0.38, CH_2_Cl_2_:CH_3_OH = 95:5 + 1% triethylamine), yield 0.018 g (60%).

The amine (0.16 g, 0.35 mmol) was dissolved in CH_2_Cl_2_ (8 mL) and methyl 2-(chlorosulfonyl)acetate (0.068 g, 0.39 mmol, 1.1 eq.), triethylamine (0.10 mL, 0.72 mmol, 2 eq.), and a catalytic amount of DMAP (~5 mol%) were added. The mixture was stirred at rt overnight. The solvent was evaporated and the residue was purified by fc (CH_3_CN:H_2_O = 5:95 ⭢ 65:35, 12 mL/min, Biotage^®®^ SNAP Ultra C18, 12 g, V = 20 mL) to afford the sulfonamide as a colorless solid (R_f_ = 0.49, ethyl acetate = 100%), yield 0.103 g (50%). C_24_H_34_N_6_O_8_S_2_ (598.69 g/mol). Purity (HPLC: method B): 90% (t_R_ = 14.49 min). Exact mass (LC-MS-ESI): *m*/*z* calculated for C_24_H_35_N_6_O_8_S [M + H]^+^ 599.1952, found 599.1952. ^1^H-NMR (400 MHz, DMSO-*d*_6_) δ (ppm) = 10.09 (s, 1H, N*H_purine_*), 8.33 (s, 1H, 8C*H_purine_*), 7.76 (t, *J* = 6.4 Hz, 1H, N*H_sulfonamide_*), 5.75 (s, 0.2H, C*H*_2_Cl_2_, solvent: dichloromethane), 5.20 (dd, *J* = 7.2, 1.2 Hz, 1H, 2-C*H_bicyclohexane_*), 4.93 (s, 1H, 4C*H_bicyclohexane_*), 4.75 (dd, *J* = 7.3, 1.3 Hz, 1H, 3C*H_bicyclohexane_*), 4.23 (dd, *J* = 14.2, 1.6 Hz, 2H, 2-C*H*_2_), 3.69 (s, 1H, OC*H*_3_), 3.34–3.28 (m, 2H, NC*H*_2_), 2.59 (s, 3H, SC*H*_3_), 1.71 (ddd, *J* = 9.0, 4.7, 1.5 Hz, 1H, 5C*H_bicyclohexane_*), 1.48 (s, 9H, C(C*H*_3_)_3_), 1.46 (s, 3H, C(C*H*_3_)_2_), 1.21–1.15 (m, 3H, C(C*H*_3_)_2_), 1.03–0.95 (m, 2H, 6-C*H_2 bicyclohexane_*). ^13^C-NMR (101 MHz, DMSO-*d*_6_) δ (ppm) = 164.0 (1C, *C*-1), 163.8 (1C, *C*-2*_purine_*), 152.0 (1C, *C*4*_purine_*), 150.8 (1C, *C*-N*_carbonyl_*), 149.6 (1C, *C*-6*_purine_*), 141.1 (1C, *C*-8*_purine_*), 120.7 (1C, *C*-5*_purine_*), 111.4 (1C, *C*(CH_3_)_2_), 88.2 (1C, *C*-3*_bicyclohexane_*), 81.9 (1C, *C*-2*_bicyclohexane_*), 80.2 (1C, *C*(CH_3_)_3_), 58.9 (1C, *C*-4*_bicyclohexane_*), 56.1 (1C, *C*-2), 52.5 (1C, O*C*H_3_), 45.5 (1C, N*C*H_2_), 36.8 (1C, *C*-1*_bicyclohexane_*), 29.9 (1C, *C*5*_bicyclohexane_*), 27.8 (3C, C(*C*H_3_)_3_), 25.8 (1C, C(*C*H_3_)_2_), 24.2 (1C, C(*C*H_3_)_2_), 14.0 (1C, S*C*H_3_), 13.4 (1C, *C*-6*_bicyclohexane_*). FT-IR (neat) *ṽ* (cm^−1^) = 3283 (N-H), 2982, 2932 (C-H_aliphat_.), 1744 (C = O), 1605, 1578 (C = C_aromat_.), 1327, 1207 (S = O), 1138, 1053 (C-O).

The sulfonamide (0.090 g, 0.15 mmol) was dissolved in CH_3_OH (3.2 mL) and trifluoroacetic acid (0.40 mL) and H_2_O (0.40 mL) were added. The mixture was stirred at 70 °C overnight. The solvent was evaporated, and the residue was dissolved in CH_3_CN (3.2 mL), 2 M NaOH-solution and H_2_O (0.40 mL) were added. The mixture was stirred at 70° C overnight. The solvent was evaporated, and the residue was purified by semi-preparative HPLC (method E) to afford the product **48** as a colorless solid, yield 0.056 g (69%). C_21_H_35_N_7_O_6_S_2_ (545.21 g/mol). Purity (HPLC: method D): 99% (t_R_ = 9.86 min). Exact mass (LC-MS-ESI): *m*/*z* calculated for C_15_H_19_N_6_O_6_S_2_ [M-C_6_H_16_N]^−^ 443.0813, found 443.0826. The compound **48** shows two different rotamers a and b in the NMR spectra in a ratio of approximately 2:1. ^1^H-NMR (600 MHz, DMSO-*d*_6_) δ (ppm) = 8.10 (s, 1H, 8-C*H_purine, rotam_. _a_*), 7.97 (s, 0.5H, 8C*H_purine, rotam_. _b_*), 7.29 (s, 3H, N*H_2, rotam_. _a_*, N*H_rotam_. _a_*), 7.25 (s, 1.5H, N*H_2, rotam_. _b_*, N*H_rotam_. _b_*), 5.12 (s, 1H, 3O*H_bicyclohexane, rotam_. _a_*), 4.78 (s, 0.3H, 2C*H_bicyclohexane, rotam_. _b_*), 4.70 (s, 0.5H, 4C*H_bicyclohexane, rotam_. _b_*), 4.61 (d, *J* = 1.9 Hz, 1H, 4-C*H_bicyclohexane, rotam_. _a_*), 4.51 (dd, *J* = 6.5, 1.4 Hz, 1H, 2C*H_bicyclohexane, rotam_. _a_*), 4.01 (s, 0.3H, 3C*H_bicyclohexane, rotam_. _b_*), 3.91 (s, 0.5H, 2-C*H*H*_rotam_. _b_*), 3.84 (dt, *J* = 6.7, 1.6 Hz, 1H, 3C*H_bicyclohexane, rotam a_*), 3.73 (dd, *J* = 14.6, 5.1 Hz, 2H, 2-C*H_2 rotam_. _a_*, 2CH*H_rotam_. _b_*), 3.39 (d, *J* = 13.5 Hz, 1H, NC*H*H*_rotam a_*), 3.25 (s, 0.5H, NC*H*H*_rotam b_*), 3.13 (s, 0.5H, NCH*H_rotam b_*), 3.07 (d, *J* = 13.5 Hz, 1H, NCH*H_rotam a_*), 2.93 (q, *J* = 7.5 Hz, 7.8H, C*H_2, triethylammonium_*), 2.48 (s, 3H, SC*H_3, rotam_. _a_*), 1.54 (t, *J* = 4.6 Hz, 0.5H, 6C*H*H*_bicyclohexane, rotam_. _b_*), 1.48 (dd, *J* = 8.5, 3.8 Hz, 1H, 5C*H_bicyclohexane, rotam_. _a_*), 1.33 (dd, *J* = 8.7, 4.8 Hz, 0.5H, 5C*H_bicyclohexane, rotam_. _b_*), 1.30 (t, *J* = 4.4 Hz, 1H, 6C*H*H*_bicyclohexane, rotam_. _a_*), 1.11 (t, *J* = 7.3 Hz, 12.3H, C*H_3, triethylammonium_*), 0.72 (ddd, *J* = 8.6, 4.7, 1.5 Hz, 1H, 6CH*H_bicyclohexane, rotam_. _a_*), 0.62 (dd, *J* = 9.6, 4.8 Hz, 0.5H, 6CH*H_bicyclohexane, rotam_. _b_*); the signal for SC*H_3, rotam_. _b_* is located under the DMSO-*d*_6_ signal and therefore only visible in 2D spectra. ^13^C-NMR (151 MHz, DMSO-*d*_6_) δ (ppm) = 165.0 (0.7C, *C*-1*_rotam_. _a, b_*), 163.7 (1C, *C*2*_purine, rotam_. _a_*), 163.6 (0.4C, *C*-2*_purine, rotam_. _b_*), 155.4 (1C, *C*-6*_purine, rotam_. _a_*), 155.4 (0.4C, *C*6*_purine, rotam_. _b_*), 149.8 (1C, *C*4*_purine, rotam_. _a_*), 149.6 (0.4C, *C*4*_purine, rotam_. _b_*), 138.1 (0.2C, *C*8*_purine, rotam_. _b_*), 137.9 (1C, *C*8*_purine, rotam_. _a_*), 116.5 (0.8C, *C*-5*_purine, rotam_. _a, b_*), 76.9 (1C, *C*3*_bicyclohexane, rotam_. _a_*), 71.6 (1C, *C*2*_bicyclohexane, rotam_. _a_*), 61.0 (1C, *C*-4*_bicyclohexane, rotam_. _a_*), 57.4 (1C, *C*-2*_rotam_. _a_*), 45.7 (1C, N*C*H_2,_ *_rotam_. _a_*), 45.3 (6.3C, *C*H_2,_ *_triethylammonium_*), 34.5 (1C, *C*-1*_bicyclohexane, rotam_. _a_*), 27.6 (0.4C, *C*5*_bicyclohexane, rotam_. _b_*), 23.2 (1C, *C*5*_bicyclohexane, rotam_. _a_*), 13.7 (1.3C, S*C*H_3,_ *_rotam_. _a, b_*), 12.6 (0.4C, *C*-6*_bicyclohexane, rotam_. _b_*), 12.5 (1C, *C*-6*_bicyclohexane, rotam_. _a_*), 9.0 (5.6C, *C*H_3,_ *_triethylammonium_*); the resolution was too low to identify the ^13^C signals for rotamer b, therefore only those signals of rotamer b that are visible in the ^13^C-NMR spectrum are described here. FT-IR (neat) *ṽ* (cm^−1^) = 3306 (O-H), 3132 (N-H), 2982, 2928 (C-H_aliphat_.), 1612 (C = O), 1582 (C = C_aromat_.), 1300 (S = O), 1150, 1119 (C-O), 1065 (S = O).

## Figures and Tables

**Table 1:** Inhibitory activities of the novel compounds in human adenosine receptor binding assays (*n* = 3), ^a^ % displacement by the test compound at a concentration of 10 µM, or at 1 µM. Bn = benzyl, PMB = *p*-methoxybenzyl.

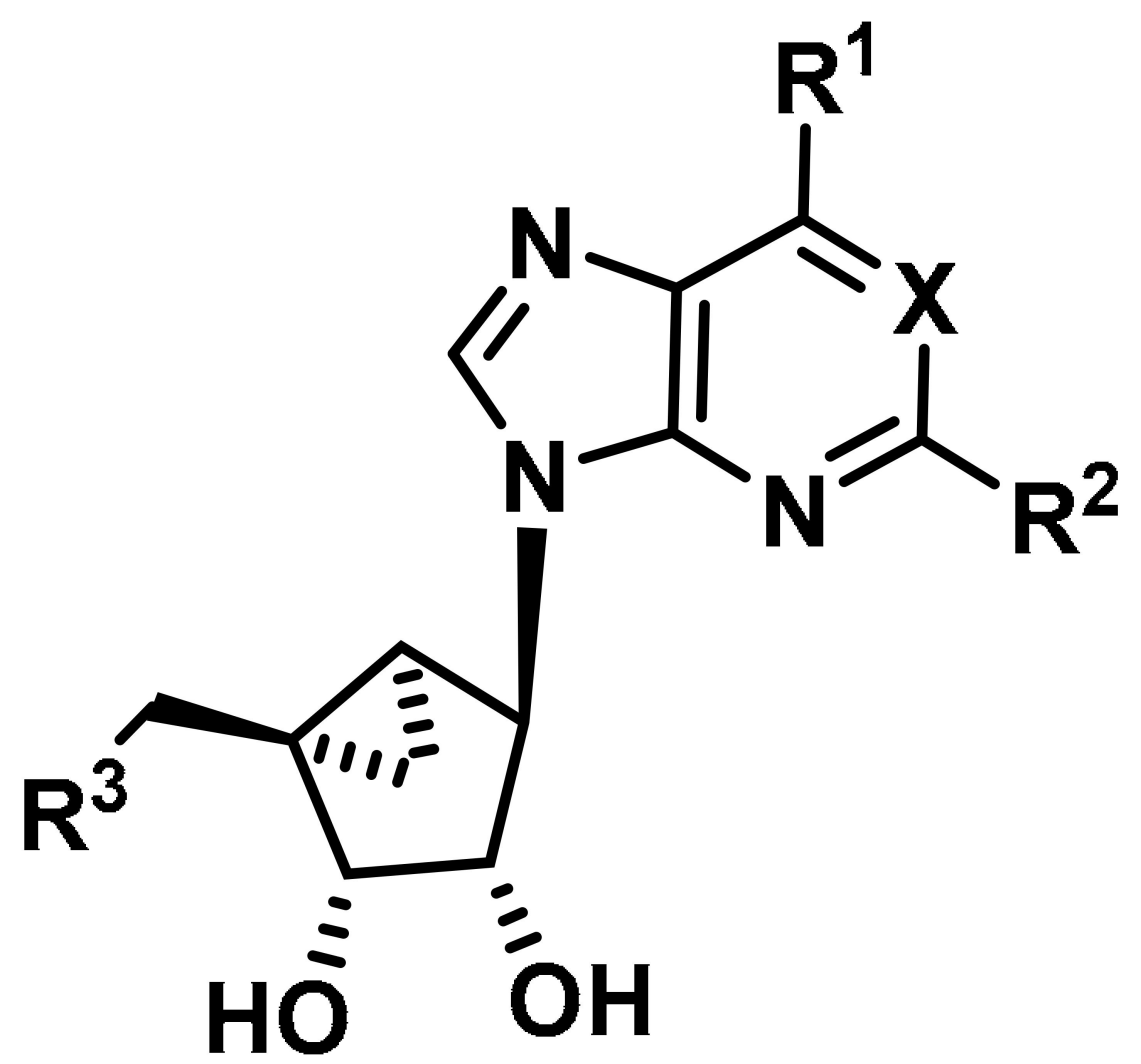								
	K*i* ± SEM [µM] or displacement [%] at 10 µM, unless noted							
**Cmpd.**	**R^1^**	**R^2^**	**R^3^**	**X**	**A_1_**	**A_2A_**	**A_2B_**	**A_3_**
**11**	Cl	NH_2_	OH	CH	5%	4%	>10	4%
**12**	Cl	Cl	OH	CH	>10	>10	>10	>10
**13**	NHBn	Cl	OH	CH	25%	13%	>10	0.46 ± 0.02
**14**	NHBn	SCH_3_	OH	CH	20%	4%	>10	1.51 ± 0.03
**15**	NHPMB	Cl	OH	CH	12%	2%	>10	0.50 ± 0.01
**16**	NH_2_	Cl	OH	CH	32%	42%	>10	1.60 ± 0.11
**17**	NH_2_	SCH_3_	OH	CH	23%	3%	>10	45%
**26**	NBn_2_	H	OH	N	25%	9%	12%	8%
**27**	NBn_2_	Cl	OH	N	15%	>10	>10	4%
**30**	NBn_2_	SCH_3_	OH	N	30%	13%	>10	0.38 ± 0.01
**31**	NBn_2_	SCH_3_	Cl	N	21%	>10	>10	26%
**36**	NH_2_	H	OH	N	6.10 ± 0.46	1.81 ± 0.11	>10	0.96 ± 0.05
**39**	NH_2_	SCH_3_	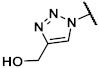	N	5%	11%	>10	37%
**43**	NH_2_	SCH_3_	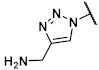	N	5%	3%	>10	13%
**40**	NH_2_	SCH_3_	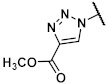	N	7%	3%	>10	8%
**44**	NH_2_	SCH_3_	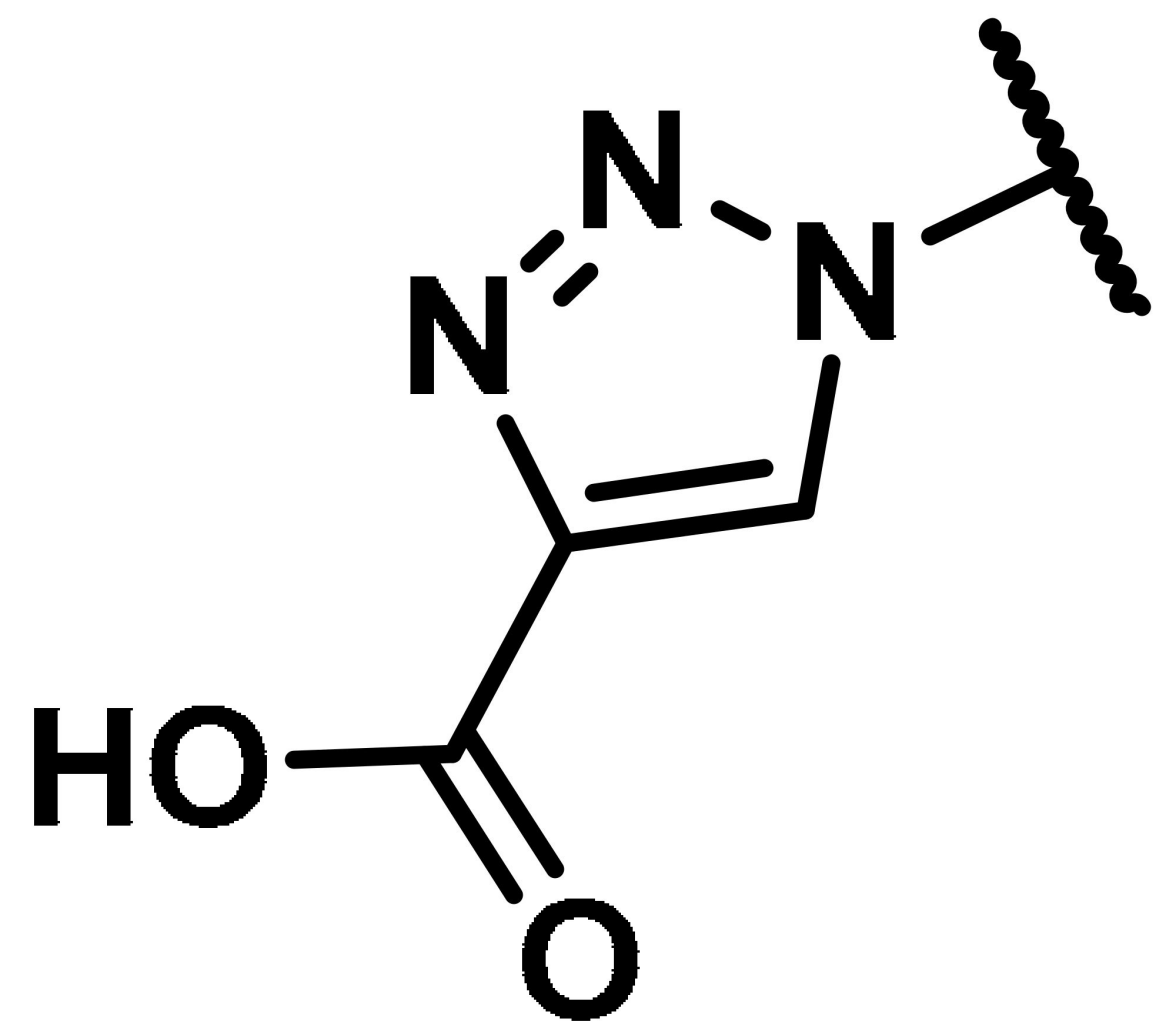	N	>1 ^a^	>1 ^a^	2% ^a^	>1 ^a^
**41**	NH_2_	SCH_3_	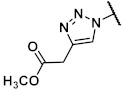	N	2% ^a^	>1 ^a^	>1 ^a^	3% ^a^
**45**	NH_2_	SCH_3_	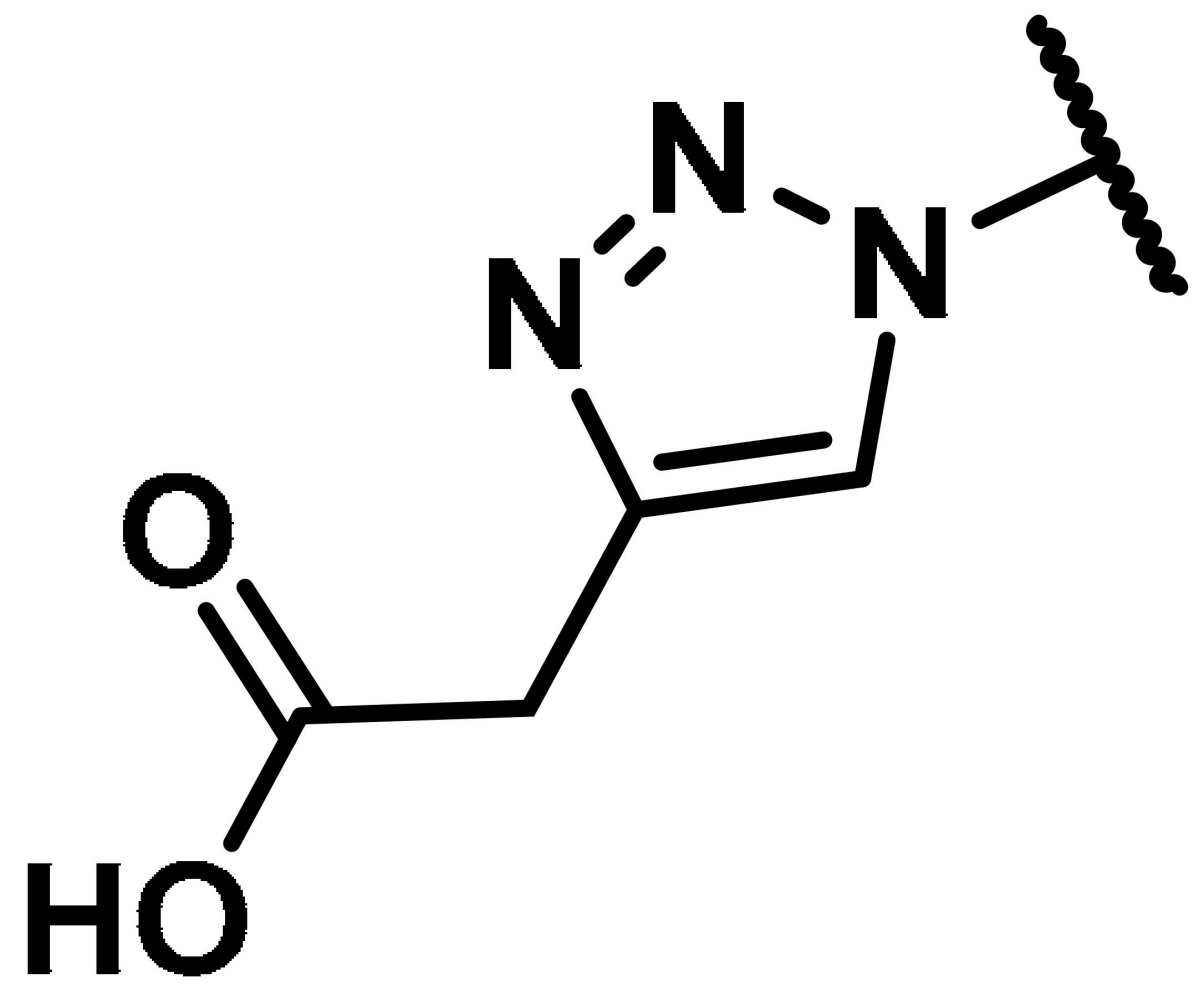	N	>1 ^a^	>1 ^a^	5% ^a^	>1 ^a^
**42**	NH_2_	SCH_3_	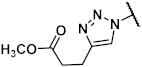	N	19%	29%	>10	6.35 ± 0.23
**46**	NH_2_	SCH_3_	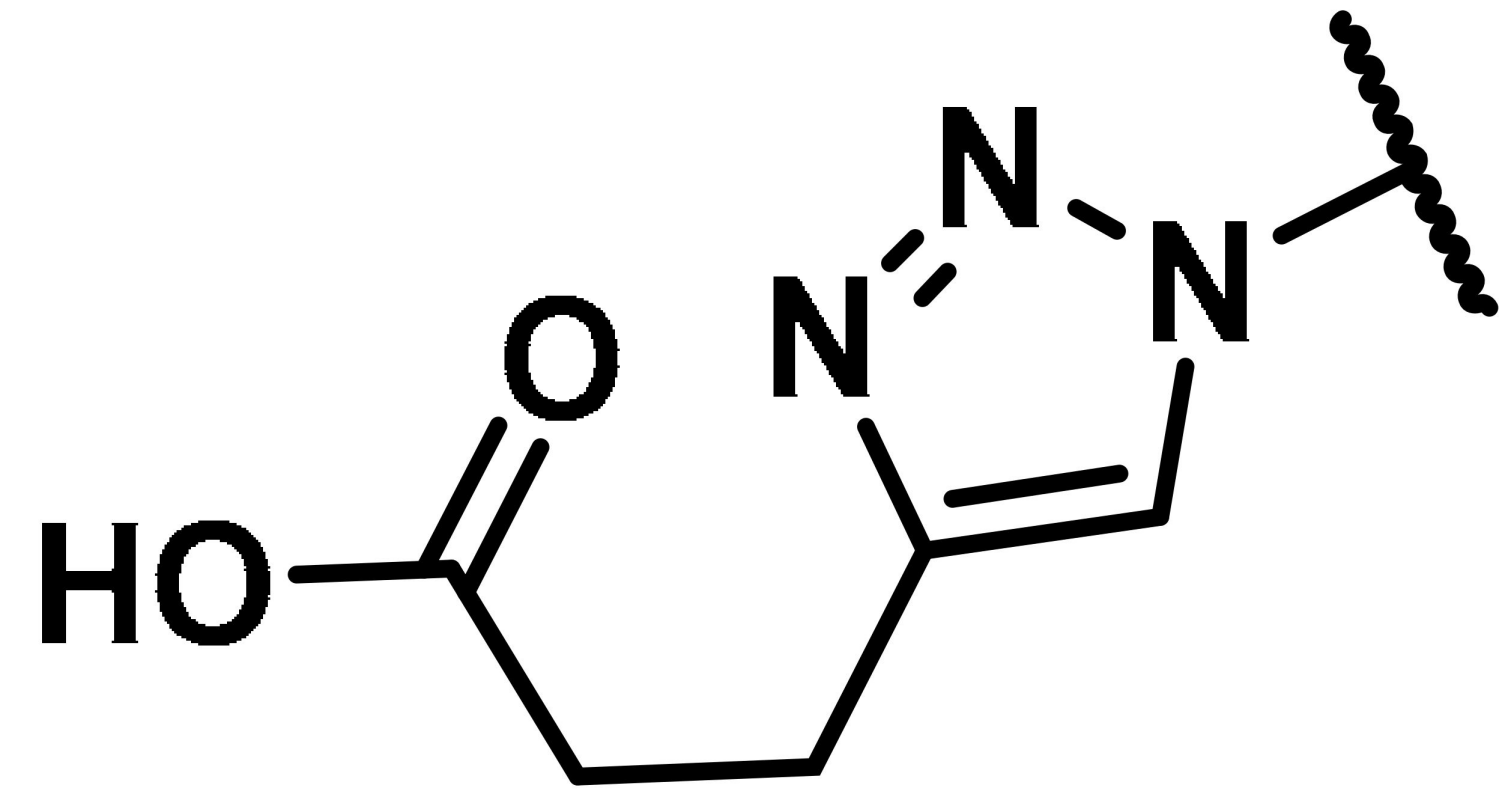	N	>1 ^a^	>1 ^a^	1% ^a^	>1 ^a^
**47**	NH_2_	SCH_3_	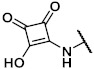	N	1% ^a^	>1 ^a^	>1 ^a^	6% ^a^
**48**	NH_2_	SCH_3_	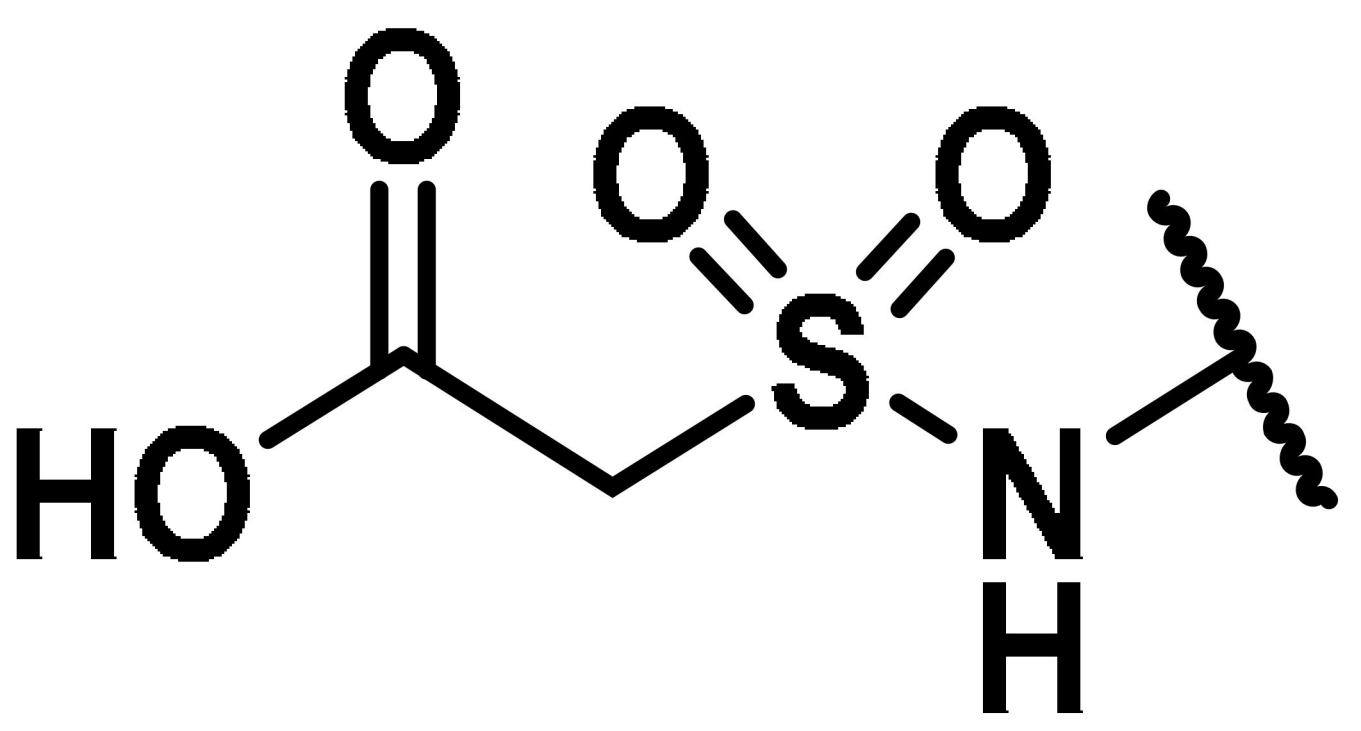	N	3% ^a^	>1 ^a^	>1 ^a^	5% ^a^

## Data Availability

All data generated or analyzed during this study are included in this article.

## Supplementary Materials

The following supporting information can be downloaded at: https://www.mdpi.com/article/10.3390/molecules27072283/s1. Synthetic procedures for the preparation of compound **4** and mass spectra and NMR spectra of compound **4**.

## Sample Availability

Samples of the compounds **36**, **39**–**48** are available from the authors.

## Abbreviations

AR, adenosine receptor; ATP, adenosine 5′-triphosphate; Bn, benzyl; Boc_2_O, di-tert-butyl dicarbonate; CCDC, Cambridge Crystallographic Data Centre; CHO, Chinese hamster ovary; cV, column volume; DAMP, damage-associated-molecular pattern; dec., decomposition; DDQ, 2,3-dichloro-5,6-dicyano-1,4-benzoquinone; DIAD, diisopropyl azodicarboxylate; DIPEA, diisopropyl ethyl amine; DMEM, Dulbecco’s modified Eagle’s medium; DMF, *N*,*N*-dimethylformamide; DMSO, dimethylsulfoxide; EGTA ethylene glycol-bis(β-aminoethyl ether)-*N*,*N*,*N*′,*N*′-tetraacetic acid; ESI, electrospray ionization; HBSS, Hanks′ balanced salt solution; HEK, human embryonic kidney cells; HEPES, 4-(2-hydroxyethyl)-1-piperazineethanesulfonic acid; HPLC, high performance liquid chromatography; LC-MS, liquid chromatography-mass spectrometry; NMP, N-methyl morpholine; PBS, phosphate-buffered saline; PMB, para-methoxybenzyl; RT, room temperature; SAR, structure-activity relationship; SEM, standard error of the mean; TBAN, tetrabutylammonium nitrate; TBDPS, tert-butyldiphenylsilyl; TFAA, trifluoroacetic acid anhydride; TFA, trifluoroacetic acid; THF, tetrahydrofuran; TLC, Thin layer chromatography.
