# Supplementary material for: Development of Bicyclo[3.1.0]hexane-Based A3 Receptor Ligands: Closing the Gaps in the Structure–Affinity Relationships
Source: Molecules. 2022 Mar 31;27(7):2283. doi: 10.3390/molecules27072283 (PMC9000336; doi:10.3390/molecules27072283)
Supplement: Supplementary file 1 [file molecules-27-02283-s001.zip › molecules-1652440-supplementary.pdf]

Supplementary Materials

Development of Bicyclo[3.1.0]hexane-Based A_3 Receptor Ligands: Closing the Gaps in the Structure–Affinity Relationships

Jan Phillip Lemmerhirt [1], Andreas Isaak [1], Rongfang Liu [2], Max Kock [1], Constantin G. Daniliuc [3], Kenneth A. Jacobson [4], Laura H. Heitman [2] and Anna Junker [1,*]

[1] European Institute for Molecular Imaging (EIMI), University of Münster, Waldeyerstr. 15, 48149 Münster, Germany; janlemmerhirt@gmail.com (J.P.L.); a_isaa01@uni-muenster.de (A.I.); max.kock@web.de (M.K.)

[2] Leiden Academic Centre for Drug Research (LACDR), Division of Medicinal Chemistry, Leiden University, Einsteinweg 55, 2333 CC Leiden, The Netherlands; r.liu@lacdr.leidenuniv.nl (R.L.); l.h.heitman@lacdr.leidenuniv.nl (L.H.H.)

[3] Organisch-Chemisches Institut; University of Münster, Correnssstraße 40, 48149 Münster, Germany; constantin.daniliuc@uni-muenster.de

[4] Molecular Recognition Section, Laboratory of Bioorganic Chemistry, National Institute of Diabetes and Digestive and Kidney Diseases, National Institutes of Health, Bethesda, MD 20892, USA; kennethj@niddk.nih.gov

* Correspondence: anna.junker@wwu.de; Tel.: +49-251-8333363

Contents — page

Synthetic procedures for the preparation of compound **4** — S2–S6

Mass Spectra and NMR Spectra of compound **4** — S7–S10

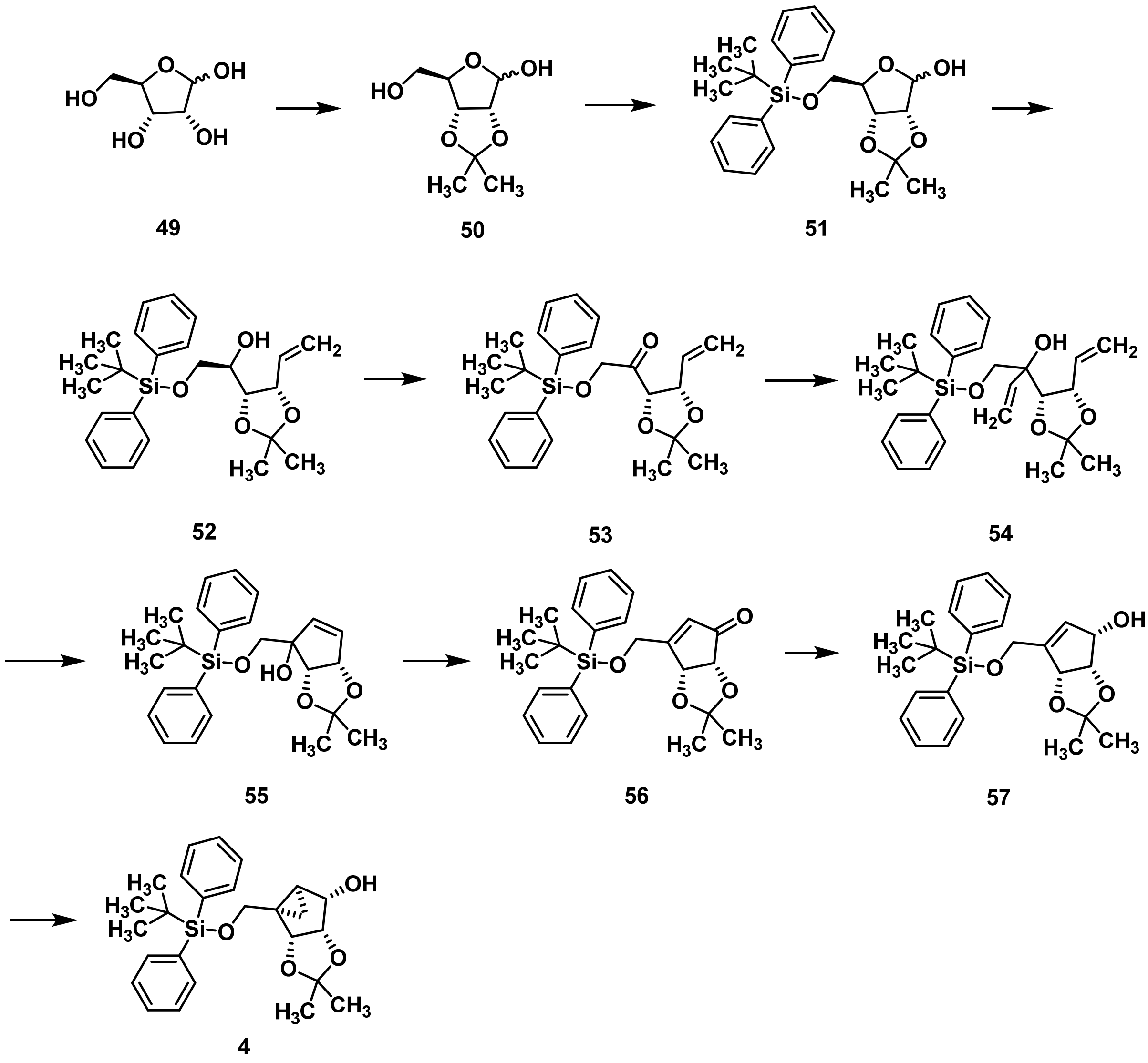

Scheme S1: Synthesis of the methanocarba alcohol **4**.

2,3-*O*-Isopropylidene-*α,β*-D-ribofuranose (50)

The procedure was modified according to reference [16].*α*,β-D-Ribose (**49**, 100.0 g, 666 mmol) was suspended in acetone (1.25 L) and sulfuric acid (3 mL, 5.52 g, 56.3 mmol, 0.08 eq.) was added dropwise. The mixture was stirred overnight at rt. The resulting solution was neutralized with solid $NaHCO_3$, filtered and concentrated in vacuo. The residue was split into four parts, each part was purified by fc (cyclohexane : ethyl acetate = 1:1, Ø = 8 cm, l = 28 cm, V = 100 mL) to afford the product **50** as a colorless oil (R_f = 0.24, cyclohexane : ethyl acetate = 1:2), yield 78.7 g (62 % as a mixture of *α* to β-isomers 1:5). $C_8H_{14}O_5$ (190.20 g/mol).

Exact mass (APCI): m/z calculated for $C_8H_{13}O_4$ $[M\text{-}OH]^+$ 173.0808, found 173.0809.

^{1}H-NMR (400 MHz, $CDCl_3$) δ (ppm) = 5.43 (s, 1H, β-1-C*H*), 5.41 (s, 0.2H, *α*-1-C*H*), 5.30 (s, 0.1H, C*H*$_2$Cl$_2$, solvent: dichloromethane), 4.86 (d, *J* = 5.9 Hz, 1H, β-3-C*H*), 4.74 (dd, *J* = 6.7, 2.4 Hz, 0.2H, *α*-3-C*H*), 4.65 (dd, *J* = 6.7, 4.2 Hz, 0.2H, *α*-2-C*H*), 4.60 (d, *J* = 5.9 Hz, 1H, β-2-C*H*), 4.42 (dd, *J* = 5.2, 2.3 Hz, 1H, β-4-C*H*), 4.19 (q, *J* = 3.2 Hz, 0.2H, *α*-4-C*H*), 3.82–3.75 (m, 1.2H, *α*-5-C*H*H, β-5-C*H*H), 3.72 (dd, *J* = 11.9, 3.0 Hz, 1H, β-5-CH*H*), 3.67 (dd, *J* = 11.7, 3.9 Hz, 0.2H, *α*-5-CH*H*), 1.58 (s, 0.6H, *α*-C(C*H*$_3$)$_2$), 1.49 (s, 3H, β-C(C*H*$_3$)$_2$), 1.4 (s, 0.6H, *α*-C(C*H*$_3$)$_2$), 1.33 (s, 3H, β-C(C*H*$_3$)$_2$).

^{13}C-NMR (101 MHz, $CDCl_3$) δ (ppm) = 114.5 (0.2C, *α*-*C*(CH_3)$_2$), 112.3 (1C, β-*C*(CH_3)$_2$), 103.3 (1C, β-*C*-1), 97.1 (0.2C, *α*-*C*-1), 88.1 (1C, β-*C*-4), 87.1 (1C, β-*C*-2), 81.9 (1C, β-*C*-3), 81.5 (0.2C, *α*-*C*-4), 81.2 (0.2C, *α*-*C*-3), 79.8 (0.2C, *α*-*C*-2), 63.9 (1C, β-*C*-5), 63.5 (0.2C, *α*-*C*-5), 26.5 (1C, β-C(*C*H$_3$)$_2$), 26.4 (0.2C, *α*-C(*C*H$_3$)$_2$), 24.9 (1.2C, *α*-C(*C*H$_3$)$_2$, β-C(*C*H$_3$)$_2$).
FT-IR (neat) $\tilde{\nu}$ (cm^{-1}) = 3372, 3352 (O-H), 2986, 2940 (C-H$_{aliphat.}$), 1034, 1061 (C-O).

5-*O*-(*tert*-Butyldiphenylsilyl)-2,3-*O*-isopropylidene-*α*,*β*-D-ribofuranose (51)
The procedure was modified according to reference [16].Compound **50** (78.5 g, 413 mmol) was dissolved in CH_2Cl_2 (1.8 L), the reaction was cooled in an ice bath and triethylamine (120 mL, 87.1 g, 861 mmol, 2.1 eq.) was added. *tert*-Butyl(chloro)-diphenylsilane (TBDPSCl, 101.5 mL, 107.3 g, 391 mmol, 0.95 eq.) was added, followed by dropwise addition of 4-(dimethylamino)pyridine (DMAP, 0,53 g, 4.34 mmol, 0.01 eq.). The reaction was stirred overnight at rt. The solvent was then evaporated and the residue partitioned between ethyl acetate and water. The organic phase was washed with brine and the combined aqueous phases were extracted with ethyl acetate. The combined organic phases were dried over anh. Na_2SO_4; filtered and concentrated in vacuo. The residue was split into eight parts, each part was purified by fc (cyclohexane : ethyl acetate = 7:1, Ø = 8 cm, l = 28 cm, V = 100 mL). The mixed fractions were pooled and purified by fc to afford the product **51** as a colorless oil (R_f = 0.17, cyclohexane : ethyl acetate = 7:1), yield 140.2 g (84 % as a mixture of *α* to β-isomers 1:2).
$C_{24}H_{32}O_5Si$ (428.60 g/mol). Purity (HPLC: method B): > 99 % (t_R = 20.62 min, both isomers).
Exact mass (APCI): m/z calculated for $C_{24}H_{31}O_4Si$ [M-OH]$^+$ 411.1986, found 411.1998.
^{1}H-NMR (600 MHz, $CDCl_3$) δ (ppm) = 7.69-7.66 (m, 4H, *α*-2, 6-C*H*$_{Ph}$), 7.66-7.62 (m, 8H, β-2, 6-C*H*$_{Ph}$), 7.50-7.44 (m, 6H, *α*-3, 4, 5-C*H*$_{Ph}$), 7.44-7.38 (m, 12H, β-3, 4, 5-C*H*$_{Ph}$), 5.62 (d, *J* = 4.0 Hz, 1H, *α*-1-C*H*), 5.35 (s, 2H, β-1-C*H*), 5.30 (s, 0.1H, C*H*$_2$Cl$_2$, solvent: dichloromethane), 4.78 (dd, *J* = 6.3, 0.9 Hz, 1H, *α*-3-C*H*), 4.72 (dd, *J* = 5.9, 1.0 Hz, 2H, β-3-C*H*), 4.66 (dd, *J* = 6.3, 4.0 Hz, 1H, *α*-2-C*H*), 4.61 (d, *J* = 5.9 Hz, 2H, β-2-C*H*), 4.28 (td, *J* = 2.8, 0.9 Hz, 2H, β-4-C*H*), 4.15 (t, *J* = 2.4 Hz, 1H, *α*-4-C*H*), 3.82 (td, *J* = 11.1, 2.8 Hz, 3H, β-5-C*H*H, *α*-5-C*H*H), 3.66 (dd, *J* = 11.4, 2.7 Hz, 2H, β-5-CH*H*), 3.63 (dd, *J* = 11.2, 2.3 Hz, 1H, *α*-5-CH*H*), 2.17 (s, 0.3H, C*H*$_3$, solvent: acetone), 1.56 (s, 3H, *α*-C(C*H*$_3$)$_2$), 1.48 (s, 6H, β-C(C*H*$_3$)$_2$), 1.40 (s, 3H, *α*-C(C*H*$_3$)$_2$), 1.32 (s, 6H, β-C(C*H*$_3$)$_2$), 1.09 (s, 18H, β-C(C*H*$_3$)$_3$), 1.05 (s, 9H, *α*- C(C*H*$_3$)$_3$).
^{13}C-NMR (151 MHz, $CDCl_3$) δ (ppm) = 135.9 (4C, *α*-*C*-2, 6$_{Ph}$), 135.7 (8C, β-*C*-2, 6$_{Ph}$), 132.8, 132.5 (2C, *α*-*C*-1$_{Ph}$), 131.8, 131.7 (4C, β-*C*-1$_{Ph}$), 130.6, 130.4, 130.2, 130.1 (6C, *α*-*C*-3, 4, 5$_{Ph}$), 128.3, 128.2, 128.0 (12C, β-*C*-3, 4, 5$_{Ph}$), 113.1 (1C, *α*-*C*(CH_3)$_2$), 112.3 (2C, β-*C*(CH_3)$_2$), 103.6 (2C, β-*C*-1), 98.2 (1C, *α*-*C*-1), 87.5 (2C, β-*C*-2), 87.2 (2C, β-*C*-4), 82.1 (1C, *α*-*C*-3), 81.8 (2C, β-*C*-3), 81.4 (1C, *α*-*C*-4), 79.6 (1C, *α*-*C*-2), 66.2 (1C, *α*-*C*-5), 65.7 (2C, β-*C*-5), 27.0 (6C, β-C(*C*H$_3$)$_3$), 27.0 (3C, *α*-C(*C*H$_3$)$_3$), 26.6 (2C, β-C(*C*H$_3$)$_2$), 26.3 (1C, *α*-C(*C*H$_3$)$_2$), 25.1 (2C, β-C(*C*H$_3$)$_2$), 24.8 (1C, *α*-C(*C*H$_3$)$_2$), 19.3 (2C, β-*C*(CH_3)$_3$), 19.2 (1C, *α*-*C*(CH_3)$_3$).
FT-IR (neat) $\tilde{\nu}$ (cm^{-1}) = 3418 (O-H), 2959, 2936 (C-H$_{aliphat.}$), 1103, 1069 (C-O), 741, 702 (C-H$_{aromat.}$, out of plane).

(*R*)-2-[(*tert*-Butyldiphenylsilyl)oxy]-1-[(4*R*,5*S*)-2,2-dimethyl-5-vinyl-1,3-dioxolan-4-yl]ethan-1-ol (52)
The procedure was modified according to reference [16].Methyltriphenylphosphonium bromide (4.22 g, 11.8 mmol, 2 eq.) was suspended in dry tetrahydrofuran (THF, 27 mL) and cooled to 0 °C. Potassium *tert*-butoxide (1 mol/L in THF) (12 mL, 12 mmol, 2 eq.) was added and the resulting yellow slurry was stirred for 1 h at rt. The mixture was cooled to 0 °C again. Compound **51** (2.52 g, 5.88 mmol) was dissolved in THF (8 mL) and added to the slurry. The mixture was stirred for 3 h at 0 °C and 2 h at rt. The reaction was quenched with saturated NH_4Cl-solution and the mixture was extracted using ethyl acetate. The organic phase was washed with brine, dried over anh. Na_2SO_4 and concentrated in vacuo. The residue was purified by fc (cyclohexane : ethyl acetate = 10:1, Ø = 6 cm, l = 22 cm, V = 65 mL) to afford the product **52** as a colorless oil (R_f = 0.20, cyclohexane : ethyl acetate = 10:1), yield 2.34 g (93 %). $C_{25}H_{34}O_4Si$ (426.63 g/mol). Purity (HPLC: method B): 97 % (t_R = 20.60 min).
Exact mass (LC-MS-ESI): m/z calculated for $C_{25}H_{34}NaO_4Si$ [M+Na]$^+$ 449.2119, found 449.2082.

^{1}H-NMR (400 MHz, $CDCl_3$) δ (ppm) = 7.73-7.63 (m, 4H, 2, 6-CH_{Ph}), 7.48-7.35 (m, 6H, 3, 4, 5-CH_{Ph}), 6.01 (ddd, *J* = 17.1, 10.4, 6.7 Hz, 1H, C*H*=CH_2), 5.41 (ddd, *J* = 17.2, 1.9, 1.3 Hz, 1H, CH=C*H*H_{trans}), 5.30 (s, 0.2H, CH_2Cl_2, solvent: dichloromethane), 5.28 (ddd, *J* = 10.4, 1.9, 1.1 Hz, 1H, CH=CHH_{cis}), 4.70 (t, *J* = 6.5 Hz, 1H, 5-C$H_{dioxolane}$), 4.15 (dd, *J* = 8.9, 6.3 Hz, 1H, 4-C$H_{dioxolane}$), 3.86 (dd, *J* = 10.3, 3.2 Hz, 1H, 2-C*H*H), 3.80 (dd, *J* = 10.3, 5.5 Hz, 1H, 2-CH*H*), 3.71 (ddd, *J* = 8.8, 5.5, 3.2 Hz, 1H, 1-C*H*), 1.38 (s, 3H, $C(CH_3)_2$), 1.34 (s, 3H, $C(CH_3)_2$), 1.26 (t, *J* = 7.1 Hz, 0.1H, CH_2CH_3, solvent: ethyl acetate), 1.07 (s, 9H, $C(CH_3)_3$); The ^{1}H-NMR spectrum displays small impurities in the range of about 5 %.

2-[(*tert*-Butyldiphenylsilyl)oxy]-1-[(4*S*,5*S*)-2,2-dimethyl-5-vinyl-1,3-dioxolan-4-yl]ethan-1-one (53)

The procedure was modified according to reference [16].Dimethyl sulfoxide (DMSO, 1.35 mL, 19.0 mmol, 3.5 eq.) in CH_2Cl_2 (2 mL) was added dropwise to a solution of oxalyl chloride (2 mol/L in CH_2Cl_2, 4.5 mL, 9 mmol, 1.7 eq.) in CH_2Cl_2 (28 mL) at -78 °C and the mixture was stirred for 30 min. The alkene **52** (2.30 g, 5.39 mmol) was dissolved in CH_2Cl_2 (6 mL) and added to the solution. After 1.5 h of stirring at -78 °C, triethylamine (5 mL, 35.9 mmol, 6.6 eq.) was added and the reaction was allowed to warm to rt and was stirred at rt for 1 h. The reaction was cooled down to 0 °C and saturated NH_4Cl-solution was added. The mixture was extracted using CH_2Cl_2 and water, the organic phase dried over anh. Na_2SO_4 and concentrated. The residue was purified by fc (cyclohexane : ethyl acetate = 8:1, Ø = 6 cm, l = 22 cm, V = 65 mL) to afford the ketone **53** as a colorless oil (R_f = 0.22, cyclohexane : ethyl acetate = 8:1), yield 2.17 g (95 %). $C_{25}H_{32}O_4Si$ (424.61 g/mol).

Purity (HPLC: method B): 96 % (t_R = 21.12 min). Exact mass (LC-MS-ESI): m/z calculated for $C_{25}H_{32}NaO_4Si$ $[M+Na]^+$ 447.1962, found 447.1974.

^{1}H-NMR (400 MHz, $CDCl_3$) δ (ppm) = 7.69-7.60 (m, 4H, 2, 6-CH_{Ph}), 7.48-7.35 (m, 6H, 3, 4, 5-CH_{Ph}), 5.60-5.45 (m, 1H, C*H*=CH_2), 5.32 (ddd, *J* = 16.6, 1.7, 0.7 Hz, 1H, CH=C*H*H_{trans}) 5.12 (ddd, *J* = 9.8, 1.6, 0.7 Hz, 1H, CH=CHH_{cis}), 4.89-4.83 (m, 2H, 4-C$H_{dioxolane}$, 5-C$H_{dioxolane}$), 4.49 (dd, *J* = 18.8, 0.7 Hz, 1H, 2-C*H*H), 4.24 (dd, *J* = 18.9, 0.7 Hz, 1H, 2-CH*H*), 1.48 (s, 3H, $C(CH_3)_2$), 1.35 (s, 3H, $C(CH_3)_2$), 1.10 (s, 9H, $C(CH_3)_3$); The ^{1}H-NMR spectrum displays small impurities in the range of about 5 %.

1-[(*tert*-Butyldiphenylsilyl)oxy]-2-[(4*S*,5*S*)-2,2-dimethyl-5-vinyl-1,3-dioxolan-4-yl]but-3-en-2-ol (54)

The procedure was modified according to reference [16].The ketone **53** (113.6 g, 268 mmol) was dissolved in dry THF (1200 mL) under nitrogen atmosphere and cooled down to -78 °C. Vinyl magnesium bromide (1 mol/L in THF, 550 mL, 550 mmol, 2.1 eq.) was added dropwise at the same temperature. The reaction was stirred for 1.5 h and was then quenched with saturated NH_4Cl-solution and brine. After warming to rt, the mixture was extracted with ethyl acetate. The organic phase was dried over anh. Na_2SO_4 and concentrated in vacuo. The residue was split into two parts, each part was purified by fc (cyclohexane : ethyl acetate = 12:1, Ø = 8 cm, l = 28 cm, V = 100 mL). The mixed fractions were purified again using fc (cyclohexane : ethyl acetate = 16:1, Ø = 8 cm, l = 28 cm, V = 100 mL). The residual resulting mixed fractions were purified one last time by fc (cyclohexane : ethyl acetate = 20:1, Ø = 8 cm, l = 28 cm, V = 100 mL) to afford the tertiary alcohol **54** as a colorless oil (R_f = 0.29, cyclohexane : ethyl acetate = 12:1), yield 114.7 g (95 %). $C_{27}H_{36}O_4Si$ (452.67 g/mol). Purity (HPLC: method B): > 99 % (t_R = 21.94 min). Exact mass (APCI): m/z calculated for $C_{27}H_{37}0_4Si$ $[M+H]^+$ 453.2456, found 453.2466.

^{1}H-NMR (400 MHz, $CDCl_3$) δ (ppm) = 7.71-7.62 (m, 4H, 2, 6-CH_{Ph}), 7.49-7.34 (m, 6H, 3, 4, 5-CH_{Ph}), 6.18-6.00 (m, 2H, 3-C*H*=CH_2, C*H*=$CH_{2\ vinyl}$), 5.43 (dd, *J* = 17.5, 1.7 Hz, 1H, 4-C*H*H_{trans}), 5.29-5.20 (m, 2H, CH=C*H*$H_{trans\ vinyl}$, 4-C*H*H_{cis}), 5.12 (ddd, *J* = 10.2, 1.8, 0.9 Hz, 1H, CH=CH$H_{cis\ vinyl}$), 4.67 (ddt, *J* = 7.9, 6.9, 1.0 Hz, 1H, 5-C$H_{dioxolane}$), 4.45 (d, *J* = 6.9 Hz, 1H, 4-C$H_{dioxolane}$), 4.13 (q, *J* = 7.2 Hz, 0.1H, CH_2, solvent: ethyl acetate), 3.79 (d, *J* = 9.9 Hz, 1H, 1-C*H*H), 3.48 (d, *J* = 9.9 Hz, 1H, 1-CH*H*), 2.17 (s, 0.1H, CH_3, solvent: acetone), 2.05 (s, 0.1H, OCH_3, solvent: ethyl acetate), 1.49 (s, 3H, $C(CH_3)_2$), 1.43 (s, 1.3H, CH_2, solvent: cyclohexane), 1.39 (s, 3H, $C(CH_3)_2$), 1.26 (t, *J* = 7.1 Hz, 0.1H, CH_2CH_3, solvent: ethyl acetate), 1.07 (s, 9H, $C(CH_3)_3$).

(2*S*,3*S*)-1-{[(*tert*-Butyldiphenylsilyl)oxy]methyl}-2,3-*O*-isopropylidene-4-cyclopenten-1,2,3-triol (55)

The procedure was modified according to reference [16].The tertiary alcohol **54** (114.6 g, 253 mmol) was dissolved in CH_2Cl_2 (850 mL) and Grubbs catalyst M2 **53** (1.70 g, 1.79 mmol, 0.7 mol-%) was added. The mixture was stirred at rt for 7 d. The solvent was removed in vacuo and the residue was split into two parts. Each part was purified by fc (cyclohexane : ethyl acetate = 9:1 → 6:1 → 5:1, Ø = 8 cm, l = 28 cm, V = 100 mL). The mixed fractions were pooled and purified again via fc (cyclohexane : ethyl acetate = 19:1 → 5:1, Ø = 8 cm, l = 28 cm, V = 100 mL) to afford the product **55** as a light brown oil.

Light brown oil (R_f = 0.21, cyclohexane : ethyl acetate = 6:1), yield 101.6 g (94 %).$C_{25}H_{32}O_4Si$ (424.61 g/mol). Purity (HPLC: method B): > 99 % (t_R = 19.95 min). Exact mass (APCI): m/z calculated for $C_{25}H_{31}O_3Si$ $[M-OH]^+$ 407.2037, found 407.2036.

^{1}H-NMR (600 MHz, $CDCl_3$) δ (ppm) = 7.76-7.66 (m, 4H, 2, 6-C*H*$_{Ph}$), 7.47-7.36 (m, 6H, 3, 4, 5-C*H*$_{Ph}$), 5.98 (dd, *J* = 5.8, 1.7 Hz, 1H, 5-C*H*), 5.74 (dq, *J* = 5.8, 0.8 Hz, 1H, 4-C*H*), 5.34 (ddd, *J* = 5.3, 1.8, 0.9 Hz, 1H, 3-C*H*), 4.54 (dt, *J* = 5.4, 0.8 Hz, 1H, 2-C*H*), 4.02 (d, *J* = 10.0 Hz, 1H, OC*H*H), 3.70 (d, *J* = 10.1 Hz, 1H, OCH*H*), 2.17 (s, 0.2H, C*H*$_3$, solvent: acetone), 1.33 (s, 3H, C(C*H*$_3$)$_2$), 1.27 (s, 3H, C(C*H*$_3$)$_2$), 1.09 (s, 9H, C(C*H*$_3$)$_3$); The ^{1}H-NMR spectrum displays small impurities in the range of about 5 %.

^{13}C-NMR (151 MHz, $CDCl_3$) δ (ppm) = 135.9 (1C, *C*-4), 135.9, 135.7 (4C, *C*-2, 6$_{Ph}$), 134.6 (1C, *C*-5), 133.2, 133.0 (2C, *C*-1$_{Ph}$), 130.0, 130.0 (2C, *C*-4$_{Ph}$), 127.9, 127.8 (4C, *C*-3, 5$_{Ph}$), 112.2 (1C, *C*(CH$_3$)$_2$), 85.2 (1C, *C*-1), 85.0 (1C, *C*-2), 84.8 (1C, *C*-3), 65.9 (1C, OCH$_2$), 27.6 (1C, C(CH$_3$)$_2$), 27.0 (3C, C(CH$_3$)$_3$), 26.3 (1C, C(CH$_3$)$_2$), 19.5 (1C, *C*(CH$_3$)$_3$).

FT-IR (neat) $\tilde{\nu}$ (cm^{-1}) = 3071 (v C-H$_{aromat.}$), 2959, 2932 (C-H$_{aliphat.}$), 1107, 1065 (C-O), 741, 702 (C-H$_{aromat.}$, out of plane).

(4*R*,5*R*)-3-{[(*tert*-Butyldiphenylsilyl)oxy]methyl}-4,5-dihydroxy-4,5-*O*-isopropylidene-2-cyclopenten-1-one (56)

The procedure was modified according to reference [16].Compound **55** (50.9 g, 120 mmol) was dissolved in dimethylformamide (DMF, 0.43 L). 4 Å Molecular sieves (100.4 g) and pyridinium dichromate (138.8 g, 369 mmol, 3 eq.) were added. The mixture was stirred for 2 d at rt. Approximately 100 mL of the solvent were evaporated and ethyl acetate was added to the mixture. The slurry was filtered portion wise through a pad of silica gel and washed with ethyl acetate. The solvent was evaporated. Ethyl acetate was added to the residue and it was filtered through a paper filter. The solvent was evaporated again and the residue was purified by fc (cyclohexane : ethyl acetate = 8:1, Ø = 8 cm, l = 28 cm, V = 100 mL). The mixed fractions were pooled and purified again by fc (cyclohexane : ethyl acetate = 12:1 → 8:1, Ø = 8 cm, l = 28 cm, V = 100 mL) to afford the ketone **56** as a colorless oil (R_f = 0.17, cyclohexane : ethyl acetate = 7:1), yield 23.2 g (46 %). $C_{25}H_{30}O_4Si$ (422.60 g/mol). Purity (HPLC: method B): > 99 % (t_R = 20.61 min).

Exact mass (APCI): m/z calculated for $C_{25}H_{31}O_4Si$ $[M+H]^+$ 423.1986, found 423.1985.

^{1}H-NMR (600 MHz, DMSO-*d*$_6$) δ (ppm) = 7.66-7.63 (m, 2H, 2, 6-C*H*$_{Ph}$), 7.63-7.60 (m, 2H, 2, 6-C*H*$_{Ph}$), 7.51-7.41 (m, 6H, 3, 4, 5-C*H*$_{Ph}$), 6.16 (t, *J* = 1.9 Hz, 1H, 2-C*H*), 5.75 (s, 0.3H, C*H*$_2$Cl$_2$, solvent: dichloromethane), 5.14 (d, *J* = 5.5 Hz, 1H, 5-C*H*), 4.70 (ddd, *J* = 18.8, 2.1, 0.9 Hz, 1H, OC*H*H), 4.59-4.54 (m, 1H, OCH*H*), 4.53 (d, *J* = 5.6 Hz, 1H, 4-C*H*), 1.39 (s, 0.2H, C*H*$_2$, solvent: cyclohexane), 1.28 (s, 3H, C(C*H*$_3$)$_2$), 1.21 (s, 3H, C(C*H*$_3$)$_2$), 1.03 (s, 9H, C(C*H*$_3$)$_3$); The ^{1}H-NMR spectrum displays small impurities in the range of about 5 %.

^{13}C-NMR (151 MHz, DMSO-*d*$_6$) δ (ppm) = 201.4 (1C, *C*-1), 177.1 (1C, *C*-3), 135.0, 134.9 (2C, *C*-2, 6$_{Ph}$), 132.2, 132.2 (2C, *C*-1$_{Ph}$), 130.1 (2C, *C*-4$_{Ph}$), 128.1 (4C, *C*-3, 5$_{Ph}$), 126.6 (1C, *C*-2), 114.1 (1C, *C*(CH$_3$)$_2$), 77.4 (1C, *C*-4), 77.2 (1C, *C*-5), 62.1 (1C, OCH$_2$), 54.9 (0.2C, *C*H$_2$Cl$_2$, solvent: dichloromethane), 27.1 (1C, C(CH$_3$)$_2$), 26.5 (3C, C(CH$_3$)$_3$), 26.0 (1C, C(CH$_3$)$_2$), 18.8 (1C, *C*(CH$_3$)$_2$).

FT-IR (neat) $\tilde{\nu}$ (cm^{-1}) = 3071 (v C-H$_{aromat.}$), 2959, 2928 (C-H$_{aliphat.}$), 1717 (C=O), 1103, 1065 (C-O), 741, 702 (C-H$_{aromat.}$, out of plane).

(1*S*,2*S*,3*R*)-4-{[(*tert*-Butyldiphenylsilyl)oxy]methyl}-2,3-*O*-isopropylidene-4-cyclopenten-1,2,3-triol (57)

The procedure was modified according to reference [16].The ketone **56** (10.4 g, 24.5 mmol) was dissolved in CH_3OH (66 mL) and $CeCl_3$ x 7 H_2O (11.1 g, 29.7 mmol, 1.2 eq.) was added. The mixture was stirred at rt until it became a clear solution. The reaction was cooled to 0 °C and $NaBH_4$ (1.50 g, 39.7 mmol, 1.6 eq.) was added in small portions. Afterwards the mixture was stirred at 0 °C for 30 min. Acetic acid was added to adjust the pH value to 5. The reaction was extracted using diethylether and the organic phase was washed with brine and dried over anh. Na_2SO_4 and concentrated in vacuo. The residue was purified by fc (cyclohexane : ethyl acetate = 9:1, Ø = 8 cm, l = 28 cm, V = 100 mL) to afford the alcohol **57** as a colorless oil (R_f = 0.15, cyclohexane : ethyl acetate = 7:1), yield 9.21 g (88 %). $C_{25}H_{32}O_4Si$ (424.61 g/mol). Purity (HPLC: method B): > 99 % (t_R = 19.49 min).

Exact mass (APCI): m/z calculated for $C_{25}H_{33}O_4Si$ $[M+H]^+$ 425.2143, found 425.2124.

^{1}H-NMR (600 MHz, DMSO-*d*$_6$) δ (ppm) = 7.67-7.59 (m, 4H, 2, 6-C*H*$_{Ph}$), 7.51-7.39 (m, 6H, 3, 4, 5-C*H*$_{Ph}$), 5.75 (s, 0.3H, C*H*$_2$Cl$_2$, solvent: dichloromethane), 5.68 (dt, *J* = 1.9, 0.9 Hz, 1H, 5-C*H*), 4.80 (d, *J* = 5.4 Hz, 1H, 3-C*H*), 4.61 (td, *J* = 5.3, 0.8 Hz, 1H, 2-C*H*), 4.55 (d, *J* = 8.1 Hz, 1H, O*H*), 4.47 (ddq, *J* = 9.4, 4.5, 1H, 1-C*H*), 4.28 (t, 2.2 Hz, 2H, OC*H*$_2$), 1.39 (s, 0.7H, C*H*$_2$, solvent: cyclohexane), 1.25 (s, 6H, C(C*H*$_3$)$_2$), 1.01 (s, 9H, C(C*H*$_3$)$_3$).

^{13}C-NMR (151 MHz, DMSO-*d*$_6$) δ (ppm) = 143.5 (1C, *C*-4), 134.9 (4C, *C*-2, 6$_{Ph}$), 132.8 (2C, *C*-1$_{Ph}$), 129.9 (2C, *C*-4$_{Ph}$), 129.3 (1C, *C*-5), 127.9 (4C, *C*-3, 5$_{Ph}$), 110.9 (1C, *C*(CH$_3$)$_2$), 82.0 (1C, *C*-3), 78.5 (1C, *C*-2), 72.5 (1C, *C*-1), 60.6 (1C, OCH$_2$), 54.9 (0.2C, *C*H$_2$Cl$_2$, solvent: dichloromethane), 27.5 (1C, C(CH$_3$)$_2$), 26.6 (4C, C(*C*H$_3$)$_2$, C(*C*H$_3$)$_3$) 26.3 (0.4C, *C*H$_2$, solvent: cyclohexane), 18.8 (1C, *C*(CH$_3$)$_3$).

FT-IR (neat) $\tilde{\nu}$ (cm^{-1}) = 3549 (O-H), 3071 (ν C-H$_{aromat.}$), 2931 (C-H$_{aliphat.}$), 1103, 1049 (C-O), 741, 702 (C-H$_{aromat.}$, out of plane).

(1*R*,2*R*,3*S*,4*S*,5*S*)-1-{[(*tert*-Butyldiphenylsilyl)oxy]methyl}-2,3-*O*-isopropylidenebicyclo[3.1.0]hexan-2,3,4-triol (4)

The procedure was modified according to reference [16].Compound **57** (1.01 g, 2.37 mmol) was dissolved in dry CH_2Cl_2 (13 mL) under nitrogen atmosphere. The reaction was cooled down to -18 °C with an ice/salt bath. Diethylzinc (1 mol/L in hexane, 2.60 mL, 2.60 mmol, 1.1 eq.) was added dropwise and the mixture stirred for 15 min. Diidomethane (0.22 mL, 2.73 mmol, 1.15 eq.) in dry CH_2Cl_2 (1.6 mL) was also added dropwise and the reaction was stirred for another 15 min. Both steps were repeated a second time. Then diethylzinc (1 mol/L in hexane, 2.60 mL, 2.60 mmol, 1.1 eq.) was added for the third time. After stirring for 15 min at -18 °C the reaction was allowed to warm to rt and stirred overnight. The reaction was quenched with saturated NH_4Cl-solution and was extracted five times with CH_2Cl_2. The organic phase was dried over anh. Na_2SO_4 and concentrated in vacuo. The residue was purified by fc (cyclohexane : ethyl acetate = 7:1, Ø = 5 cm, l = 22 cm, V = 30 mL) to afford the product **4** as a colorless oil (R_f = 0.20, cyclohexane : ethyl acetate = 5:1), yield 0.90 g (86 %). $C_{26}H_{34}O_4Si$ (438.64 g/mol). Purity (HPLC: method B): > 99 % (t_R = 18.94 min). Exact mass (APCI): m/z calculated for $C_{23}H_{27}O_2Si$ [M-OH, -CO(CH$_3$)$_2$]$^+$ 363.1775, found 363.1777.

^{1}H-NMR (600 MHz, CDCl$_3$) δ (ppm) = 7.66-7.60 (m, 4H, 2, 6-C*H*$_{Ph}$), 7.46-7.34 (m, 6H, 3, 4, 5-C*H*$_{Ph}$), 5.00 (dd, *J* = 6.9, 1.2 Hz, 1H, 2-C*H*), 4.54 (td, *J* = 6.9, 0.8 Hz, 1H, 3-C*H*), 4.45 (dt, *J* = 9.6, 6.1 Hz, 1H, 4-C*H*), 4.12 (q, *J* = 7.2 Hz, 0.2H, C*H*$_2$, solvent: ethyl acetate), 4.07 (d, *J* = 11.0 Hz, 1H, OC*H*H), 3.29 (d, *J* = 11.0 Hz, 1H, OCH*H*), 2.33 (d, *J* = 9.7 Hz, 1H, O*H*), 2.04 (s, 0.3H, OC*H*$_3$, solvent: ethyl acetate), 1.61 (dt, *J* = 9.3, 4.9 Hz, 1H, 5-C*H*), 1.54 (s, 3H, C(C*H*$_3$)$_2$), 1.31 (s, 3H, C(C*H*$_3$)$_2$), 1.26 (t, *J* = 7.1 Hz, 0.5H, CH$_2$C*H*$_3$, solvent: ethyl acetate), 1.09 (t, *J* = 5.0 Hz, 1H, 6-C*H*H), 1.05 (s, 9H, C(C*H*$_3$)$_3$), 0.54 (ddt, *J* = 8.8, 5.3, 1.1 Hz, 1H, 6-CH*H*).

^{13}C-NMR (151 MHz, CDCl$_3$) δ (ppm) = 135.7 (4C, *C*-2, 6$_{Ph}$), 133.8, 133.7 (2C, *C*-1$_{Ph}$), 129.9 (2C, *C*-4$_{Ph}$), 127.8 (4C, *C*-3, 5$_{Ph}$), 113.0 (1C, *C*(CH$_3$)$_2$), 81.3 (1C, *C*-2), 79.9 (1C, *C*-3), 71.2 (1C, *C*-4), 65.4 (1C, OCH$_2$), 35.7 (1C, *C*-1), 33.0 (1C, *C*-5), 27.0 (3C, C(*C*H$_3$)$_3$), 26.3 (1C, C(*C*H$_3$)$_2$), 24.8 (1C, C(*C*H$_3$)$_2$), 19.4 (1C, *C*(CH$_3$)$_3$), 10.5 (1C, *C*-6).

FT-IR (neat) $\tilde{\nu}$ (cm^{-1}) = 2932, 2859 (C-H$_{aliphat.}$), 1470 (C=C$_{aromat.}$), 1107, 1080, 1042 (C-O), 741, 702 (C-H$_{aromat.}$, out of plane).

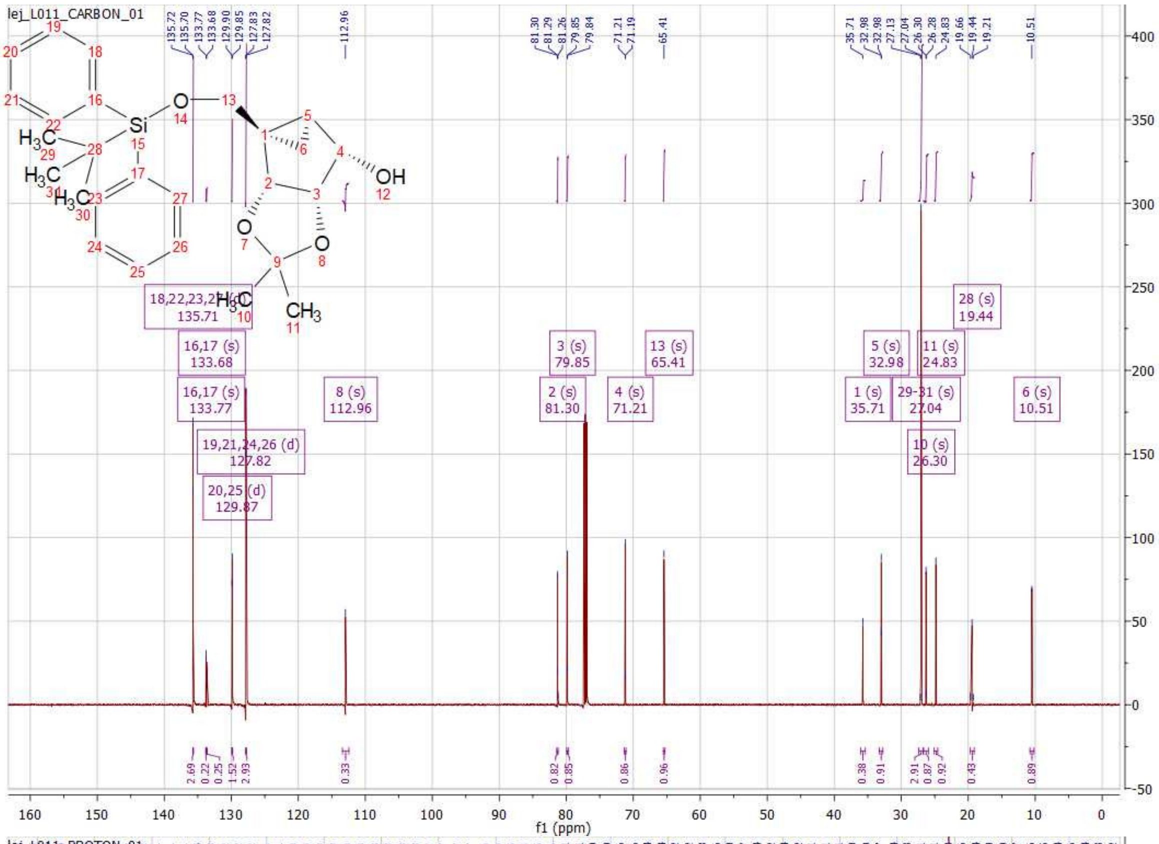
lej_L011_CARBON_01
18,22,23,27 (d) 135.71
16,17 (s) 133.68
16,17 (s) 133.77
19,21,24,26 (d) 127.82
20,25 (d) 129.87
8 (s) 112.96
3 (s) 79.85
2 (s) 81.30
4 (s) 71.21
13 (s) 65.41
5 (s) 32.98
1 (s) 35.71
11 (s) 24.83
29-31 (s) 27.04
10 (s) 26.30
28 (s) 19.44
6 (s) 10.51
f1 (ppm)

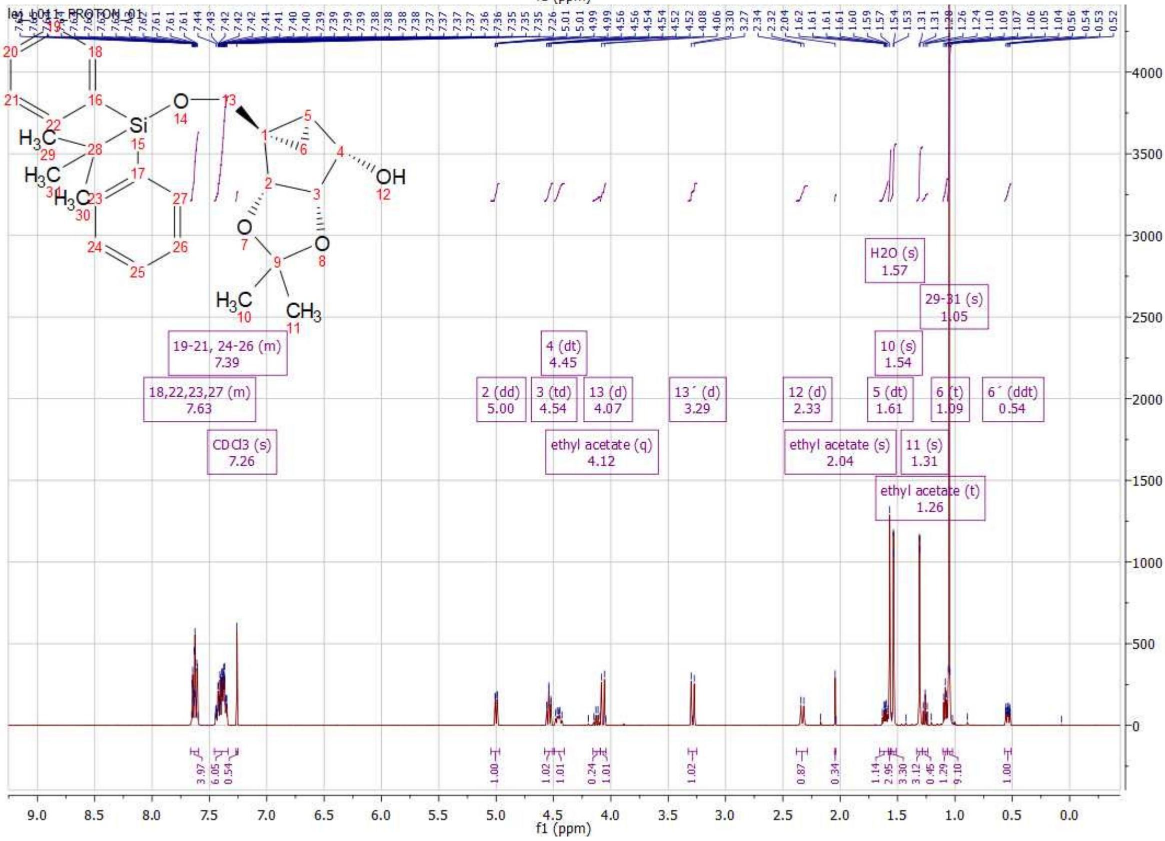
lej_L011_PROTON_01
H2O (s) 1.57
29-31 (s) 1.05
19-21, 24-26 (m) 7.39
18,22,23,27 (m) 7.63
CDCl3 (s) 7.26
4 (dt) 4.45
2 (dd) 5.00
3 (td) 4.54
13 (d) 4.07
13´ (d) 3.29
ethyl acetate (q) 4.12
12 (d) 2.33
ethyl acetate (s) 2.04
10 (s) 1.54
5 (dt) 1.61
6 (t) 1.09
6´ (ddt) 0.54
11 (s) 1.31
ethyl acetate (t) 1.26
f1 (ppm)

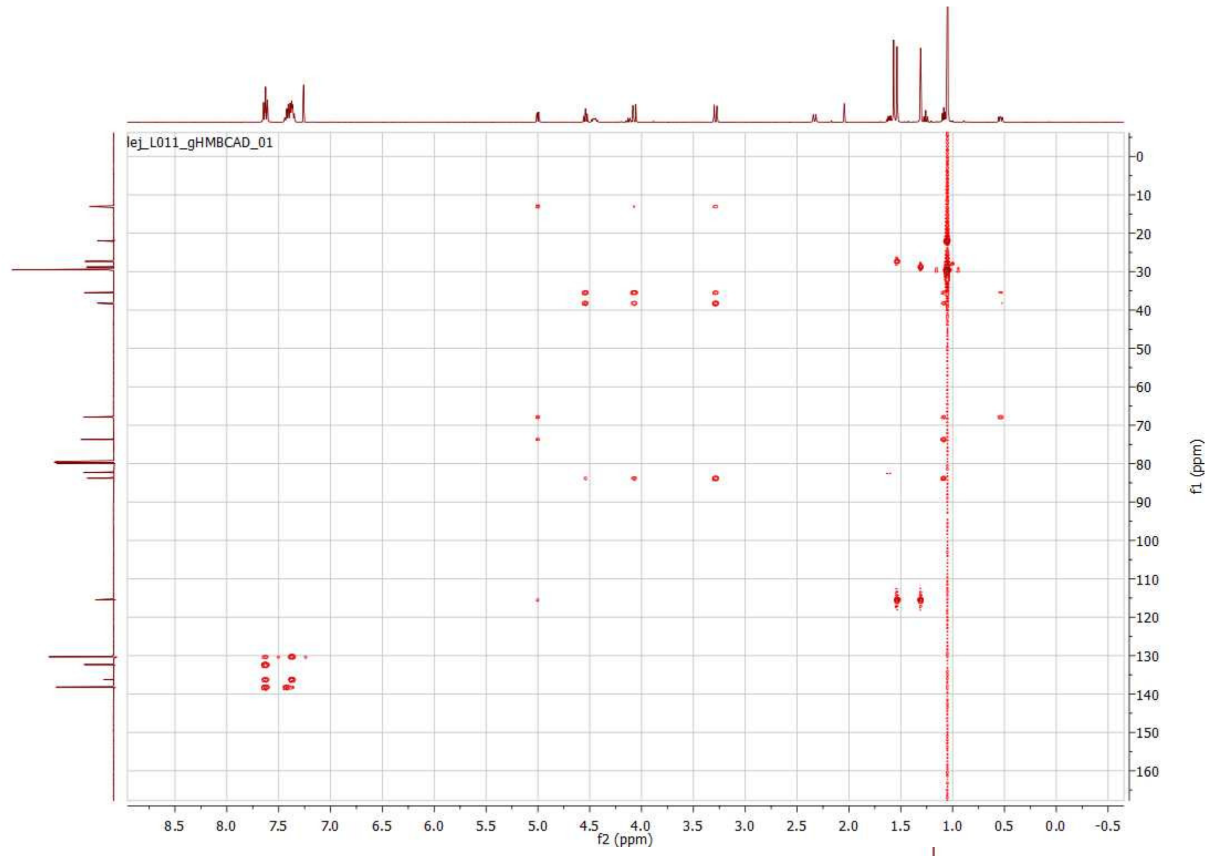
lej_L011_gHMBCAD_01
f2 (ppm)
f1 (ppm)

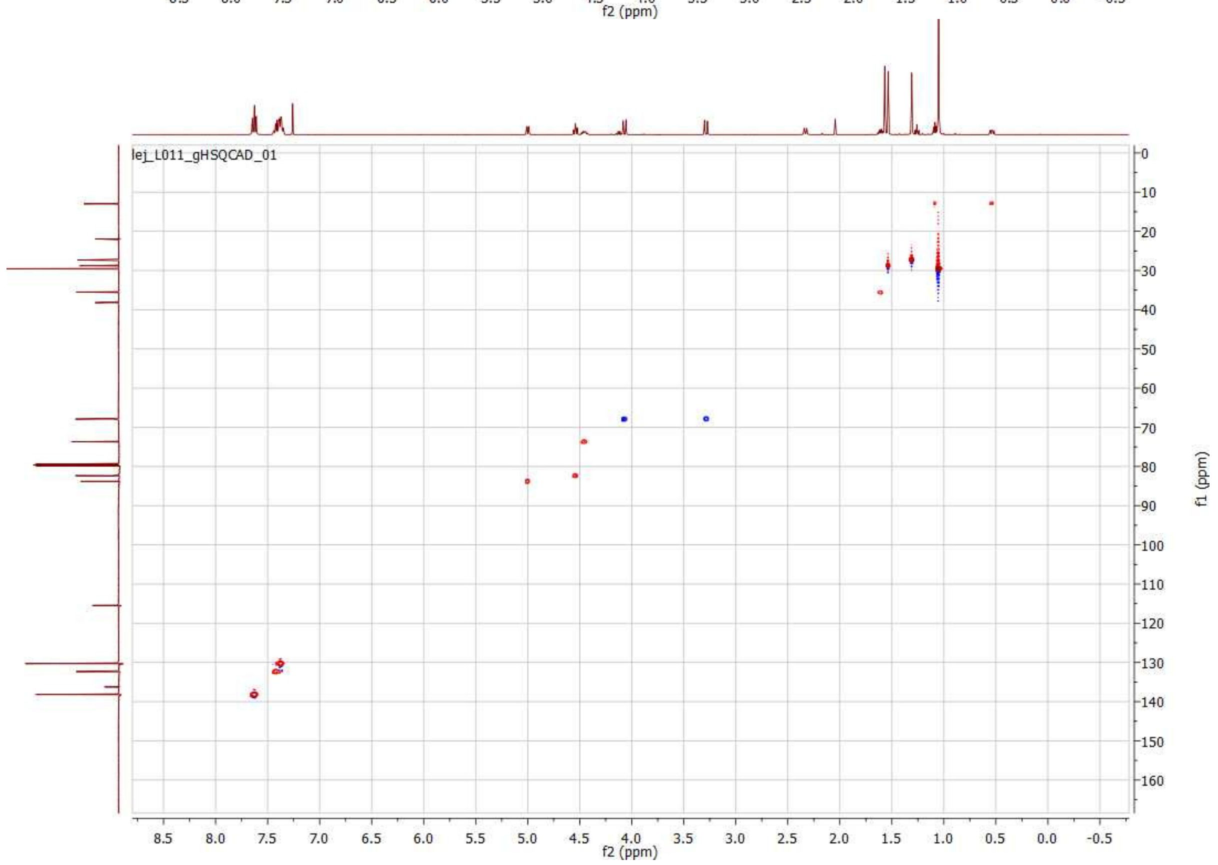
lej_L011_gHSQCAD_01
f2 (ppm)
f1 (ppm)

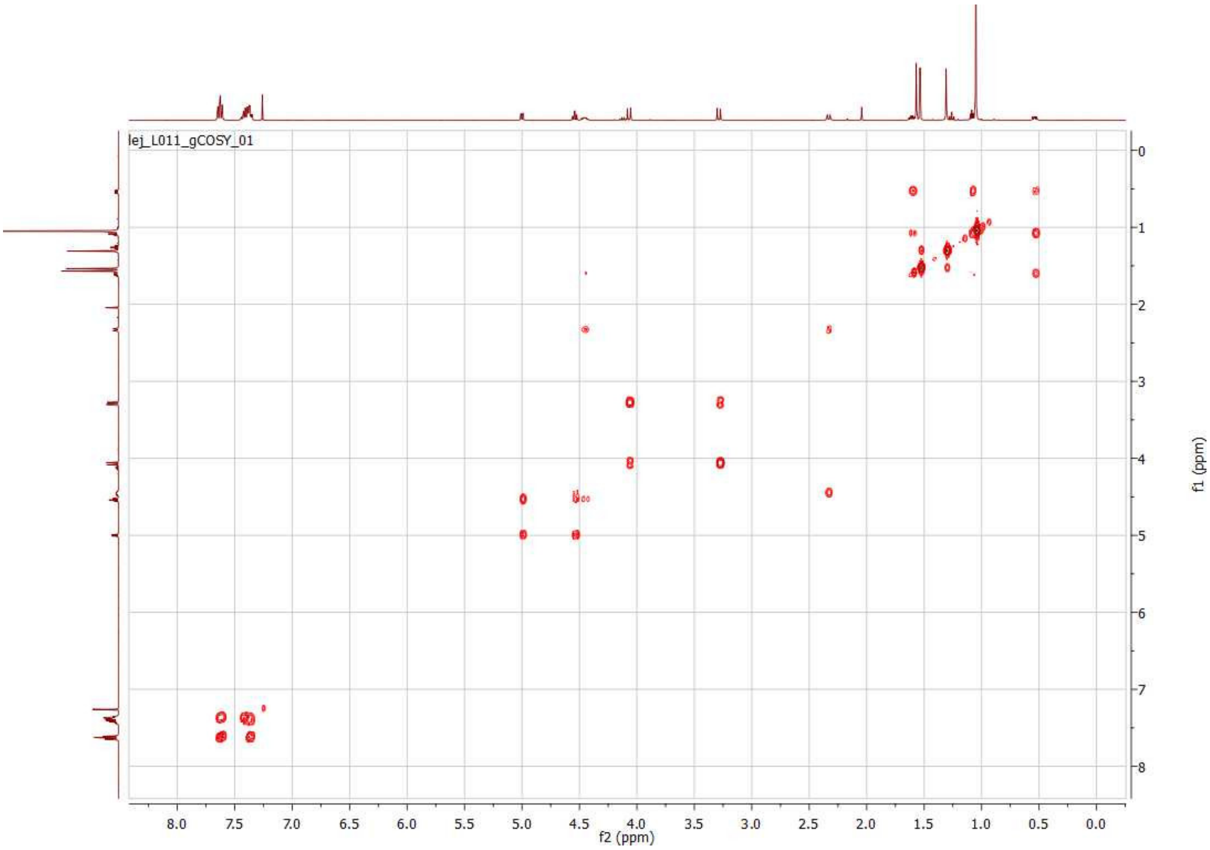
lej_L011_gCOSY_01
f1 (ppm)
f2 (ppm)
8.0 7.5 7.0 6.5 6.0 5.5 5.0 4.5 4.0 3.5 3.0 2.5 2.0 1.5 1.0 0.5 0.0
0 1 2 3 4 5 6 7 8

Mass Spectrum SmartFormula Report

Analysis Info

Analysis Name	\\pz-messdaten\MS-Daten\IPMC\routine\APCI\18_11\WLJ_JL011Rs.d	Acquisition Date	06.11.2018 14:38:35
Method	APCI_directprobe_positiv.m	Operator	Meiners
Sample Name	JL011Rs	Instrument / Ser#	micrOTOF-Q 10252
Comment	Lemmerhirt<br>APCI-Direkt<br>Kalibration mit Fettsaeureestern		

Acquisition Parameter

Source Type	APCI	Ion Polarity	Positive	Set Nebulizer	0.7 Bar
Focus	Not active	Set Capillary	4000 V	Set Dry Heater	200 °C
Scan Begin	60 m/z	Set End Plate Offset	-500 V	Set Dry Gas	3.0 l/min
Scan End	1000 m/z	Set Collision Cell RF	130.0 Vpp	Set Divert Valve	Waste

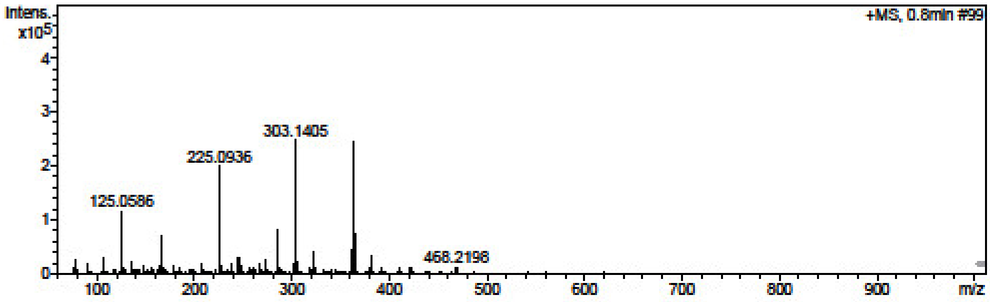

Meas. m/z	#	Formula	Score	m/z	err [mDa]	err [ppm]	mSigma	rdb	e⁻ Conf	N-Rule
363.1777	1	C 23 H 27 O 2 Si	100.00	363.1775	-0.3	-0.7	7.2	11.5	even	ok
	2	C 27 H 23 O	6.63	363.1743	-3.4	-9.3	27.0	16.5	even	ok
	3	C 26 H 23 N 2	0.01	363.1856	7.8	21.6	31.2	16.5	even	ok
	4	C 22 H 23 N 2 O 3	0.01	363.1703	-7.4	-20.4	46.6	12.5	even	ok
	5	C 21 H 23 N 4 O 2	2.29	363.1816	3.8	10.5	49.2	12.5	even	ok

Bruker Compass DataAnalysis 4.0 printed: 16.11.2018 15:57:51 Page 1 of 1
